# The impact of community health worker-led home delivery of antiretroviral therapy on virological suppression: a non-inferiority cluster-randomized health systems trial in Dar es Salaam, Tanzania

**DOI:** 10.1186/s12913-017-2032-7

**Published:** 2017-02-22

**Authors:** Pascal Geldsetzer, Joel M. Francis, Nzovu Ulenga, David Sando, Irene A. Lema, Eric Mboggo, Maria Vaikath, Happiness Koda, Sharon Lwezaula, Janice Hu, Ramadhani A. Noor, Ibironke Olofin, Elysia Larson, Wafaie Fawzi, Till Bärnighausen

**Affiliations:** 1 Management and Development for Health, Plot #802, Mwai Kibaki Road, Mikocheni, Dar es Salaam, Tanzania; 2 Department of Global Health and Population, Harvard T.H. Chan School of Public Health, 665 Huntington Avenue, Boston, MA 02115 USA; 3 National AIDS Control Program, Lithuli Street, P.O. Box 11857, Dar es Salaam, Tanzania; 4 Department of Nutrition, Harvard T.H. Chan School of Public Health, 677 Huntington Avenue, Boston, MA 02115 USA; 5 National Institute for Medical Research, 3 Barack Obama Drive, 11101 Dar es Salaam, Tanzania; 6 Duke University School of Medicine, Duke University, 8 Duke University Medical Center Greenspace, Durham, NC 27703 USA; 7 Institute for Public Health, Faculty of Medicine, Heidelberg University, Im Neuenheimer Feld 130.3, 69120 Heidelberg, Germany; 8 Africa Health Research Institute, Mtubatuba 3935, KwaZulu-Natal, South Africa; 9 Department of Epidemiology, Harvard T.H. Chan School of Public Health, 677 Huntington Avenue, Boston, MA 02115 USA; 10 Africa Academy for Public Health (AAPH), Plot #802, Mwai Kibaki Road, Dar es Salaam, Tanzania

**Keywords:** Antiretroviral therapy, HIV, Community health workers, Nutrition counseling, Food production, Adherence, Retention, Healthcare expenditure

## Abstract

**Background:**

Home delivery of antiretroviral therapy (ART) by community health workers (CHWs) may improve ART retention by reducing the time burden and out-of-pocket expenditures to regularly attend an ART clinic. In addition, ART home delivery may shorten waiting times and improve quality of care for those in facility-based care by decongesting ART clinics. This trial aims to determine whether ART home delivery for patients who are clinically stable on ART combined with facility-based care for those who are not stable on ART is non-inferior to the standard of care (facility-based care for all ART patients) in achieving and maintaining virological suppression.

**Methods:**

This is a non-inferiority cluster-randomized trial set in Dar es Salaam, Tanzania. A cluster is one of 48 healthcare facilities with its surrounding catchment area. 24 clusters were randomized to ART home delivery and 24 to the standard of care. The intervention consists of home visits by CHWs to provide counseling and deliver ART to patients who are stable on ART, while the control is the standard of care (facility-based ART and CHW home visits without ART home delivery). In addition, half of the healthcare facilities in each study arm were randomized to standard counseling during home visits (covering family planning, prevention of HIV transmission, and ART adherence), and half to standard plus nutrition counseling (covering food production and dietary advice). The non-inferiority design applies to the endpoints of the ART home delivery trial; the primary endpoint is the proportion of ART patients at a healthcare facility who are virally suppressed at the end of the study period. The margin of non-inferiority for this primary endpoint was set at nine percentage points.

**Discussion:**

As the number of ART patients in sub-Saharan Africa is expected to rise, this trial provides causal evidence on the effectiveness of a home-based care model that could decongest ART clinics and reduce patients’ healthcare expenditures. More broadly, this trial will inform the increasing policy interest in task-shifting of chronic disease care from facility- to community-based healthcare workers.

**Trial registration:**

ClinicalTrials.gov: NCT02711293. Registration date: 16 March 2016.

**Electronic supplementary material:**

The online version of this article (doi:10.1186/s12913-017-2032-7) contains supplementary material, which is available to authorized users.

## Background

An estimated 37% of the 24.7 million people living with the human immunodeficiency virus (HIV) in sub-Saharan Africa (SSA) were receiving antiretroviral therapy (ART) in 2015 [1, 2]. Since the advent of antiretroviral drugs, the World Health Organization (WHO) has gradually increased the recommended threshold for ART initiation from a CD4-cell count of less than 200 cells/μL in 2006 [3], to less than 350 cells/μL in 2010 [4], and less than 500 cells/μL in 2013 [5]. Most recently, in 2015, the WHO has recommended immediate ART for all people living with HIV, irrespective of CD4 count [6]. As countries expand ART eligibility– and because ART increases the life expectancy of people living with HIV – the number of people needing ART in SSA is likely to increase substantially over the coming decades [6, 7]. However, the financial, human and physical resources available to deliver ART are unlikely to grow in proportion to this increase. These developments call for new models of care that increase the capacity and efficiency of delivering ART without reducing quality of care [8].

### The importance of ART retention and adherence

The benefits from the scale-up in ART coverage will critically depend on life-long ART retention and adherence. A meta-analysis pooling ART adherence data from over 30,000 adult patients in 84 observational studies across 20 countries found that 38% of patients took less than 90% of prescribed ART doses [9]. Similarly, previous analyses by our team in a cohort of over 44,000 patients in Dar es Salaam’s adult HIV treatment and care program have found low ART retention and adherence. More specifically, we found that 39% of adults on ART were lost to follow-up within 12 months of initiation (unpublished data). In addition, 19% were non-adherent to ART [10] (as defined by non-compliance with scheduled ART pickup visits of greater than 5%) at any given point in time, with the risk of non-adherence increasing with duration on ART. Worryingly, the risk of non-adherence also rose independently with increasing calendar year, with the relative risk (RR) of non-adherence being 2.0 (95% confidence interval: 1.9-2.1) in 2010 compared to the reference year 2004. Poor adherence is not only likely to lead to treatment failure and resulting morbidity and mortality, but also increases the risk of HIV transmission and, crucially, the development of resistant HIV strains [11–13]. Increasing ART resistance may narrow future antiretroviral drug options, and thus reduce ART access and increase the cost of effective HIV treatment as programs have to move to more expensive second and third-line regimens.

### Reasons for community health worker-led home delivery of ART to improve retention and adherence

In a large qualitative study in three urban settings in sub-Saharan Africa, including Dar es Salaam, Ware et al. found that the main unintentional reason for missed ART clinic visits was a lack of time due to other, often unexpected, events in a patient’s life [14]. In addition, many studies have identified the cost to patients of attending ART clinics (not just expenses on user fees but also on transport, food, and lost income [14–18]), transport-related factors [19], and long clinic waiting times [14] as being important barriers to ART retention. Given that the delivery of ART at home through community health workers (CHWs) would overcome many of these barriers, CHWs have the potential to significantly improve ART retention and adherence.

An additional benefit of a home-based approach to ART arises from the resulting reduced patient load at ART clinics, which may decrease waiting times and improve quality of care as facility-based healthcare workers have more time available per patient. The home delivery of ART by CHWs is, therefore, not merely an intervention aimed at improving ART retention and adherence but also a measure that can shift care from more highly to less well-trained health workers. Such task-shifting measures may therefore alleviate the severe shortage of human resources for health in SSA, which is a central barrier to attaining universal coverage of HIV services [20, 21]. The WHO has identified 313 tasks, which are essential for the prevention of HIV transmission, identification of HIV-infected individuals, provision of basic HIV-related clinical management, and initiation and maintenance of patients on ART. The WHO recommends that 115 of these tasks, including the dispensing of ART, can be performed by CHWs, highlighting the immense potential of task-shifting for HIV-related care [22].

### The current evidence base for home delivery of ART by community health workers

A systematic review of health service delivery for ART provision identified two randomized trials, which evaluated ART home-delivery programs [23]. Both of these trials randomized geographical areas around one ART clinic. The first trial was set in rural Uganda and randomized areas to either home delivery of ART by field officers, or standard facility-based ART [24]. The participants were patients newly initiated on ART. The trial found no difference between study arms in the rate of virological failure or mortality, neither after six months [24] nor at 36-months follow-up [25]. The median expense to patients in terms of transport costs, food, child care, and lost work time due to ART utilization was higher in the facility-based group than in the home-based group at US$60 versus US$29 in the first year, and US$54 versus US$18 in subsequent years. In addition, the median cost to the health system per patient per year was somewhat lower in the CHW group (US$793) than in the facility-based group (US$838) as the increased transport costs for CHWs were offset by patients’ reduced clinic attendance. In another study, the same cohort of patients receiving ART home delivery was compared with ART patients who attended a physician-staffed hospital in an urban sub-district of Uganda [26, 27]. While comparability of the two cohorts is limited by the observational study design, the study found that community-based participants were more likely to achieve viral suppression (after adjusting for CD4-count at baseline and socio-demographic characteristics), and there was no difference in all-cause mortality. The second randomized trial was carried out in rural Kenya and, similar to the Uganda study, found no difference in the percentage of patients with an undetectable viral load, mean CD4-count, incidence of opportunistic infections, and change in ART regimen between stable ART patients who received ART from CHWs during home visits, as compared to patients randomized to standard facility-based ART [28].

This trial differs in several crucial aspects from the two studies described above. Firstly, this trial is the first to evaluate ART home delivery in an urban setting. Secondly, this is a health systems trial, which implements the intervention directly into the routine healthcare system. Both the Uganda and Kenya trial randomized geographic areas around one clinic run by a non-governmental organization [24, 28], while this study is being implemented at all healthcare facilities in Dar es Salaam that offer ART and have an affiliated team of public-sector CHWs. In addition, while the Uganda trial trained a new cadre of field officers to deliver ART by motorbike and the Kenya trial trained ART patients at the clinic to act as community care coordinators, this trial is utilizing a large existing public-sector CHW program in Dar es Salaam. Thirdly, this trial includes a large number (24) of healthcare facilities in each study arm, while both the Uganda and Kenya trial implemented the intervention at only one clinic. Aside from external validity concerns arising from a study carried out in a single clinic, an important disadvantage of drawing the intervention and control group from the same healthcare facility is that ART home delivery is likely to have affected the care provided to the control group, as the shifting of patients to community-based care substantially reduced the patient volume at the facility. Such shifts may have resulted in the control group being a poor counterfactual. Finally, both the Uganda and Kenya trial were unable to rigorously assess the effect of shifting patients to the community on routine facility-based ART. This trial, on the other hand, will be able to compare patient satisfaction, time spent by nurses and physicians with ART patients, and clinicians’ job satisfaction between the 24 intervention and 24 control facilities.

This non-inferiority cluster-randomized trial evaluates the feasibility and effectiveness of CHW-led home delivery of ART in the public-sector healthcare system of Dar es Salaam, the largest city in Eastern Africa. More specifically, this study aims to determine whether a differentiated ART care model (ART home delivery for patients who are clinically stable on ART and standard facility-based care for those who are not stable on ART) is non-inferior to the standard of care (facility-based care for all ART patients) in achieving and maintaining virological suppression.

## Methods/design

### Study setting

The study will be implemented in all three municipalities (Ilala, Kinondoni, and Temeke) of the Dar es Salaam region of Tanzania, which contains the city of Dar es Salaam. The average household size in the Dar es Salaam region is 4.0 people and is virtually the same across its three municipalities (ranging from 3.9 to 4.0). Dar es Salaam’s HIV prevalence was 6.9% among adults aged 15–49 years in 2012, which is above the national prevalence of 5.1% [29].

#### The home-based carer program

This trial utilizes an existing and long-standing public-sector CHW cadre, called home-based carers or HBCs, to deliver the intervention. The CHWs are employed by Dar es Salaam’s municipalities and receive a stipend of 80,000 Tanzanian shilling (approximately equal to 36 US dollars) per month. They are lay healthcare workers whose main responsibility is to conduct regular home visits (at least once every three months) to all households in the neighborhood to which they have been assigned. The precise tasks of the CHWs during these home visits have varied somewhat over the years but generally consist of the provision of information and counseling on a wide variety of health topics, referral of ill clients to healthcare facilities, and promotion of preventive healthcare services. The CHW program exists in most but not all areas of Dar es Salaam. In those areas where the CHW program has been implemented, each neighborhood has one to three CHWs. The CHWs are residents in the neighborhoods in which they work.

### Study duration

**Fig. 1:**
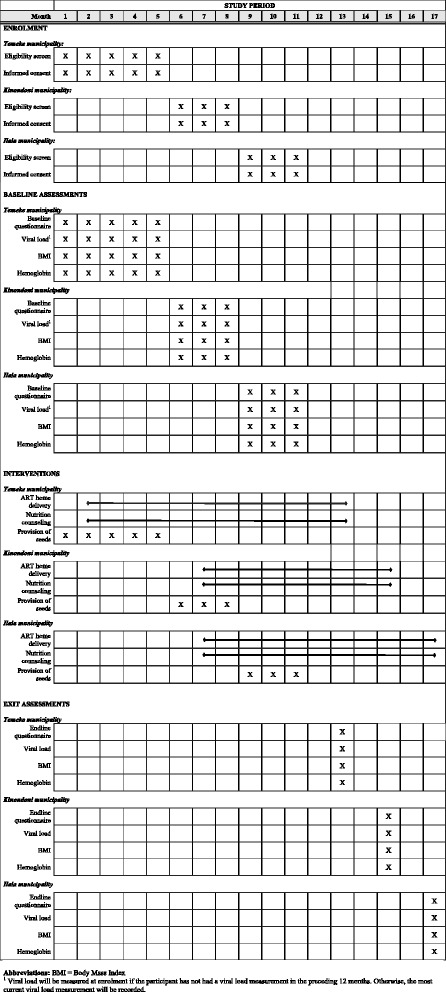
Trial activities over the study period

Enrolment into the trial took place in facilities in the Temeke municipality from February 29^th^ 2016 to July 29^th^ 2016. Because the number of participants enrolled in Temeke was lower than expected, the trial was expanded to 16 healthcare facilities in the Kinondoni municipality and 14 facilities in the Ilala municipality. Enrolment in Kinondoni took place from August 1^st^ 2016 to November 11^th^ 2016, and enrolment in Ilala from November 14^th^ 2016 to January 20^th^ 2017. The study activities during the trial period are detailed in Fig. 1.

### Eligibility criteria

The eligibility criteria for participants in this trial are: 1) being aged 18 years or older; 2) attending one of the participating healthcare facilities for ART during the enrolment period; and 3) living in a neighborhood in the facility’s catchment area. An additional eligibility criterion for ART home delivery is being clinically stable on ART. A patient is clinically stable on ART if the patient’s most current viral load has been taken less than 12 months prior to enrolment and showed viral suppression. If a viral load measurement is unavailable at the time of enrolment but a CD4-cell count taken in the 12 months prior to enrolment is available, then a patient is clinically stable on ART if the most current CD4-cell count is >350 cells/μL. If neither a viral load nor a CD4-count taken in the 12 months prior to enrolment is available, then a venous blood sample will be taken for a viral load measurement and the result used for the eligibility assessment. Additional requirements for being stable on ART are 1) taking ART for at least six months, and 2) having had a CD4-cell count >350 cells/μL or a suppressed viral load at six or more months after ART initiation. Patients who are pregnant at the time of enrolment (by patient self-report) or unable to provide written informed consent (e.g., due to mental incapacity) are excluded from eligibility.

### Study design

#### Rationale for a non-inferiority design and margin of non-inferiority

The non-inferiority design only applies to the primary endpoint (the proportion of participants with a suppressed HIV viral load). This design choice was made because in settings with an existing CHW program, such as in Dar es Salaam, CHW-led home delivery should likely be the standard of care if it does not negatively affect patients’ health outcomes as compared to standard facility-based care. Two virtually certain benefits of CHW-led ART home delivery are a reduction in 1) patient volume at healthcare facilities, which helps to alleviate the severe shortage of skilled healthcare workers in sub-Saharan Africa [20], and 2) the substantial time and financial (e.g., transport costs) burden on patients of having to attend an ART facility [24, 30]. The main drawback of CHW-led ART home delivery is the cost of establishing and running the CHW program, and the risk of overburdening CHWs, which may lead to a reduction in quality and/or quantity of care for non-ART patients. We would argue that in the case of Dar es Salaam, the cost consideration is minor as the CHW program already exists and is likely continue to run independently of whether CHWs are tasked with ART home delivery. Regarding overburdening CHWs, we carefully monitor the number of visits made by CHWs to non-HIV patients in each study arm and also ascertain CHWs’ job satisfaction and perception of the intervention through semi-structured qualitative interviews.

Based on our own judgement of what constitutes non-inferiority for this intervention and in line with the margin of equivalence of nine percentage points used by Jaffar et al. in their randomized trial of ART home delivery in rural Uganda [24], we chose a margin of non-inferiority of nine percentage points.

#### Randomization

**Fig. 2:**
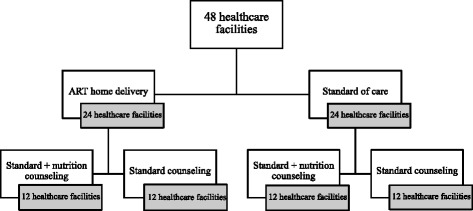
Randomization scheme

The unit of randomization is a healthcare facility with its surrounding catchment area (Fig. 2). Which healthcare facilities were included in this study was determined by the supervisory structure of CHWs in the routine public-sector health system. Each CHW is supervised by one community outreach nurse who is a nurse based at a healthcare facility. Each community outreach nurse supervises between three and 16 CHWs who work in neighborhoods (*mtaa* in Kiswahili) in the facility’s catchment area. We included all healthcare facilities in Dar es Salaam in this trial, which provide ART and had a community outreach nurse (and thus a team of affiliated CHWs) with the exception of two facilities (Amana Hospital and Mwananyamala District Hospital) because of a conflict with a clinical trial taking place at these facilities. Table 1 describes the characteristics of each cluster.

**Table 1:** Characteristics of the clusters

Name of healthcare facility	Counseling package^a^	Type of healthcare facility	Municipality	No. of patients currently on ART	No. of CHWs in cluster
ART home delivery					
1. Mbagala Rangi Tatu	Standard + nutrition	Hospital	Temeke	15,663	3
2. Tambukareli	Standard	Dispensary	Temeke	1,554	12
3. Yombo Makangarawe	Standard	Dispensary	Temeke	544	2
4. Toa Ngoma	Standard + nutrition	Dispensary	Temeke	239	12
5. Buza	Standard + nutrition	Dispensary	Temeke	215	5
6. Arafa Ugweno	Standard	Dispensary	Temeke	202	3
7. Mji mwema	Standard	Dispensary	Temeke	161	8
8. Kimbiji	Standard + nutrition	Dispensary	Temeke	119	6
9. Keko	Standard + nutrition	Dispensary	Temeke	79	4
10. Tandale	Standard	Dispensary	Kinondoni	2,951	9
11. Mburahati	Standard + nutrition	Dispensary	Kinondoni	1,639	11
12. Mwenge	Standard	Dispensary	Kinondoni	1,597	5
13. Mbezi	Standard + nutrition	Dispensary	Kinondoni	870	11
14. Hananasif	Standard + nutrition	Dispensary	Kinondoni	530	7
15. Kigogo	Standard	Dispensary	Kinondoni	347	3
16. Mabibo	Standard + nutrition	Dispensary	Kinondoni	278	9
17. Goba	Standard	Dispensary	Kinondoni	177	4
18. Tabata	Standard	Health Centre	Ilala	2,193	10
19. Vingunguti	Standard + nutrition	Health Centre	Ilala	1,865	7
20. Kitunda	Standard + nutrition	Health Centre	Ilala	768	10
21. Pugu Kajiungeni	Standard	Dispensary	Ilala	561	11
22. Tabata NBC	Standard + nutrition	Dispensary	Ilala	249	10
23. Kinyerezi	Standard	Dispensary	Ilala	238	13
24. Mongolandege	Standard	Dispensary	Ilala	152	16
		Total:		33,191^b^	191
Standard of care					
1. Temeke	Standard + nutrition	Hospital	Temeke	17,409	3
2. Kigamboni	Standard	Health Centre	Temeke	2,879	9
3. Mbagala Round Table	Standard + nutrition	Dispensary	Temeke	850	5
4. Maji Matitu	Standard	Dispensary	Temeke	540	7
5. Kichemchem	Standard + nutrition	Dispensary	Temeke	211	3
6. Kingugi	Standard	Dispensary	Temeke	166	6
7. Sandali	Standard	Dispensary	Temeke	148	9
8. Kibada	Standard + nutrition	Dispensary	Temeke	109	8
9. Kisarawe II	Standard	Dispensary	Temeke	63	5
10. Magomeni	Standard + nutrition	Health Center	Kinondoni	2,361	8
11. Kimara	Standard	Dispensary	Kinondoni	2,270	5
12. Bunju	Standard + nutrition	Dispensary	Kinondoni	1,595	7
13. Kawe	Standard	Dispensary	Kinondoni	844	5
14. Kijitonyama	Standard + nutrition	Dispensary	Kinondoni	750	8
15. Kinondoni	Standard	Hospital	Kinondoni	396	5
16. Makuburi	Standard	Dispensary	Kinondoni	256	4
17. Ununio	Standard + nutrition	Dispensary	Kinondoni	115	6
18. Mnazi Mmoja Health Centre	Standard + nutrition	Health Centre	Ilala	3,650	4
19. Chanika	Standard	Health Centre	Ilala	1,413	12
20. Segerea	Standard	Health Centre	Ilala	678	8
21. Kiwalani	Standard + nutrition	Dispensary	Ilala	519	13
22. Gerezani	Standard	Dispensary	Ilala	350	2
23. Majohe	Standard + nutrition	Dispensary	Ilala	220	10
24. Mvuti	Standard + nutrition	Dispensary	Ilala	185	16
		Total:		37,977^b^	168

*Abbreviations*: *ART* antiretroviral therapy, *No*. number, *CHW* community health worker

^a^ Half of the clusters in each study arm were randomized to standard counseling and half to standard plus nutrition counseling.

^b^ This is *not* the expected number of participants as many ART patients do not live in the cluster (i.e., in the area surrounding the healthcare facility), and are therefore not eligible for this trial

For the purposes of randomization, clusters were first matched into pairs, separately within each municipality, based on the number of patients currently on ART at the facility. More specifically, the facility with the highest number of ART patients in Temeke was paired with the facility with the second highest number of ART patients in Temeke, and so on. The reason for this choice was that the intervention becomes more complex and thus plausibly less feasible to implement with an increasing volume of eligible participants. Most facilities only have one community outreach nurse. Thus, with an increasing number of eligible participants (for which ART patient volume is a proxy), the number of patients for whom the community outreach nurse has to supervise ART home delivery increases. An added benefit of matching on size prior to randomization is that it ensured an approximately equal number of participants in each study arm. The same process of matching facilities into pairs based on ART patient volume was used to randomize half of the facilities in each study arm to standard counseling and half to standard plus nutrition counseling (Fig. 1). The study design is thus a matched-pair cluster-randomized controlled 2x2 factorial trial. The randomization was conducted prior to the start of the study using computer-generated random numbers. For feasibility reasons, neither the research team nor the study participants are blinded to the intervention assignment.

### Enrolment procedure

One to two study team members (henceforth referred to as data collectors) are placed full-time at each of the participating healthcare facilities for the duration of the enrolment period. The ART nurse at each of the participating healthcare facilities referes all ART patients who live in the facility’s catchment area to the data collector. The data collector introduces the study to the patient, and, provided the patient gives initial verbal consent, ascertains whether the patient is 1) stable on ART (see eligibility criteria), and 2) lives in the facility’s catchment area. If both criteria are fulfilled, the data collector conducts the written informed consent procedure, administers a tablet-based baseline questionnaire, and measures the participant’s height and weight. Next, the data collector asks the community outreach nurse at the facility to take a blood sample, which is sent for a measurement of blood hemoglobin. In addition, the blood sample is sent for an HIV viral load in participants who have not had a viral load measurement in the 12 months prior to study enrolment. Lastly, the data collector takes a map cue (a description of the location of the participant’s residence) from the participant and records his/her mobile phone number as well as the mobile phone number from at least one household member. These details are then passed to the CHW assigned to the neighborhood, in which the participant lives.

### Description of the interventions

#### Home delivery of ART

In clusters randomized to ART home delivery, a CHW visits participants at home to provide counseling (see below), deliver a supply of ART, and perform an ART pill count. After a year on ART, most patients in Dar es Salaam are scheduled to attend the facility every two months. We maintain a participant’s usual facility ART schedule in the ART home-delivery intervention. For instance, an ART patient who was scheduled to visit the facility every two months (and was provided with a two-months ART supply) receives a CHW visit for ART home delivery every two months (and receives a two-months ART supply from the CHW). The CHWs received three days of training in the home delivery of ART and in counseling skills for this intervention prior to the start of the trial.

#### Nutrition counseling

CHWs provide counseling to all participants in both the intervention and control arms during their home visits. As described in the study design section, half of the clusters in each the intervention and control arm of the trial were randomized to standard counseling, and half to standard plus nutrition counseling. The standard counseling covers family planning, ART adherence, and prevention of HIV transmission. Nutrition counseling covers advice on 1) food production for those with access to a garden or plot of land, 2) a healthy diet for people living with HIV, 3) exercise, and 4) monitoring one’s weight. In addition, participants enrolled in the trial at one of the healthcare facilities randomized to the nutrition counseling intervention receive a pack of seeds (amaranth, cowpea, or pumpkin) at enrolment if they report to have access to a garden or plot of land where they can grow vegetables for their own consumption.

### Endpoints

The primary endpoint for the ART home delivery intervention is the proportion of enrolled patients with a suppressed HIV viral load at the end of the study period (i.e., when the study exit assessment is performed). A substantial proportion of participants does not have a recent viral load or CD4-cell count at enrolment. As described above, the study team obtains a blood sample from these patients at enrolment and sends it to the public healthcare system’s laboratory for a viral load measurement. Because the delay in receiving the viral load count from the laboratory varies between participants (and thus, the time point at which eligibility for ART home delivery can be assessed at intervention facilities), the length of time for which intervention participants receive ART at home varies between participants at a healthcare facility. Participants in the ART home-delivery intervention receive ART at home for a period varying between one and 11 months, and for an average of six months.

The primary endpoint for the nutrition counseling intervention is the mean BMI of participants who received standard counseling by CHWs versus those who received standard plus nutrition counseling, assessed at the end of the study period. Secondary endpoints of this trial (all assessed at the end of the study period) are: 1) participants’ healthcare expenditures in the last six months, 2) self-reported ART adherence during the last one month, 3) the proportion of patients with access to a plot of land who grow vegetables or fruits for their own consumption, 4) diversity of dietary intake, and 5) the proportion of participants who are anemic.

### Sample size

Over the enrolment period, we expect to recruit approximately 700 participants in each of the two trial arms (1,400 participants in total). We calculated the design effect (taking into account clustering of outcomes at the facility-level and varying cluster sizes) for this trial using the ‘clustersampsi’ function in Stata [31]. The design effect was then used to adjust the expected power calculated for a non-inferiority trial under individual randomization, which we determined using the ‘ssi’ function in Stata [32]. Our calculations assumed that 85% of enrolled participants are virally suppressed at baseline as found in a recent cross-sectional study in Dar es Salaam [33]. The margin of non-inferiority was set at nine percentage points. We used a range of intra-cluster correlation coefficients (ICCs) from 0.005 to 0.020. Barnhart et al. calculated ICC values for CD4-cell count measures in Dar es Salaam [34]. The 6-months cumulative incidence for non-adherence to ART (defined as a 50% drop in CD4-count from its peak value and return to pre-ART CD4-count or lower after 168 days on ART or a viral load greater than 10,000 after 168 days on ART) had an ICC value of 0.016 (95% confidence interval: 0.009 to 0.029). We set the probability of a type one statistical error at 0.05 and assumed a correlation coefficient between baseline and endline viral load measurement of 0.5. We find that we are well powered (≥80% power) to detect modest one-sided differences (<9 percentage points) in the proportion of participants who are virally suppressed between the two study arms. Regarding the effect of nutrition counseling on BMI, we are well powered (≥80%) to detect a difference in mean BMI of approximately 1.0 kg/m^2^ or more between the participants in the standard versus the standard plus nutrition counseling arm. This difference is considerably smaller than the difference of 3.1 kg/m^2^ found by Alo et al. between female ART patients randomized to six months of nutrition counseling versus the standard of care in Southeast Nigeria [35]. The trial by Alo et al. is, to the best of our knowledge, the only randomized evaluation to date of the impact of nutrition counseling on BMI in ART patients.

### Data collection

This trial is being implemented by Management and Development for Health (MDH). MDH is a Tanzanian non-governmental organization based in Dar es Salaam, which works closely with Tanzania’s Ministry of Health and Social Welfare. MDH is working on this trial in partnership with the Harvard T.H. Chan School of Public Health, which provides technical assistance throughout the study period.

#### Biomarkers

HIV viral load and hemoglobin are measured at baseline and at the end of the study period. If a participant has had a viral load measurement in the 12 months prior to study enrolment, this measurement will be used as the baseline viral load.

#### Questionnaires

The study’s team of trained data collectors administers a tablet-based questionnaire at enrolment, and then again at the end of the study period. This questionnaire (see Additional file 1) covers self-reported ART adherence, health service utilization, out-of-pocket healthcare expenditures, satisfaction with CHW services, knowledge of HIV, utilization of family planning methods, dietary intake, physical activity, and food production. In addition, the data collectors administer a questionnaire during the enrolment period and during the follow-up period at each participating healthcare facility to a random set of patients after they have accessed an HIV service (i.e., HIV-testing, pre-ART, or ART). This questionnaire (see Additional file 2) asks about patient satisfaction with HIV-related healthcare services, time spent in the facility for the present visit, health service utilization and expenditure, and self-reported ART adherence. Patients for this questionnaire are selected using the sampling method of selecting the next patient entering the consultation [36]. Lastly, a healthcare provider questionnaire is administered to a random set of healthcare workers at each participating healthcare facility during the enrolment period and the follow-up period of the trial. This questionnaire (see Additional file 3) covers job satisfaction, perceived time pressure, and healthcare workers’ views on home delivery of ART.

#### Qualitative data collection

During the enrolment period and at the end of the study period, data collectors trained in qualitative interviewing will conduct semi-structured qualitative interviews with community outreach nurses, CHWs, and participants in the ART home-delivery arm. These interviews aim to ascertain healthcare workers’ and participants’ experiences with ART home delivery, and their suggestions for improvement in the delivery of the intervention. In addition, semi-structured qualitative interviews will be conducted with patients who were offered ART home delivery, but refused to enroll in ART home delivery, to identify their reasoning for preferring facility-based ART.

### Data monitoring

The ability for data monitoring in this trial is limited by the fact that no outcome data is collected on participants during the follow-up period. However, the follow-up period has intentionally been designed to be comparatively short so that in the case of ART home delivery resulting in considerably lower viral load suppression than facility-based care, the adverse effects on patients’ health are relatively minor. We do not expect adverse effects on patients’ health from this intervention given the encouraging results from the trials in Uganda [24] and Kenya [28]. Further, participants in the ART home delivery arm can opt to switch back to facility-based care at any time during the trial period. This trial therefore does not have a data safety and monitoring board.

### Analysis plan

We will analyze the data by intention-to-treat analysis at the patient level in all primary analyses for this trial. The primary endpoint of this trial (i.e., the percentage of participants with a suppressed HIV viral load at the end of the study period) will be assessed by fitting robust clustered log-binomial models. Given the relatively small number of clusters in this trial, we cannot assume that randomization will eliminate confounding despite the matching of clusters described in the study design section. We will, therefore, adjust the analysis for viral load suppression at baseline. As viral load suppression is the outcome variable for the primary endpoint, adjusting for baseline viral load could be considered perfect adjustment for confounders. However, in secondary analyses, we will also adjust for other potentially confounding variables (e.g., participants’ socio-demographic characteristics) as a single baseline measurement may not account for trends in viral load suppression over time. Similarly, in secondary analyses, we will adjust for differing average exposure lengths to ART home delivery at each facility, and conduct subgroup analyses according to whether a participant received ART home delivery and by participants’ exposure length to ART home delivery.

Potential effect modification for any of the endpoints of this trial will be assessed through the likelihood ratio test. If statistically significant effect modification is present, we will present relevant results stratified by the effect modifier. All analyses will be conducted using Stata (Release 13; StataCorp, College Station, TX, USA).

### Ethical considerations

#### Approvals

The study was approved by the research ethics committee of the National Institutes of Medical Research (NIMR) in Tanzania on July 16^th^ 2015 and received an exemption by the institutional review board of the Harvard T.H. Chan School of Public Health in June 2015. NIMR will be informed immediately of any study protocol amendments and the trial registers (RIDIE and ClinicalTrials.gov) will be updated accordingly.

#### Consent

The data collectors placed at each of the study’s healthcare facilities will obtain written informed consent from all participants at enrolment into the study. To adhere to the intent-to-treat principle, patients who refuse to participate in the study at any time during the study period, including during enrolment, will be asked for consent to complete the study questionnaire, and to take a blood sample and BMI measurement at enrolment and at the end of the study period. The data collectors will also obtain written informed consent from respondents before administering the patient exit questionnaire and the healthcare provider questionnaire.

#### Confidentiality

CHWs were instructed to seek a private space in the participant’s home before providing counseling and delivering ART. In addition, potential participants will be asked about whether they have disclosed their HIV status to other household members during enrolment. CHWs will be informed which participants have not disclosed their HIV status to all other household members and be urged to make an utmost effort to maintain the participant’s privacy during their home visits. Similarly, the patient exit and healthcare provider questionnaire will be administered in a space that maximizes the respondent’s privacy.

#### Access to data

The research team at MDH will have access to the full datasets collected as part of this study at all times during the trial. The MDH team will share de-identified datasets with the investigators at the Harvard T.H. Chan School of Public Health on a regular basis.

#### Dissemination of results

The trial results will be disseminated through personal meetings with policy makers in Dar es Salaam, including the municipal leadership and the Tanzanian Ministry of Health and Social Welfare. In addition, the results will be submitted for publication in a peer-reviewed scientific journal and presented at scientific conferences.

### Trial status

Enrolment into the trial has commenced on February 29^th^ 2016 and is currently ongoing.

## Discussion

Most low- and middle-income countries (LMICs) face a severe shortage of nurses and physicians [1], and distance to the nearest healthcare facility is a key barrier to accessing care in many regions [37–40]. Thus, the potential benefits of shifting care from skilled facility-based health worker cadres to lay community-based healthcare workers are evident. While this trial focuses on HIV care, it will contribute to a growing body of evidence on a broader question that is central to current health systems implementation research: what is the acceptability and feasibility of shifting care from facility-based nurses and physicians to lay healthcare workers based in the community, and what is the effect on patients’ health and economic outcomes? The question of whether drugs for chronic diseases can be safely provided by CHWs is one that is becoming increasingly important as the burden of chronic non-communicable diseases in LMICs grows [41]. The regular dispensing of drugs for chronic diseases may be a good candidate for task-shifting to CHWs for several reasons: 1) the frequency of needing to return to a healthcare facility to pick up medications may be determined by a facility’s drug stocks rather than a need for clinical monitoring by a nurse or physician, 2) there is a considerable cost to patients, both in terms of time lost and out-of-pocket expenditures [30], from having to regularly attend a healthcare facility, and 3) non-retention in care is common in many chronic care settings in LMICs [42].

### Limitations

This study has several limitations. First, there is the possibility of selection bias whereby ART patients in a control cluster may hear about the offer of ART home delivery at intervention facilities. These patients may change their care from a control to an intervention facility in order to receive ART at home. To assess whether this is a likely source of bias, we will ask patients at enrolment where and when they have last received ART and, where applicable, why they have changed healthcare facility. An additional threat to the internal validity of this trial could arise if a substantial portion of participants in the ART home-delivery arm does not return to the facility at the end of the follow-up period for a viral load measurement and administration of the endline questionnaire. If the patients who are lost to follow-up in the ART home-delivery arm have systematically different outcomes (e.g., are less likely to be virally suppressed) than patients who are not lost to follow-up, and this difference or the rate of loss to follow-up varies between the study arms, then this would result in bias. To minimize this potential bias, we will undertake additional efforts to collect viral load and BMI data from participants in the ART home-delivery arm who do not return to the healthcare facility at the end of the follow-up period, including taking blood samples for viral load measurement at participants’ homes. A limitation to the external validity of this trial is that it focuses on home delivery of ART for patients who are stable on ART. home delivery of ART may, however, also be feasible for patients who just initiated ART, or whose WHO clinical stage, CD4-cell count or viral load suggests that they are not stable ART patients. For example, in a sub-analysis in patients who started ART with a low CD4-cell count (<50 cells/μL) as part of the cluster-randomized trial in Uganda described in the introduction, Woodd et al. did not find an increased rate of mortality among those who continued to receive ART at home after ART initiation as compared to those who received standard clinic-based care [43].

In conclusion, this is the first randomized health systems implementation trial investigating the feasibility and effectiveness of CHW-led ART home delivery in urban sub-Saharan Africa. The results of this trial will not only inform ART programs in sub-Saharan Africa but also contribute an important piece of evidence to the ongoing policy debate on task-shifting of chronic care from facility- to community-based healthcare workers.

## Additional files

Additional file 1: Baseline Patient Questionnaire. (DOCX 314 kb)
Additional file 2: Patient Exit Questionnaire. (DOCX 325 kb)
Additional file 3: Healthcare Provider Questionnaire. (DOCX 142 kb)

## Abbreviations

ART: Antiretroviral therapy
BMI: Body mass index
CD4: Cluster of differentiation 4
CHW: Community health worker
HBC: Home-based carer
HIV: Human immunodeficiency virus
LMICs: Low-and middle-income countries
MDH: Management and Development for Health
PMTCT: Prevention of mother-to-child HIV transmission
RR: Relative risk
SSA: Sub-Saharan Africa
WHO: World Health Organization
μL: Microliter
