# Supplementary material for: The impact of community health worker-led home delivery of antiretroviral therapy on virological suppression: a non-inferiority cluster-randomized health systems trial in Dar es Salaam, Tanzania
Source: BMC Health Serv Res. 2017 Feb 22;17:160. doi: 10.1186/s12913-017-2032-7 (PMC5322683; doi:10.1186/s12913-017-2032-7)
Supplement: Additional file 1: — Baseline Patient Questionnaire. (DOCX 314 kb) [file 12913_2017_2032_MOESM1_ESM.docx]

#### Baseline Patient Questionnaire

| Field | Question | Answer |
| --- | --- | --- |
| introgroup | | |
| \|  \| cover \| \| --- \| --- \| | Welcome to the Baseline Patient Questionnaire.  0.1: INTERVIEWER INFORMATION |  |
| \|  \| interviewer_id (required) \| \| --- \| --- \| | Select interviewer name: | \|  \| 1 \| Grace Joseph Matemu \| \| --- \| --- \| --- \| \|  \| 2 \| Geofrey Isdory \| \|  \| 3 \| Glory William \| \|  \| 4 \| Tunkine Sanga \| \|  \| 5 \| Willy Ulkaye \| \|  \| 6 \| Lilian Lwanda \| \|  \| 7 \| Sakina Hamisi \| \|  \| 8 \| Happiness Madadi \| \|  \| 9 \| Mgalama Jaqueline \| \|  \| 10 \| Jeila Maulid \| \|  \| 11 \| Aidath Murusuri \| \|  \| 12 \| Judith Mmari \| \|  \| 13 \| Joan Reno Mori \| \|  \| 14 \| Paul Msale \| \|  \| 15 \| Charles Kafula \| \|  \| 16 \| Josephine Uiso \| \|  \| 17 \| Irene Machume \| \|  \| 18 \| Flora Musa \| \|  \| 97 \| OTHER \| |
| other_interviewer | | |
| \|  \| other_interviewer_note \| \| --- \| --- \| | You selected 'OTHER' interviewer. Please enter your first and last name. |  |
| \|  \| other_interviewer_first (required) \| \| --- \| --- \| | First name of interviewer: |  |
| \|  \| other_interviewer_last (required) \| \| --- \| --- \| | Last name of interviewer: |  |
| facility (required) | Name of healthcare facility: | \|  \| 1 \| MAJIMATITU \| \| --- \| --- \| --- \| \|  \| 2 \| KINGUGI \| \|  \| 3 \| KIMBIJI DISP \| \|  \| 4 \| KISARAWA 11 \| \|  \| 5 \| KIGAMBONI HOSP \| \|  \| 6 \| MJUMWEMA DISP \| \|  \| 7 \| T/RELI DISP \| \|  \| 8 \| TEMEKE HOSP \| \|  \| 9 \| BUZA DISP \| \|  \| 10 \| ARAFA UGWENO \| \|  \| 11 \| KIBADA DISP \| \|  \| 12 \| SANDALI DESP \| \|  \| 13 \| MBAGALA R/TATU \| \|  \| 14 \| KICHEMCHEM DISP \| \|  \| 15 \| KEKO DISP \| \|  \| 16 \| MAKANGARAWE \| \|  \| 17 \| TOANGOMA \| \|  \| 18 \| MBAGALA ROUND TABLE \| \|  \| 19 \| Magomeni Health Center \| \|  \| 20 \| Kimara Dispensary \| \|  \| 21 \| Bunju Dispensary \| \|  \| 22 \| Kawe Dispensary \| \|  \| 23 \| Kijitonyama Dispensary \| \|  \| 24 \| Kinondoni Hospital - Other Hospital \| \|  \| 25 \| Makuburi Dispensary \| \|  \| 27 \| Ununio Dispensary \| \|  \| 28 \| Tandale Dispensary \| \|  \| 29 \| Mburahati Dispensary \| \|  \| 30 \| Mwenge Dispensary \| \|  \| 31 \| Mbezi Dispensary \| \|  \| 32 \| Hananasif Dispensary \| \|  \| 33 \| Kigogo Dispensary \| \|  \| 34 \| Mabibo Dispensary \| \|  \| 35 \| Goba Dispensary \| |
| ward_temeke (required) | Is the ward of residence in Temeke or Kinondoni? | \|  \| 1 \| Temeke \| \| --- \| --- \| --- \| \|  \| 2 \| Kinondoni \| |
| ward_in_temeke (required) | Select ward of residence in Temeke: | \|  \| 1 \| Azimio \| \| --- \| --- \| --- \| \|  \| 2 \| Buza \| \|  \| 3 \| Chamazi \| \|  \| 4 \| Chang'ombe \| \|  \| 5 \| Charambe \| \|  \| 6 \| Keko \| \|  \| 7 \| Kigamboni \| \|  \| 8 \| Kibada \| \|  \| 9 \| Kimbiji \| \|  \| 10 \| Kisarawe II \| \|  \| 11 \| Kurasini \| \|  \| 12 \| Makangarawe \| \|  \| 13 \| Mbagala \| \|  \| 14 \| Mbagala Kuu \| \|  \| 15 \| Miburani \| \|  \| 16 \| Mjimwema \| \|  \| 17 \| Mtoni \| \|  \| 18 \| Pemba Mnazi \| \|  \| 19 \| Sandali \| \|  \| 20 \| Somangila \| \|  \| 21 \| Tandika \| \|  \| 22 \| Temeke \| \|  \| 23 \| Toangoma \| \|  \| 24 \| Vijibweni \| \|  \| 25 \| Yombo Vituka \| \|  \| 26 \| Tungi \| \|  \| 27 \| Kiburugwa \| \|  \| 28 \| Kilungule \| \|  \| 29 \| Kilakila \| \|  \| 30 \| Mianzini \| \|  \| 31 \| Kijichi \| \|  \| 32 \| Kibondemaji \| \|  \| 97 \| OTHER \| |
| ward_in_kinondoni (required) | Select ward of residence in Kinondoni: | \|  \| 1 \| Bunju \| \| --- \| --- \| --- \| \|  \| 2 \| Goba \| \|  \| 3 \| Hananasifu \| \|  \| 4 \| Kawe \| \|  \| 5 \| Kibamba \| \|  \| 6 \| Kigogo \| \|  \| 7 \| Kijitonyama \| \|  \| 8 \| Kimara \| \|  \| 9 \| Kinondoni \| \|  \| 10 \| Kunduchi \| \|  \| 11 \| Kwembe \| \|  \| 12 \| Mabibo \| \|  \| 13 \| Mabwe Pande \| \|  \| 14 \| Magomeni \| \|  \| 15 \| Makongo \| \|  \| 16 \| Makuburi \| \|  \| 17 \| Makumbusho \| \|  \| 18 \| Makurumla \| \|  \| 19 \| Manzese \| \|  \| 20 \| Mbezi \| \|  \| 21 \| Mbezi Juu \| \|  \| 22 \| Mburahati \| \|  \| 23 \| Mikocheni \| \|  \| 24 \| Msasani \| \|  \| 25 \| Msigani \| \|  \| 26 \| Mwananyamala \| \|  \| 27 \| Mzimuni \| \|  \| 28 \| Ndugumbi \| \|  \| 29 \| Saranga \| \|  \| 30 \| Sinza \| \|  \| 31 \| Tandale \| \|  \| 32 \| Ubungo \| \|  \| 33 \| Wazo \| \|  \| 97 \| OTHER \| |
| other_ward_temeke (required) | Please specify 'Other' ward: |  |
| other_ward_kinondoni (required) | Please specify 'Other' ward: |  |
| facility_mtaa (required) | Select mtaa of residence: | \|  \| 1 \| Kimbangulile \| \| --- \| --- \| --- \| \|  \| 2 \| Kurasini Mjimpya \| \|  \| 3 \| Nzasa \| \|  \| 4 \| Rangi Tatu \| \|  \| 5 \| Nzasa 'B' \| \|  \| 6 \| Mianzini \| \|  \| 7 \| Majimatitu \| \|  \| 8 \| Rangi Tatu \| \|  \| 9 \| Mchikichini \| \|  \| 10 \| Majimatitu B' \| \|  \| 11 \| Majimatitu \| \|  \| 197 \| None of the mtaas above \| \|  \| 12 \| Kwa Nyoka \| \|  \| 13 \| Kiburugwa Origin \| \|  \| 14 \| Kiburugwa namna Tatu \| \|  \| 15 \| Kingugi \| \|  \| 297 \| None of the mtaas above \| \|  \| 16 \| Mikenge \| \|  \| 17 \| Kizito \| \|  \| 18 \| Ngobanya \| \|  \| 19 \| Kwa chale \| \|  \| 397 \| None of the mtaas above \| \|  \| 20 \| Vumilia Ukooni \| \|  \| 21 \| Mwasonga \| \|  \| 497 \| None of the mtaas above \| \|  \| 22 \| Kigamboni \| \|  \| 23 \| Ferry \| \|  \| 24 \| Tuamoyo \| \|  \| 597 \| None of the mtaas above \| \|  \| 25 \| Mjimwema \| \|  \| 26 \| Maweni \| \|  \| 27 \| Ungindoni \| \|  \| 28 \| Kibugumo \| \|  \| 697 \| None of the mtaas above \| \|  \| 29 \| Kichangani \| \|  \| 30 \| Tambukareli \| \|  \| 31 \| Mjimpya \| \|  \| 32 \| Mbuyuni \| \|  \| 33 \| Azimio \| \|  \| 34 \| Azimio Kurasini \| \|  \| 35 \| Mtongani \| \|  \| 797 \| None of the mtaas above \| \|  \| 36 \| Keko Juu \| \|  \| 37 \| Wailes \| \|  \| 38 \| Mibulani \| \|  \| 897 \| None of the mtaas above \| \|  \| 39 \| Buza \| \|  \| 40 \| Machine ya Maji \| \|  \| 41 \| Mjimpya \| \|  \| 997 \| None of the mtaas above \| \|  \| 42 \| Tamla \| \|  \| 43 \| Kilimahewa \| \|  \| 44 \| Mabatini \| \|  \| 1097 \| None of the mtaas above \| \|  \| 45 \| Kifurukwe \| \|  \| 46 \| Uvumba \| \|  \| 47 \| Nyakwale \| \|  \| 48 \| Kiziza \| \|  \| 49 \| Kichangani \| \|  \| 50 \| Sokoni \| \|  \| 1197 \| None of the mtaas above \| \|  \| 51 \| Mkwinda \| \|  \| 52 \| Mnofu \| \|  \| 53 \| Mamboleo \| \|  \| 54 \| Tindwa \| \|  \| 55 \| Mpogo \| \|  \| 56 \| Vetenary \| \|  \| 57 \| Mwembeladu \| \|  \| 58 \| Kimbunga \| \|  \| 59 \| Usalama \| \|  \| 1297 \| None of the mtaas above \| \|  \| 60 \| Mbagala Kuu \| \|  \| 61 \| Mbagala Kuu Kaskazini \| \|  \| 62 \| Mbagala Kuu Mashariki \| \|  \| 63 \| Jeshi La Wokovu \| \|  \| 1397 \| None of the mtaas above \| \|  \| 64 \| Kichemichemi \| \|  \| 65 \| Kizuiani \| \|  \| 66 \| Makuka \| \|  \| 1497 \| None of the mtaas above \| \|  \| 67 \| Magurumbasi A \| \|  \| 68 \| Keko Mwanga B' \| \|  \| 69 \| Magurumbasi B \| \|  \| 1597 \| None of the mtaas above \| \|  \| 70 \| Yombo dovya \| \|  \| 71 \| Makangarawe \| \|  \| 1697 \| None of the mtaas above \| \|  \| 72 \| Kongowe \| \|  \| 73 \| Masaki \| \|  \| 74 \| Mponde \| \|  \| 75 \| Goroka \| \|  \| 76 \| Toangoma \| \|  \| 77 \| Mikwambe \| \|  \| 78 \| Masuliza \| \|  \| 79 \| Ponde \| \|  \| 80 \| Mzinga \| \|  \| 1797 \| None of the mtaas above \| \|  \| 81 \| Kizinga \| \|  \| 82 \| Bughdadi \| \|  \| 83 \| Mangaya \| \|  \| 84 \| Moringe \| \|  \| 85 \| Mbagala \| \|  \| 1897 \| None of the mtaas above \| \|  \| 86 \| Tungi \| \|  \| 87 \| Muungano \| \|  \| 88 \| Magogoni \| \|  \| 89 \| Kwa Mwinyi \| \|  \| 90 \| Juhudi \| |
| other_facility_mtaa (required) | Please specify 'Other' mtaa: |  |
| reachable (required) | Does the patient live in a ward that is reachable by the HBC? | \|  \| 1 \| Yes \| \| --- \| --- \| --- \| \|  \| 2 \| No \| |
| not_reachable | Thank you for your time. Have a nice day.  The participant is not in a reachable ward. Swipe forward to save and exit this form. |  |
| reachable_group | | |
| \|  \| interview_date (required) \| \| --- \| --- \| | Confirm today's date: |  |
| \|  \| reachable_group > participant_name \| \| --- \| --- \| | | |
| \|  \|  \| note_name \| \| --- \| --- \| --- \| | Names of participant: |  |
| \|  \|  \| first_name (required) \| \| --- \| --- \| --- \| | First name |  |
| \|  \|  \| second_name \| \| --- \| --- \| --- \| | Second name |  |
| \|  \|  \| last_name (required) \| \| --- \| --- \| --- \| | Last name |  |
| \|  \| rsp_id (required) \| \| --- \| --- \| | Counting all of the people you have interviewed today, what number survey is this? |  |
| \|  \| rsp_id_note \| \| --- \| --- \| | The respondent's study ID number is [rspid_final] |  |
| \|  \|  \| ctc2 (required) \| \| --- \| --- \| --- \| | What is the CTC2 number of the respondent?  Enter 14 digits and 1 dash. Example: 12345678-123456 |  |
| \|  \|  \| time_start \| \| --- \| --- \| --- \| | Time at start of interview |  |
| \|  \|  \| resp_mobile (required) \| \| --- \| --- \| --- \| | Mobile phone number of the respondent  If participant does not have their own mobile phone, enter '0000' |  |
| \|  \|  \| contact_note \| \| --- \| --- \| --- \| | ASK RESPONDENT FOR THE NAMES, RELATIONSHIPS, AND MOBILE NUMBERS OF HIS/HER HOUSEHOLD CONTACTS. |  |
| \|  \|  \| reachable_group > consented_group > hh_contact1 \| \| --- \| --- \| --- \| | | |
| \|  \|  \|  \| hh_contact1a (required) \| \| --- \| --- \| --- \| --- \| | Name of Household Contact 1 |  |
| \|  \|  \|  \| hh_contact1b (required) \| \| --- \| --- \| --- \| --- \| | Relationship of Household Contact 1 | \|  \| 1 \| Wife or husband \| \| --- \| --- \| --- \| \|  \| 2 \| Son or daughter \| \|  \| 3 \| Son-in-law or daughter-in-law \| \|  \| 4 \| Grandchild \| \|  \| 5 \| Parent \| \|  \| 6 \| Parent-in-law \| \|  \| 7 \| Brother or sister \| \|  \| 8 \| Other relative \| \|  \| 9 \| Adopted / foster / stephchild \| \|  \| 10 \| Not related \| \|  \| 98 \| DON'T KNOW \| |
| \|  \|  \|  \| hh_contact1c (required) \| \| --- \| --- \| --- \| --- \| | Phone number of Household Contact 1 |  |
| \|  \|  \| reachable_group > consented_group > hh_contact2 \| \| --- \| --- \| --- \| | | |
| \|  \|  \|  \| hh_contact2a \| \| --- \| --- \| --- \| --- \| | Name of Household Contact 2 |  |
| \|  \|  \|  \| hh_contact2b \| \| --- \| --- \| --- \| --- \| | Relationship of Household Contact 2 | \|  \| 1 \| Wife or husband \| \| --- \| --- \| --- \| \|  \| 2 \| Son or daughter \| \|  \| 3 \| Son-in-law or daughter-in-law \| \|  \| 4 \| Grandchild \| \|  \| 5 \| Parent \| \|  \| 6 \| Parent-in-law \| \|  \| 7 \| Brother or sister \| \|  \| 8 \| Other relative \| \|  \| 9 \| Adopted / foster / stephchild \| \|  \| 10 \| Not related \| \|  \| 98 \| DON'T KNOW \| |
| \|  \|  \|  \| hh_contact2c \| \| --- \| --- \| --- \| --- \| | Phone number of Household Contact 2 |  |
| \|  \|  \| reachable_group > consented_group > hh_contact3 \| \| --- \| --- \| --- \| | | |
| \|  \|  \|  \| hh_contact3a \| \| --- \| --- \| --- \| --- \| | Name of Household Contact 3 |  |
| \|  \|  \|  \| hh_contact3b \| \| --- \| --- \| --- \| --- \| | Relationship of Household Contact 3 | \|  \| 1 \| Wife or husband \| \| --- \| --- \| --- \| \|  \| 2 \| Son or daughter \| \|  \| 3 \| Son-in-law or daughter-in-law \| \|  \| 4 \| Grandchild \| \|  \| 5 \| Parent \| \|  \| 6 \| Parent-in-law \| \|  \| 7 \| Brother or sister \| \|  \| 8 \| Other relative \| \|  \| 9 \| Adopted / foster / stephchild \| \|  \| 10 \| Not related \| \|  \| 98 \| DON'T KNOW \| |
| \|  \|  \|  \| hh_contact3c \| \| --- \| --- \| --- \| --- \| | Phone number of Household Contact 3 |  |
| \|  \|  \| mobile_consent (required) \| \| --- \| --- \| --- \| | Would it be alright for a study team member to call any of these numbers in case they cannot find you in the community? | \|  \| 1 \| Yes \| \| --- \| --- \| --- \| \|  \| 2 \| No \| |
| \|  \|  \| note_a \| \| --- \| --- \| --- \| | PART 1: BASIC IDENTIFYING & SOCIODEMOGRAPHIC INFORMATION |  |
| \|  \|  \| a1 (required) \| \| --- \| --- \| --- \| | WHAT IS THE SEX OF THE RESPONDENT?  Only ask if not obvious. | \|  \| 1 \| Female \| \| --- \| --- \| --- \| \|  \| 2 \| Male \| |
| \|  \|  \| a2 (required) \| \| --- \| --- \| --- \| | How old are you?  Please enter the age in years. If respondent does not know, but confirms that he/she is over 18, enter 1111. |  |
| \|  \| below18 \| \| --- \| --- \| | Thank you for your time. Have a nice day.  You cannot continue because the participant is under 18. Save and exit this form. |  |
| \|  \| reachable_group > over18_group \| \| --- \| --- \| | | |
| \|  \|  \| reachable_group > over18_group > a3_group \| \| --- \| --- \| --- \| | | |
| \|  \|  \|  \| a3 (required) \| \| --- \| --- \| --- \| --- \| | Are you married? | \|  \| 1 \| Yes (incl. civil and custom marriage) \| \| --- \| --- \| --- \| \|  \| 2 \| No, but I live with my partner \| \|  \| 3 \| No, and I live alone or with other family members \| \|  \| 4 \| Divorced/separated from husband/wife \| \|  \| 5 \| Widowed \| \|  \| 99 \| REFUSED \| \|  \| 6 \| Other (specify below) \| |
| \|  \|  \|  \| a3_other (required) \| \| --- \| --- \| --- \| --- \| | Please specify 'Other': |  |
| \|  \|  \| a4_pre \| \| --- \| --- \| --- \| | IS THE RESPONDENT HIV-POSITIVE? | \|  \| 1 \| Yes \| \| --- \| --- \| --- \| \|  \| 2 \| No \| |
| \|  \|  \| not_hiv_positive \| \| --- \| --- \| --- \| | Thank you for your time. Have a nice day.  You cannot continue because the participant is not HIV-positive. Swipe forward to save and exit this form. |  |
| \|  \|  \| reachable_group > over18_group > hiv_positive \| \| --- \| --- \| --- \| | | |
| \|  \|  \|  \| a4 (required) \| \| --- \| --- \| --- \| --- \| | When did you find out that you are HIV-positive?  If respondent is not HIV-positive, please save the form and exit. If respondent DK, enter February 1980. If respondent RF, enter March 1980. |  |
| \|  \|  \|  \| reachable_group > over18_group > hiv_positive > a5_group \| \| --- \| --- \| --- \| --- \| | | |
| \|  \|  \|  \|  \| a5 \| \| --- \| --- \| --- \| --- \| --- \| | For how many months or years have you been attending this clinic to get your ART? |  |
| \|  \|  \|  \|  \| a5_months \| \| --- \| --- \| --- \| --- \| --- \| | Months: |  |
| \|  \|  \|  \|  \| a5_years (required) \| \| --- \| --- \| --- \| --- \| --- \| | Years: |  |
| \|  \|  \|  \| reachable_group > over18_group > hiv_positive > less_than_year_ART \| \| --- \| --- \| --- \| --- \| | | |
| \|  \|  \|  \|  \| a6 (required) \| \| --- \| --- \| --- \| --- \| --- \| | Which clinic were you attending for your ART before? | \|  \| 1 \| MAJIMATITU \| \| --- \| --- \| --- \| \|  \| 2 \| KINGUGI \| \|  \| 3 \| KIMBIJI DESP \| \|  \| 4 \| KISARAWA 11 \| \|  \| 5 \| KIGAMBONI HOSP \| \|  \| 6 \| MJUMWEMA DISP \| \|  \| 7 \| T/RELI DISP \| \|  \| 8 \| TEMEKE HOSP \| \|  \| 9 \| BUZA DISP \| \|  \| 10 \| ARAFA UGWENO \| \|  \| 11 \| KIBADA DISP \| \|  \| 12 \| SANDALI DESP \| \|  \| 13 \| MBAGALA R/TATU \| \|  \| 14 \| KICHEMCHEM DISP \| \|  \| 15 \| KEKO DISP \| \|  \| 16 \| MAKANGARAWE \| \|  \| 17 \| TOANGOMA \| \|  \| 18 \| MBAGALA ROUND TABLE \| \|  \| 19 \| Magomeni Health Center \| \|  \| 20 \| Kimara Dispensary \| \|  \| 21 \| Bunju Dispensary \| \|  \| 22 \| Kawe Dispensary \| \|  \| 23 \| Kijitonyama Dispensary \| \|  \| 24 \| Kinondoni Hospital - Other Hospital \| \|  \| 25 \| Makuburi Dispensary \| \|  \| 27 \| Ununio Dispensary \| \|  \| 28 \| Tandale Dispensary \| \|  \| 29 \| Mburahati Dispensary \| \|  \| 30 \| Mwenge Dispensary \| \|  \| 31 \| Mbezi Dispensary \| \|  \| 32 \| Hananasif Dispensary \| \|  \| 33 \| Kigogo Dispensary \| \|  \| 34 \| Mabibo Dispensary \| \|  \| 35 \| Goba Dispensary \| \|  \| 96 \| None \| \|  \| 97 \| Other \| \|  \| 98 \| Don't know \| \|  \| 99 \| Refused \| |
| \|  \|  \|  \|  \| a6_other (required) \| \| --- \| --- \| --- \| --- \| --- \| | Please specify 'Other' clinic you attended for ART before. |  |
| \|  \|  \|  \|  \| reachable_group > over18_group > hiv_positive > less_than_year_ART > a7_group \| \| --- \| --- \| --- \| --- \| --- \| | | |
| \|  \|  \|  \|  \|  \| a7 \| \| --- \| --- \| --- \| --- \| --- \| --- \| | For how many months or years had you been attending that clinic to get your ART? |  |
| \|  \|  \|  \|  \|  \| a7_months \| \| --- \| --- \| --- \| --- \| --- \| --- \| | Months: |  |
| \|  \|  \|  \|  \|  \| a7_years (required) \| \| --- \| --- \| --- \| --- \| --- \| --- \| | Years: |  |
| \|  \|  \|  \| reachable_group > over18_group > hiv_positive > education_highest \| \| --- \| --- \| --- \| --- \| | | |
| \|  \|  \|  \|  \| a8 (required) \| \| --- \| --- \| --- \| --- \| --- \| | What is the highest level of school you attended: preschool, primary school, secondary school, or high school? | \|  \| 1 \| None \| \| --- \| --- \| --- \| \|  \| 2 \| Preschool \| \|  \| 3 \| Primary school \| \|  \| 4 \| Secondary school \| \|  \| 5 \| High school \| \|  \| 99 \| REFUSED \| \|  \| 97 \| Other (specify) \| |
| \|  \|  \|  \| a8_other (required) \| \| --- \| --- \| --- \| --- \| | Please specify 'Other' education level: |  |
| \|  \|  \|  \| a9 (required) \| \| --- \| --- \| --- \| --- \| | Have you completed any education after school? | \|  \| 1 \| Yes \| \| --- \| --- \| --- \| \|  \| 2 \| No \| \|  \| 99 \| REFUSED \| |
| \|  \|  \|  \| reachable_group > over18_group > hiv_positive > highest_after \| \| --- \| --- \| --- \| --- \| | | |
| \|  \|  \|  \|  \| a10 (required) \| \| --- \| --- \| --- \| --- \| --- \| | What is the highest level of education you completed after school? | \|  \| 1 \| Teacher-training College \| \| --- \| --- \| --- \| \|  \| 2 \| Nursing Assistant College \| \|  \| 3 \| Nursing College \| \|  \| 4 \| Other technical or vocational college \| \|  \| 5 \| University (studying towards Bachelor) \| \|  \| 6 \| University (studying towards Master) \| \|  \| 7 \| University (studying medicine or law) \| \|  \| 8 \| University (studying towards PhD) \| \|  \| 99 \| REFUSED \| \|  \| 97 \| Other (specify) \| |
| \|  \|  \|  \| a10_other (required) \| \| --- \| --- \| --- \| --- \| | Please specify 'Other' education level: |  |
| \|  \|  \|  \| a11 (required) \| \| --- \| --- \| --- \| --- \| | Have you done any work in the last six months? With work, we mean any activity to earn money or obtain food. | \|  \| 1 \| Yes \| \| --- \| --- \| --- \| \|  \| 2 \| No \| \|  \| 99 \| REFUSED \| |
| \|  \|  \|  \| a12 (required) \| \| --- \| --- \| --- \| --- \| | What is your occupation? That is, what kind of work do you mainly do? | \|  \| 1 \| Farm work \| \| --- \| --- \| --- \| \|  \| 2 \| Domestic work \| \|  \| 3 \| Construction work \| \|  \| 4 \| Security work \| \|  \| 5 \| Cleaning work \| \|  \| 6 \| Small business owner \| \|  \| 7 \| Mine work \| \|  \| 8 \| Teacher \| \|  \| 9 \| Traditional healer \| \|  \| 10 \| Nurse or Nurse assistant \| \|  \| 11 \| Physician or Surgeon \| \|  \| 12 \| Other healthcare worker \| \|  \| 13 \| Game farm / game reserve (eg ranger) \| \|  \| 14 \| Driver \| \|  \| 15 \| Skiller worker (eg plumber, mechanic, electrician) \| \|  \| 16 \| Cook / chef / catering \| \|  \| 17 \| Unskilled worker (eg general labourer) \| \|  \| 18 \| Artisan (eg carpenter, woodcarver, weaver) \| \|  \| 19 \| Waiter / barman \| \|  \| 20 \| Informal selling \| \|  \| 21 \| Small business assistant \| \|  \| 22 \| Clerical and office work \| \|  \| 23 \| Cattle herder \| \|  \| 24 \| Sewing, hairdressing, baking, brewing \| \|  \| 25 \| Police, soldier, fireman \| \|  \| 26 \| Petrol attendant \| \|  \| 27 \| Timber, sawmill, poles \| \|  \| 28 \| Gardening services \| \|  \| 29 \| Fieldworker - NGO or university \| \|  \| 30 \| Art, craft, photography, fashion design \| \|  \| 31 \| Senior administrator, manager, professional \| \|  \| 32 \| Priest / pastor \| \|  \| 33 \| Other \| \|  \| 98 \| DON'T KNOW \| \|  \| RF \| REFUSED \| |
| \|  \|  \|  \| a12_other (required) \| \| --- \| --- \| --- \| --- \| | YOU SELECTED 'Other healthcare worker' OR 'Other'. PLEASE SPECIFY: |  |
| \|  \|  \|  \| reachable_group > over18_group > hiv_positive > not_working \| \| --- \| --- \| --- \| --- \| | | |
| \|  \|  \|  \|  \| a13 (required) \| \| --- \| --- \| --- \| --- \| --- \| | Why have you not worked in the last 12 months? | \|  \| 1 \| Was in full-time education \| \| --- \| --- \| --- \| \|  \| 2 \| Unable to work (disabled) \| \|  \| 3 \| Unemployed \| \|  \| 4 \| Homemaker \| \|  \| 5 \| Looked after my (grand) children \| \|  \| 6 \| Could not work because of pregnancy \| \|  \| 7 \| Retired \| \|  \| 8 \| Sick leave \| \|  \| 9 \| Other leave \| \|  \| 97 \| Other (specify) \| \|  \| 99 \| REFUSED \| |
| \|  \|  \|  \| a13_other \| \| --- \| --- \| --- \| --- \| | Please specify 'Other' reason for not working in the last 12 months: |  |
| \|  \|  \|  \| reachable_group > over18_group > hiv_positive > water \| \| --- \| --- \| --- \| --- \| | | |
| \|  \|  \|  \|  \| a14 (required) \| \| --- \| --- \| --- \| --- \| --- \| | What is the main source of drinking water for members of your household? | \|  \| 1 \| Piped into dwelling \| \| --- \| --- \| --- \| \|  \| 2 \| Piped to yard/plot \| \|  \| 3 \| Public taps/standpipe \| \|  \| 4 \| Borehold \| \|  \| 5 \| Protected well \| \|  \| 6 \| Unprotected well \| \|  \| 7 \| Protected spring \| \|  \| 8 \| Unprotected spring \| \|  \| 9 \| Rainwater \| \|  \| 10 \| Tanker truck \| \|  \| 11 \| Surface water (river/dam/lake/pond/stream/canal/irrigation channel) \| \|  \| 12 \| Bottled water \| \|  \| 99 \| REFUSED \| \|  \| 13 \| Other (specify below) \| |
| \|  \|  \|  \|  \| a14_other \| \| --- \| --- \| --- \| --- \| --- \| | Please specify 'Other' main drinking water source: |  |
| \|  \|  \|  \| a15 (required) \| \| --- \| --- \| --- \| --- \| | Do you do anything to the water to make it safer to drink? | \|  \| 1 \| Yes \| \| --- \| --- \| --- \| \|  \| 2 \| No \| \|  \| 99 \| REFUSED \| |
| \|  \|  \|  \| reachable_group > over18_group > hiv_positive > toilet \| \| --- \| --- \| --- \| --- \| | | |
| \|  \|  \|  \|  \| a16 (required) \| \| --- \| --- \| --- \| --- \| --- \| | What kind of toilet facility do members of your household usually use? | \|  \| 1 \| Flush or pour flush toilet \| \| --- \| --- \| --- \| \|  \| 2 \| Ordinary pit toilet \| \|  \| 3 \| Ventilated improved privy (VIP) \| \|  \| 4 \| No facility/bush/field \| \|  \| 99 \| REFUSED \| \|  \| 5 \| Other (specify below) \| |
| \|  \|  \|  \|  \| a16_other \| \| --- \| --- \| --- \| --- \| --- \| | Please specify 'Other' toilet facility: |  |
| \|  \|  \|  \| a17 (required) \| \| --- \| --- \| --- \| --- \| | Do you share this toilet facility with other households? | \|  \| 1 \| Yes \| \| --- \| --- \| --- \| \|  \| 2 \| No \| \|  \| 99 \| REFUSED \| |
| \|  \|  \|  \| reachable_group > over18_group > hiv_positive > fuel \| \| --- \| --- \| --- \| --- \| | | |
| \|  \|  \|  \|  \| a18 (required) \| \| --- \| --- \| --- \| --- \| --- \| | What type of fuel does your household mainly use for cooking? | \|  \| 1 \| Electricity from grid \| \| --- \| --- \| --- \| \|  \| 2 \| Electricity from generator \| \|  \| 3 \| Electricity from solar energy \| \|  \| 4 \| Charcoal \| \|  \| 5 \| Wood \| \|  \| 6 \| Gas \| \|  \| 7 \| Paraffin \| \|  \| 8 \| Coal \| \|  \| 9 \| No food cooked in the household \| \|  \| 99 \| REFUSED \| \|  \| 10 \| Other (specify below) \| |
| \|  \|  \|  \|  \| a18_other \| \| --- \| --- \| --- \| --- \| --- \| | Please specify 'Other' household cooking fuel: |  |
| \|  \|  \|  \| reachable_group > over18_group > hiv_positive > ownrent \| \| --- \| --- \| --- \| --- \| | | |
| \|  \|  \|  \|  \| a19 (required) \| \| --- \| --- \| --- \| --- \| --- \| | Does your household own or rent your homestead? | \|  \| 1 \| Own \| \| --- \| --- \| --- \| \|  \| 2 \| Rent \| \|  \| 99 \| REFUSED \| \|  \| 3 \| Other (specify below) \| |
| \|  \|  \|  \|  \| a19_other \| \| --- \| --- \| --- \| --- \| --- \| | Please specify 'Other' if household does not own or rent: |  |
| \|  \|  \|  \| a20 (required) \| \| --- \| --- \| --- \| --- \| | Overall, how satisfied or dissatisfied are you with the healthcare services in your area?  Ask the respondent to refer to scale 1. Ensure that respondent knows 0=Very dissatisfied, and 10=Very satisfied. | \|  \| 0 \| 0 (Very dissatisfied) \| \| --- \| --- \| --- \| \|  \| 1 \| 1 \| \|  \| 2 \| 2 \| \|  \| 3 \| 3 \| \|  \| 4 \| 4 \| \|  \| 5 \| 5 \| \|  \| 6 \| 6 \| \|  \| 7 \| 7 \| \|  \| 8 \| 8 \| \|  \| 9 \| 9 \| \|  \| 10 \| 10 (Very satisfied) \| \|  \| 99 \| REFUSED \| |
| \|  \|  \|  \| a21 (required) \| \| --- \| --- \| --- \| --- \| | How satisfied or dissatisfied are you with the healthcare services for HIV in your area?  Ask the respondent to refer to scale 1. Ensure that respondent knows 0=Very dissatisfied, and 10=Very satisfied. | \|  \| 0 \| 0 (Very dissatisfied) \| \| --- \| --- \| --- \| \|  \| 1 \| 1 \| \|  \| 2 \| 2 \| \|  \| 3 \| 3 \| \|  \| 4 \| 4 \| \|  \| 5 \| 5 \| \|  \| 6 \| 6 \| \|  \| 7 \| 7 \| \|  \| 8 \| 8 \| \|  \| 9 \| 9 \| \|  \| 10 \| 10 (Very satisfied) \| \|  \| 99 \| REFUSED \| |
| \|  \|  \|  \| reachable_group > over18_group > hiv_positive > groupb1 \| \| --- \| --- \| --- \| --- \| | | |
| \|  \|  \|  \|  \| note_b \| \| --- \| --- \| --- \| --- \| --- \| | PART 2: HIV STATUS DISCLOSURE AND SELF-REPORTED ART ADHERENCE |  |
| \|  \|  \|  \|  \| b1 (required) \| \| --- \| --- \| --- \| --- \| --- \| | Have you ever informed anyone about your HIV status? | \|  \| 1 \| Yes \| \| --- \| --- \| --- \| \|  \| 0 \| No \| \|  \| 2 \| DON'T KNOW \| \|  \| 3 \| REFUSED \| |
| \|  \|  \|  \| reachable_group > over18_group > hiv_positive > disclosure \| \| --- \| --- \| --- \| --- \| | | |
| \|  \|  \|  \|  \| b2 (required) \| \| --- \| --- \| --- \| --- \| --- \| | To whom did you inform about your HIV status?  Read out each option and select all that apply. Probe with: Anyone else? | \|  \| 1 \| Spouse / Current partner \| \| --- \| --- \| --- \| \|  \| 2 \| Girlfriend / Boyfriend \| \|  \| 3 \| Parent \| \|  \| 4 \| Brother / Sister \| \|  \| 5 \| Someone else in the family \| \|  \| 6 \| Friend \| \|  \| 7 \| Religious Leader \| \|  \| 99 \| REFUSED \| \|  \| 97 \| Other (specify below) \| |
| \|  \|  \|  \|  \| b2_other \| \| --- \| --- \| --- \| --- \| --- \| | If you selected 'Other', please specify: |  |
| \|  \|  \|  \| b3 (required) \| \| --- \| --- \| --- \| --- \| | In total, to how many people have you disclosed your HIV status?  Please enter the number of people. |  |
| \|  \|  \|  \| b4 (required) \| \| --- \| --- \| --- \| --- \| | When did you first start taking ARVs?  If respondent DK, enter February 1980. If respondent RF, enter March 1980. |  |
| \|  \|  \|  \| b6 (required) \| \| --- \| --- \| --- \| --- \| | Were you pregnant when you first started taking ARVs? | \|  \| 1 \| Yes \| \| --- \| --- \| --- \| \|  \| 2 \| No \| \|  \| 99 \| REFUSED \| |
| \|  \|  \|  \| b7 (required) \| \| --- \| --- \| --- \| --- \| | Did you continue taking ARVs after pregnancy? | \|  \| 1 \| Yes \| \| --- \| --- \| --- \| \|  \| 2 \| No \| \|  \| 99 \| REFUSED \| |
| \|  \|  \|  \| b8 (required) \| \| --- \| --- \| --- \| --- \| | How would you rate your adherence to ARVs over the last month? Please answer with very poor, poor, fair, good, very good, or excellent. | \|  \| 1 \| Very poor \| \| --- \| --- \| --- \| \|  \| 2 \| Poor \| \|  \| 3 \| Fair \| \|  \| 4 \| Good \| \|  \| 5 \| Very good \| \|  \| 6 \| Excellent \| \|  \| 98 \| DON'T KNOW \| \|  \| 99 \| REFUSED \| |
| \|  \|  \|  \| b9-13 \| \| --- \| --- \| --- \| --- \| | Please answer the next five questions with ‘very often’, ‘often’, ‘sometimes’ ‘rarely’, or ‘never’. |  |
| \|  \|  \|  \| reachable_group > over18_group > hiv_positive > often \| \| --- \| --- \| --- \| --- \| | | |
| \|  \|  \|  \|  \| reachable_group > over18_group > hiv_positive > often > b9group \| \| --- \| --- \| --- \| --- \| --- \| | | |
| \|  \|  \|  \|  \|  \| b9 (required) \| \| --- \| --- \| --- \| --- \| --- \| --- \| | Some people forget to take their ARVs. In the last one month, how often did this happen to you? | \|  \| 1 \| Very often \| \| --- \| --- \| --- \| \|  \| 2 \| Often \| \|  \| 3 \| Sometimes \| \|  \| 4 \| Rarely \| \|  \| 5 \| Never \| \|  \| 98 \| DON'T KNOW \| \|  \| 99 \| REFUSED \| |
| \|  \|  \|  \|  \|  \| b10 (required) \| \| --- \| --- \| --- \| --- \| --- \| --- \| | Some people miss out a dose of their ARVs or adjust it to suit their own needs. In the last one month, how often did you do this? | \|  \| 1 \| Very often \| \| --- \| --- \| --- \| \|  \| 2 \| Often \| \|  \| 3 \| Sometimes \| \|  \| 4 \| Rarely \| \|  \| 5 \| Never \| \|  \| 98 \| DON'T KNOW \| \|  \| 99 \| REFUSED \| |
| \|  \|  \|  \|  \| reachable_group > over18_group > hiv_positive > often > groupb11 \| \| --- \| --- \| --- \| --- \| --- \| | | |
| \|  \|  \|  \|  \|  \| b11 (required) \| \| --- \| --- \| --- \| --- \| --- \| --- \| | Some people stop taking their ARVs when they feel better. In the last one month, how often did you do this? | \|  \| 1 \| Very often \| \| --- \| --- \| --- \| \|  \| 2 \| Often \| \|  \| 3 \| Sometimes \| \|  \| 4 \| Rarely \| \|  \| 5 \| Never \| \|  \| 98 \| DON'T KNOW \| \|  \| 99 \| REFUSED \| |
| \|  \|  \|  \|  \|  \| b12 (required) \| \| --- \| --- \| --- \| --- \| --- \| --- \| | Some people stop taking their ARVs when they feel worse. In the last one month, how often did you do this? | \|  \| 1 \| Very often \| \| --- \| --- \| --- \| \|  \| 2 \| Often \| \|  \| 3 \| Sometimes \| \|  \| 4 \| Rarely \| \|  \| 5 \| Never \| \|  \| 98 \| DON'T KNOW \| \|  \| 99 \| REFUSED \| |
| \|  \|  \|  \|  \| b13 (required) \| \| --- \| --- \| --- \| --- \| --- \| | Some people miss their clinic appointment to pick up their ARVs. In the last six months, how often did you do this? | \|  \| 1 \| Very often \| \| --- \| --- \| --- \| \|  \| 2 \| Often \| \|  \| 3 \| Sometimes \| \|  \| 4 \| Rarely \| \|  \| 5 \| Never \| \|  \| 98 \| DON'T KNOW \| \|  \| 99 \| REFUSED \| |
| \|  \|  \|  \| b14 (required) \| \| --- \| --- \| --- \| --- \| | In the last six months, how many times did you miss your appointment to pick up your ARVs? | \|  \| 1 \| One \| \| --- \| --- \| --- \| \|  \| 2 \| Two \| \|  \| 3 \| Three \| \|  \| 4 \| Four \| \|  \| 5 \| Five \| \|  \| 6 \| Six or more \| \|  \| 7 \| None \| \|  \| 98 \| DON'T KNOW \| \|  \| 99 \| REFUSED \| |
| \|  \|  \|  \| b15 (required) \| \| --- \| --- \| --- \| --- \| | What was the reason or the reasons for missing the visits?  DO NOT READ THE LIST ALOUD; PROBE WITH: "ANYTHING ELSE?" Select all that apply. | \|  \| 1 \| Lack of money \| \| --- \| --- \| --- \| \|  \| 2 \| Lack of time \| \|  \| 3 \| I felt better \| \|  \| 4 \| I could not take time off from work \| \|  \| 5 \| No transport \| \|  \| 6 \| Too ill to travel \| \|  \| 7 \| Other responsibilities \| \|  \| 8 \| The treatment is not effective / does not make me feel better \| \|  \| 9 \| The queues in the facility are too long \| \|  \| 10 \| The staff are rude or uncaring \| \|  \| 11 \| I have had bad experiences with the staff in the past \| \|  \| 97 \| Other (SPECIFY ON THE NEXT PAGE) \| \|  \| 98 \| Don't know \| \|  \| 99 \| Refused \| |
| \|  \|  \|  \| b15_other (required) \| \| --- \| --- \| --- \| --- \| | PLEASE SPECIFY 'Other' REASON |  |
| \|  \|  \|  \| b16_pre (required) \| \| --- \| --- \| --- \| --- \| | Do you know the date when you last picked up your ARVs from the healthcare facility? | \|  \| 1 \| Yes \| \| --- \| --- \| --- \| \|  \| 2 \| No \| \|  \| 99 \| REFUSED \| |
| \|  \|  \|  \| b16 (required) \| \| --- \| --- \| --- \| --- \| | Before today, when did you last pick up your ARVs from the healthcare facility?  Please enter the date. If RF, enter Jan 1, 1980. If never picked up ARVs, enter February 1, 1980. |  |
| \|  \|  \|  \| b16_dk (required) \| \| --- \| --- \| --- \| --- \| | DOES THE RESPONDENT KNOW HOW MANY WEEKS OR DAYS AGO HE/SHE PICKED UP THE ARVS? | \|  \| 1 \| Weeks \| \| --- \| --- \| --- \| \|  \| 2 \| Days \| \|  \| 98 \| Still don't know \| |
| \|  \|  \|  \| b16_dk_weeks (required) \| \| --- \| --- \| --- \| --- \| | How many weeks ago did you pick up your ARVs from the healthcare facility?  If RF, enter 9999. |  |
| \|  \|  \|  \| b16_dk_days (required) \| \| --- \| --- \| --- \| --- \| | How many days ago did you pick up your ARVs from the healthcare facility?  If RF, enter 9999. |  |
| \|  \|  \|  \| b17 (required) \| \| --- \| --- \| --- \| --- \| | How frequently are you supposed to go to the healthcare facility to pick up your ARVs? | \|  \| 1 \| Once a month \| \| --- \| --- \| --- \| \|  \| 2 \| Once every 3 months \| \|  \| 3 \| Once every 6 months \| \|  \| 4 \| My ARVs are brought to my home by a home-based carer \| \|  \| 97 \| Other (specify below) \| \|  \| 99 \| REFUSED \| |
| \|  \|  \|  \| b17_other (required) \| \| --- \| --- \| --- \| --- \| | Please specify 'Other' frequency: |  |
| \|  \|  \|  \| note_c \| \| --- \| --- \| --- \| --- \| | PART 3: HEALTH SERVICE UTILIZATION AND EXPENDITURE  I would now like to ask you a few questions about the costs you experience to access healthcare. This question is only about the cost for YOUR OWN healthcare, NOT for other people in your household. |  |
| \|  \|  \|  \| c1 (required) \| \| --- \| --- \| --- \| --- \| | Are you covered by a scheme, such as an insurance, that helps you pay for healthcare? | \|  \| 1 \| Yes \| \| --- \| --- \| --- \| \|  \| 2 \| No \| \|  \| 99 \| REFUSED \| |
| \|  \|  \|  \| c2 (required) \| \| --- \| --- \| --- \| --- \| | For your clinic visit TODAY, what type of expenses did you pay for?  Ensure that the respondent only counts expenses since he/she started travelling to the clinic until the time of the interview. | \|  \| 1 \| Consultation fees \| \| --- \| --- \| --- \| \|  \| 2 \| Medical tests \| \|  \| 3 \| Medicines \| \|  \| 4 \| Transport to get to the clinic (one way) \| \|  \| 5 \| Payment for someone to look after your children while you are gone to the clinic \| \|  \| 6 \| Food since you started travelling to the clinic until the time of the interview \| \|  \| 7 \| Phone calls/SMS since you started travelling to the clinic until the time of the interview \| \|  \| 8 \| Others (specify on next page) \| \|  \| 99 \| REFUSED \| |
| \|  \|  \|  \| c2_other (required) \| \| --- \| --- \| --- \| --- \| | Please specify 'Other' clinic expenses: |  |
| \|  \|  \|  \| c2_a (required) \| \| --- \| --- \| --- \| --- \| | How much did you pay for: Consultation fee?  Please enter the amount in Tanzanian Shilling. If DK, enter -98. If RF, enter -99. |  |
| \|  \|  \|  \| c2_b (required) \| \| --- \| --- \| --- \| --- \| | How much did you pay for: Medical tests?  Please enter the amount in Tanzanian Shilling. If DK, enter -98. If RF, enter -99. |  |
| \|  \|  \|  \| c2_c (required) \| \| --- \| --- \| --- \| --- \| | How much did you pay for: Medicines?  Please enter the amount in Tanzanian Shilling. If DK, enter -98. If RF, enter -99. |  |
| \|  \|  \|  \| c2_d (required) \| \| --- \| --- \| --- \| --- \| | How much did you pay for: Transport to get to the clinic (one way)?  Please enter the amount in Tanzanian Shilling. If DK, enter -98. If RF, enter -99. |  |
| \|  \|  \|  \| c2_e (required) \| \| --- \| --- \| --- \| --- \| | How much did you pay for: Payment for someone to look after your children while you are gone to the clinic?  Please enter the amount in Tanzanian Shilling. If DK, enter -98. If RF, enter -99. |  |
| \|  \|  \|  \| c2_f (required) \| \| --- \| --- \| --- \| --- \| | How much did you pay for: Food since you started travelling to the clinic until the time of the interview?  Please enter the amount in Tanzanian Shilling. If DK, enter -98. If RF, enter -99. |  |
| \|  \|  \|  \| c2_g (required) \| \| --- \| --- \| --- \| --- \| | How much did you pay for: Phone calls/SMS since you started travelling to the clinic until the time of the interview  Please enter the amount in Tanzanian Shilling. If DK, enter -98. If RF, enter -99. |  |
| \|  \|  \|  \| c2_h (required) \| \| --- \| --- \| --- \| --- \| | How much did you pay for: Others?  Please enter the amount in Tanzanian Shilling. If DK, enter -98. If RF, enter -99. |  |
| \|  \|  \|  \| c3 (required) \| \| --- \| --- \| --- \| --- \| | In the past 12 months, were you admitted to a hospital? With being admitted to a hospital, I mean that you slept in the hospital and did not just come there for a few hours. | \|  \| 1 \| Yes \| \| --- \| --- \| --- \| \|  \| 2 \| No \| \|  \| 99 \| REFUSED \| |
| \|  \|  \|  \| c4 (required) \| \| --- \| --- \| --- \| --- \| | How many times were you admitted to hospital in the last 12 months?  Please record the number of times. If DON'T KNOW enter '8888.' If REFUSED enter '9999' |  |
| \|  \|  \|  \| reachable_group > over18_group > hiv_positive > Hospital stay (1) \| \| --- \| --- \| --- \| --- \| | | (Repeated group) |
| \|  \|  \|  \|  \| reachable_group > over18_group > hiv_positive > Hospital stay (1) > hosp_descript \| \| --- \| --- \| --- \| --- \| --- \| | | |
| \|  \|  \|  \|  \|  \| c5_note \| \| --- \| --- \| --- \| --- \| --- \| --- \| | For hospital admission # 1, please write the date, number of nights you spent in hospital, and name of the facility. |  |
| \|  \|  \|  \|  \|  \| c5_a (required) \| \| --- \| --- \| --- \| --- \| --- \| --- \| | Date of hospital admission  If respondent DK, enter February 1st, 1980. If respondent RF, enter March 1st, 1980. |  |
| \|  \|  \|  \|  \|  \| c5_b (required) \| \| --- \| --- \| --- \| --- \| --- \| --- \| | Number of nights spent in the hospital  If respondent DK, enter '8888'. If respondent RF, enter '9999'. |  |
| \|  \|  \|  \|  \|  \| c5_c (required) \| \| --- \| --- \| --- \| --- \| --- \| --- \| | Name of the facility |  |
| \|  \|  \|  \|  \| reachable_group > over18_group > hiv_positive > Hospital stay (1) > admission_cost \| \| --- \| --- \| --- \| --- \| --- \| | | |
| \|  \|  \|  \|  \|  \| reachable_group > over18_group > hiv_positive > Hospital stay (1) > admission_cost > c5_group1 \| \| --- \| --- \| --- \| --- \| --- \| --- \| | | |
| \|  \|  \|  \|  \|  \|  \| c5_note2 \| \| --- \| --- \| --- \| --- \| --- \| --- \| --- \| | For hospital admission # 1, how much did you pay for:  Please record the answers in TSh. If DK, enter -98. If RF, enter -99. |  |
| \|  \|  \|  \|  \|  \|  \| c5_d1 (required) \| \| --- \| --- \| --- \| --- \| --- \| --- \| --- \| | Hospital/consultation fees? |  |
| \|  \|  \|  \|  \|  \|  \| c5_d2 (required) \| \| --- \| --- \| --- \| --- \| --- \| --- \| --- \| | Surgery? |  |
| \|  \|  \|  \|  \|  \|  \| c5_d3 (required) \| \| --- \| --- \| --- \| --- \| --- \| --- \| --- \| | Medical tests (e.g. blood tests and X-rays) ? |  |
| \|  \|  \|  \|  \|  \|  \| c5_d4 (required) \| \| --- \| --- \| --- \| --- \| --- \| --- \| --- \| | Medicines? |  |
| \|  \|  \|  \|  \|  \|  \| c5_d5 (required) \| \| --- \| --- \| --- \| --- \| --- \| --- \| --- \| | Other hospital fees? |  |
| \|  \|  \|  \|  \|  \|  \| c5_d6 (required) \| \| --- \| --- \| --- \| --- \| --- \| --- \| --- \| | Transport including ambulance charges? |  |
| \|  \|  \|  \|  \|  \| reachable_group > over18_group > hiv_positive > Hospital stay (1) > admission_cost > c5_group2 \| \| --- \| --- \| --- \| --- \| --- \| --- \| | | |
| \|  \|  \|  \|  \|  \|  \| c5_note3 \| \| --- \| --- \| --- \| --- \| --- \| --- \| --- \| | For hospital admission # 1, how much did you pay for:  Please record the answers in TSh. If DK, enter -98. If RF, enter -99. |  |
| \|  \|  \|  \|  \|  \|  \| c5_d7 (required) \| \| --- \| --- \| --- \| --- \| --- \| --- \| --- \| | Payment for someone to look after your child while you were in hospital? |  |
| \|  \|  \|  \|  \|  \|  \| c5_d8 (required) \| \| --- \| --- \| --- \| --- \| --- \| --- \| --- \| | Payment for someone to look after your house, garden/fields or animals while you were in hospital? |  |
| \|  \|  \|  \|  \|  \|  \| c5_d9 (required) \| \| --- \| --- \| --- \| --- \| --- \| --- \| --- \| | Food? |  |
| \|  \|  \|  \|  \|  \|  \| c5_d10 (required) \| \| --- \| --- \| --- \| --- \| --- \| --- \| --- \| | Phone calls/SMS? |  |
| \|  \|  \|  \|  \|  \|  \| c5_d11 (required) \| \| --- \| --- \| --- \| --- \| --- \| --- \| --- \| | Accommodation if you needed to stay the night nearby? |  |
| \|  \|  \|  \|  \|  \|  \| c5_d12 \| \| --- \| --- \| --- \| --- \| --- \| --- \| --- \| | Other? (SPECIFY) |  |
| \|  \|  \|  \|  \| c5_d12other \| \| --- \| --- \| --- \| --- \| --- \| | Please specify if 'Other'. |  |
| \|  \|  \|  \| reachable_group > over18_group > hiv_positive > c6 \| \| --- \| --- \| --- \| --- \| | | |
| \|  \|  \|  \|  \| c6_label \| \| --- \| --- \| --- \| --- \| --- \| | The following questions ask about healthcare you accessed in the past six months. |  |
| \|  \|  \|  \|  \| c6_labels \| \| --- \| --- \| --- \| --- \| --- \| | In the past SIX MONTHS, have you visited… | \|  \| 1 \| Yes \| \| --- \| --- \| --- \| \|  \| 2 \| No \| \|  \| 99 \| REFUSED \| |
| \|  \|  \|  \|  \| c6_a (required) \| \| --- \| --- \| --- \| --- \| --- \| | A public primary care clinic? | \|  \| 1 \| Yes \| \| --- \| --- \| --- \| \|  \| 2 \| No \| \|  \| 99 \| REFUSED \| |
| \|  \|  \|  \|  \| c6_b (required) \| \| --- \| --- \| --- \| --- \| --- \| | A private doctor? | \|  \| 1 \| Yes \| \| --- \| --- \| --- \| \|  \| 2 \| No \| \|  \| 99 \| REFUSED \| |
| \|  \|  \|  \|  \| c6_c (required) \| \| --- \| --- \| --- \| --- \| --- \| | Chemist / pharmacy? | \|  \| 1 \| Yes \| \| --- \| --- \| --- \| \|  \| 2 \| No \| \|  \| 99 \| REFUSED \| |
| \|  \|  \|  \|  \| c6_d (required) \| \| --- \| --- \| --- \| --- \| --- \| | Traditional healer? | \|  \| 1 \| Yes \| \| --- \| --- \| --- \| \|  \| 2 \| No \| \|  \| 99 \| REFUSED \| |
| \|  \|  \|  \|  \| c6_e (required) \| \| --- \| --- \| --- \| --- \| --- \| | Diviner? | \|  \| 1 \| Yes \| \| --- \| --- \| --- \| \|  \| 2 \| No \| \|  \| 99 \| REFUSED \| |
| \|  \|  \|  \|  \| c6_f (required) \| \| --- \| --- \| --- \| --- \| --- \| | A faith healer? | \|  \| 1 \| Yes \| \| --- \| --- \| --- \| \|  \| 2 \| No \| \|  \| 99 \| REFUSED \| |
| \|  \|  \|  \| c6_a1 (required) \| \| --- \| --- \| --- \| --- \| | In the past SIX MONTHS, how many times did you visit: a public primary care clinic?  If RF, enter -99. |  |
| \|  \|  \|  \| c6_error \| \| --- \| --- \| --- \| --- \| | NOTE: YOU INPUT 0 VISITS, BUT IN A PREVIOUS QUESTION YOU INPUT THAT THE RESPONDENT VISITED THIS FACILITY / HEALER IN THE PAST MONTH. PLEASE GO BACK AND CONFIRM THE RESPONSES. |  |
| \|  \|  \|  \| reachable_group > over18_group > hiv_positive > c6_1 \| \| --- \| --- \| --- \| --- \| | | |
| \|  \|  \|  \|  \| reachable_group > over18_group > hiv_positive > c6_1 > c6_a1group \| \| --- \| --- \| --- \| --- \| --- \| | | |
| \|  \|  \|  \|  \|  \| c6_a1label \| \| --- \| --- \| --- \| --- \| --- \| --- \| | For the last public primary care clinic visit, how much did you pay for:  Please record the answers in TSh. If DK, enter -98. If RF, enter -99. |  |
| \|  \|  \|  \|  \|  \| c6_a2 (required) \| \| --- \| --- \| --- \| --- \| --- \| --- \| | Clinic/consultation fee |  |
| \|  \|  \|  \|  \|  \| c6_a3 (required) \| \| --- \| --- \| --- \| --- \| --- \| --- \| | Medical tests (e.g. blood tests and X-rays) |  |
| \|  \|  \|  \|  \|  \| c6_a4 (required) \| \| --- \| --- \| --- \| --- \| --- \| --- \| | Medicines |  |
| \|  \|  \|  \|  \|  \| c6_a5 (required) \| \| --- \| --- \| --- \| --- \| --- \| --- \| | Transport |  |
| \|  \|  \|  \|  \|  \| c6_a6 (required) \| \| --- \| --- \| --- \| --- \| --- \| --- \| | Payment for someone to look after your child while you were gone |  |
| \|  \|  \|  \|  \| reachable_group > over18_group > hiv_positive > c6_1 > c6_a7group \| \| --- \| --- \| --- \| --- \| --- \| | | |
| \|  \|  \|  \|  \|  \| c6_a7label \| \| --- \| --- \| --- \| --- \| --- \| --- \| | For the last public primary care clinic visit, how much did you pay for:  Please record the answers in TSh. If DK, enter -98. If RF, enter -99. |  |
| \|  \|  \|  \|  \|  \| c6_a7 (required) \| \| --- \| --- \| --- \| --- \| --- \| --- \| | Payment for someone to look after your house, garden/fields or animals while you were gone |  |
| \|  \|  \|  \|  \|  \| c6_a8 (required) \| \| --- \| --- \| --- \| --- \| --- \| --- \| | Food |  |
| \|  \|  \|  \|  \|  \| c6_a9 (required) \| \| --- \| --- \| --- \| --- \| --- \| --- \| | Phone calls/SMS |  |
| \|  \|  \|  \|  \|  \| c6_a10 (required) \| \| --- \| --- \| --- \| --- \| --- \| --- \| | Accommodation if you needed to stay the night nearby |  |
| \|  \|  \|  \|  \|  \| c6_a11 (required) \| \| --- \| --- \| --- \| --- \| --- \| --- \| | Other (SPECIFY ON THE NEXT PAGE) |  |
| \|  \|  \|  \| c6_a11_other (required) \| \| --- \| --- \| --- \| --- \| | Please specify 'Other' payment related to primary care clinic visit |  |
| \|  \|  \|  \| c6_b1 (required) \| \| --- \| --- \| --- \| --- \| | In the past SIX MONTHS, how many times did you visit: a private doctor?  If RF, enter -99. |  |
| \|  \|  \|  \| c6_errorb \| \| --- \| --- \| --- \| --- \| | NOTE: YOU INPUT 0 VISITS, BUT IN A PREVIOUS QUESTION YOU INPUT THAT THE RESPONDENT VISITED THIS FACILITY / HEALER IN THE PAST MONTH. PLEASE GO BACK AND CONFIRM THE RESPONSES. |  |
| \|  \|  \|  \| reachable_group > over18_group > hiv_positive > c6_3 \| \| --- \| --- \| --- \| --- \| | | |
| \|  \|  \|  \|  \| reachable_group > over18_group > hiv_positive > c6_3 > c6_b1group \| \| --- \| --- \| --- \| --- \| --- \| | | |
| \|  \|  \|  \|  \|  \| c6_b1label \| \| --- \| --- \| --- \| --- \| --- \| --- \| | For the last private doctor visit, how much did you spend for:  Please record the answers in TSh. If DK, enter -98. If RF, enter -99. |  |
| \|  \|  \|  \|  \|  \| c6_b2 (required) \| \| --- \| --- \| --- \| --- \| --- \| --- \| | Clinic/consultation fee |  |
| \|  \|  \|  \|  \|  \| c6_b3 (required) \| \| --- \| --- \| --- \| --- \| --- \| --- \| | Medical tests (e.g. blood tests and X-rays) |  |
| \|  \|  \|  \|  \|  \| c6_b4 (required) \| \| --- \| --- \| --- \| --- \| --- \| --- \| | Medicines |  |
| \|  \|  \|  \|  \|  \| c6_b5 (required) \| \| --- \| --- \| --- \| --- \| --- \| --- \| | Transport |  |
| \|  \|  \|  \|  \|  \| c6_b6 (required) \| \| --- \| --- \| --- \| --- \| --- \| --- \| | Payment for someone to look after your child while you were gone |  |
| \|  \|  \|  \|  \| reachable_group > over18_group > hiv_positive > c6_3 > c6_b7group \| \| --- \| --- \| --- \| --- \| --- \| | | |
| \|  \|  \|  \|  \|  \| c6_b7label \| \| --- \| --- \| --- \| --- \| --- \| --- \| | For the last private doctor visit, how much did you spend for:  Please record the answers in TSh. If DK, enter -98. If RF, enter -99. |  |
| \|  \|  \|  \|  \|  \| c6_b7 (required) \| \| --- \| --- \| --- \| --- \| --- \| --- \| | Payment for someone to look after your house, garden/fields or animals while you were gone |  |
| \|  \|  \|  \|  \|  \| c6_b8 (required) \| \| --- \| --- \| --- \| --- \| --- \| --- \| | Food |  |
| \|  \|  \|  \|  \|  \| c6_b9 (required) \| \| --- \| --- \| --- \| --- \| --- \| --- \| | Phone calls/SMS |  |
| \|  \|  \|  \|  \|  \| c6_b10 (required) \| \| --- \| --- \| --- \| --- \| --- \| --- \| | Accommodation if you needed to stay the night nearby |  |
| \|  \|  \|  \|  \|  \| c6_b11 (required) \| \| --- \| --- \| --- \| --- \| --- \| --- \| | Other (specify below) |  |
| \|  \|  \|  \| c6_b11_other (required) \| \| --- \| --- \| --- \| --- \| | Please specify 'Other' payment related to private doctor visit(s) |  |
| \|  \|  \|  \| c6_c1 (required) \| \| --- \| --- \| --- \| --- \| | In the past SIX MONTHS, how many times did you visit: a chemist / pharmacy?  If RF, enter -99. |  |
| \|  \|  \|  \| c6_errorc \| \| --- \| --- \| --- \| --- \| | NOTE: YOU INPUT 0 VISITS, BUT IN A PREVIOUS QUESTION YOU INPUT THAT THE RESPONDENT VISITED THIS FACILITY / HEALER IN THE PAST MONTH. PLEASE GO BACK AND CONFIRM THE RESPONSES. |  |
| \|  \|  \|  \| reachable_group > over18_group > hiv_positive > c6_4 \| \| --- \| --- \| --- \| --- \| | | |
| \|  \|  \|  \|  \| reachable_group > over18_group > hiv_positive > c6_4 > c6_c1group \| \| --- \| --- \| --- \| --- \| --- \| | | |
| \|  \|  \|  \|  \|  \| c6_c1label \| \| --- \| --- \| --- \| --- \| --- \| --- \| | For the last Chemist / pharmacy visit, how much did you spend for:  Please record the answers in TSh. If DK, enter -98. If RF, enter -99. |  |
| \|  \|  \|  \|  \|  \| c6_c2 (required) \| \| --- \| --- \| --- \| --- \| --- \| --- \| | Clinic/consultation fee |  |
| \|  \|  \|  \|  \|  \| c6_c3 (required) \| \| --- \| --- \| --- \| --- \| --- \| --- \| | Medical tests (e.g. blood tests and X-rays) |  |
| \|  \|  \|  \|  \|  \| c6_c4 (required) \| \| --- \| --- \| --- \| --- \| --- \| --- \| | Medicines |  |
| \|  \|  \|  \|  \|  \| c6_c5 (required) \| \| --- \| --- \| --- \| --- \| --- \| --- \| | Transport |  |
| \|  \|  \|  \|  \|  \| c6_c6 (required) \| \| --- \| --- \| --- \| --- \| --- \| --- \| | Payment for someone to look after your child while you were gone |  |
| \|  \|  \|  \|  \| reachable_group > over18_group > hiv_positive > c6_4 > c6_c7group \| \| --- \| --- \| --- \| --- \| --- \| | | |
| \|  \|  \|  \|  \|  \| c6_c7label \| \| --- \| --- \| --- \| --- \| --- \| --- \| | For the last Chemist / pharmacy visit, how much did you spend for:  Please record the answers in TSh. If DK, enter -98. If RF, enter -99. |  |
| \|  \|  \|  \|  \|  \| c6_c7 (required) \| \| --- \| --- \| --- \| --- \| --- \| --- \| | Payment for someone to look after your house, garden/fields or animals while you were gone |  |
| \|  \|  \|  \|  \|  \| c6_c8 (required) \| \| --- \| --- \| --- \| --- \| --- \| --- \| | Food |  |
| \|  \|  \|  \|  \|  \| c6_c9 (required) \| \| --- \| --- \| --- \| --- \| --- \| --- \| | Phone calls/SMS |  |
| \|  \|  \|  \|  \|  \| c6_c10 (required) \| \| --- \| --- \| --- \| --- \| --- \| --- \| | Accommodation if you needed to stay the night nearby |  |
| \|  \|  \|  \|  \|  \| c6_c11 (required) \| \| --- \| --- \| --- \| --- \| --- \| --- \| | Other (specify below) |  |
| \|  \|  \|  \| c6_c11_other (required) \| \| --- \| --- \| --- \| --- \| | Please specify 'Other' payment related to Chemist / pharmacy(s) |  |
| \|  \|  \|  \| c6_d1 (required) \| \| --- \| --- \| --- \| --- \| | In the past SIX MONTHS, how many times did you visit: a traditional healer?  If RF, enter -99. |  |
| \|  \|  \|  \| c6_errord \| \| --- \| --- \| --- \| --- \| | NOTE: YOU INPUT 0 VISITS, BUT IN A PREVIOUS QUESTION YOU INPUT THAT THE RESPONDENT VISITED THIS FACILITY / HEALER IN THE PAST MONTH. PLEASE GO BACK AND CONFIRM THE RESPONSES. |  |
| \|  \|  \|  \| reachable_group > over18_group > hiv_positive > c6_5 \| \| --- \| --- \| --- \| --- \| | | |
| \|  \|  \|  \|  \| reachable_group > over18_group > hiv_positive > c6_5 > c6_d1group \| \| --- \| --- \| --- \| --- \| --- \| | | |
| \|  \|  \|  \|  \|  \| c6_d1label \| \| --- \| --- \| --- \| --- \| --- \| --- \| | For the last Traditional healer visit, how much did you spend for:  Please record the answers in TSh. If DK, enter -98. If RF, enter -99. |  |
| \|  \|  \|  \|  \|  \| c6_d2 (required) \| \| --- \| --- \| --- \| --- \| --- \| --- \| | Clinic/consultation fee |  |
| \|  \|  \|  \|  \|  \| c6_d3 (required) \| \| --- \| --- \| --- \| --- \| --- \| --- \| | Medical tests (e.g. blood tests and X-rays) |  |
| \|  \|  \|  \|  \|  \| c6_d4 (required) \| \| --- \| --- \| --- \| --- \| --- \| --- \| | Medicines |  |
| \|  \|  \|  \|  \|  \| c6_d5 (required) \| \| --- \| --- \| --- \| --- \| --- \| --- \| | Transport |  |
| \|  \|  \|  \|  \|  \| c6_d6 (required) \| \| --- \| --- \| --- \| --- \| --- \| --- \| | Payment for someone to look after your child while you were gone |  |
| \|  \|  \|  \|  \| reachable_group > over18_group > hiv_positive > c6_5 > c6_d7group \| \| --- \| --- \| --- \| --- \| --- \| | | |
| \|  \|  \|  \|  \|  \| c6_d7label \| \| --- \| --- \| --- \| --- \| --- \| --- \| | For the last Traditional healer visit, how much did you spend for:  Please record the answers in TSh. If DK, enter -98. If RF, enter -99. |  |
| \|  \|  \|  \|  \|  \| c6_d7 (required) \| \| --- \| --- \| --- \| --- \| --- \| --- \| | Payment for someone to look after your house, garden/fields or animals while you were gone |  |
| \|  \|  \|  \|  \|  \| c6_d8 (required) \| \| --- \| --- \| --- \| --- \| --- \| --- \| | Food |  |
| \|  \|  \|  \|  \|  \| c6_d9 (required) \| \| --- \| --- \| --- \| --- \| --- \| --- \| | Phone calls/SMS |  |
| \|  \|  \|  \|  \|  \| c6_d10 (required) \| \| --- \| --- \| --- \| --- \| --- \| --- \| | Accommodation if you needed to stay the night nearby |  |
| \|  \|  \|  \|  \|  \| c6_d11 (required) \| \| --- \| --- \| --- \| --- \| --- \| --- \| | Other (SPECIFY ON THE NEXT PAGE) |  |
| \|  \|  \|  \| c6_d11_other (required) \| \| --- \| --- \| --- \| --- \| | Please specify 'Other' payment related to Traditional healer visit(s) |  |
| \|  \|  \|  \| c6_e1 (required) \| \| --- \| --- \| --- \| --- \| | In the past SIX MONTHS, how many times did you visit: a diviner?  If RF, enter -99. |  |
| \|  \|  \|  \| c6_errore \| \| --- \| --- \| --- \| --- \| | NOTE: YOU INPUT 0 VISITS, BUT IN A PREVIOUS QUESTION YOU INPUT THAT THE RESPONDENT VISITED THIS FACILITY / HEALER IN THE PAST MONTH. PLEASE GO BACK AND CONFIRM THE RESPONSES. |  |
| \|  \|  \|  \| reachable_group > over18_group > hiv_positive > c6_6 \| \| --- \| --- \| --- \| --- \| | | |
| \|  \|  \|  \|  \| reachable_group > over18_group > hiv_positive > c6_6 > c6_e1group \| \| --- \| --- \| --- \| --- \| --- \| | | |
| \|  \|  \|  \|  \|  \| c6_e1label \| \| --- \| --- \| --- \| --- \| --- \| --- \| | For the last Diviner visit, how much did you spend for:  Please record the answers in TSh. If DK, enter -98. If RF, enter -99. |  |
| \|  \|  \|  \|  \|  \| c6_e2 (required) \| \| --- \| --- \| --- \| --- \| --- \| --- \| | Clinic/consultation fee |  |
| \|  \|  \|  \|  \|  \| c6_e3 (required) \| \| --- \| --- \| --- \| --- \| --- \| --- \| | Medical tests (e.g. blood tests and X-rays) |  |
| \|  \|  \|  \|  \|  \| c6_e4 (required) \| \| --- \| --- \| --- \| --- \| --- \| --- \| | Medicines |  |
| \|  \|  \|  \|  \|  \| c6_e5 (required) \| \| --- \| --- \| --- \| --- \| --- \| --- \| | Transport |  |
| \|  \|  \|  \|  \|  \| c6_e6 (required) \| \| --- \| --- \| --- \| --- \| --- \| --- \| | Payment for someone to look after your child while you were gone |  |
| \|  \|  \|  \|  \| reachable_group > over18_group > hiv_positive > c6_6 > c6_e7group \| \| --- \| --- \| --- \| --- \| --- \| | | |
| \|  \|  \|  \|  \|  \| c6_e7label \| \| --- \| --- \| --- \| --- \| --- \| --- \| | For the last Diviner visit, how much did you spend for:  Please record the answers in TSh. If DK, enter -98. If RF, enter -99. |  |
| \|  \|  \|  \|  \|  \| c6_e7 (required) \| \| --- \| --- \| --- \| --- \| --- \| --- \| | Payment for someone to look after your house, garden/fields or animals while you were gone |  |
| \|  \|  \|  \|  \|  \| c6_e8 (required) \| \| --- \| --- \| --- \| --- \| --- \| --- \| | Food |  |
| \|  \|  \|  \|  \|  \| c6_e9 (required) \| \| --- \| --- \| --- \| --- \| --- \| --- \| | Phone calls/SMS |  |
| \|  \|  \|  \|  \|  \| c6_e10 (required) \| \| --- \| --- \| --- \| --- \| --- \| --- \| | Accommodation if you needed to stay the night nearby |  |
| \|  \|  \|  \|  \|  \| c6_e11 (required) \| \| --- \| --- \| --- \| --- \| --- \| --- \| | Other (specify below) |  |
| \|  \|  \|  \| c6_e11_other (required) \| \| --- \| --- \| --- \| --- \| | Please specify 'Other' payment related to Diviner visit(s) |  |
| \|  \|  \|  \| c6_f1 (required) \| \| --- \| --- \| --- \| --- \| | In the past SIX MONTHS, how many times did you visit: a faith healer?  If RF, enter -99. |  |
| \|  \|  \|  \| c6_errorf \| \| --- \| --- \| --- \| --- \| | NOTE: YOU INPUT 0 VISITS, BUT IN A PREVIOUS QUESTION YOU INPUT THAT THE RESPONDENT VISITED THIS FACILITY / HEALER IN THE PAST MONTH. PLEASE GO BACK AND CONFIRM THE RESPONSES. |  |
| \|  \|  \|  \| reachable_group > over18_group > hiv_positive > c6_7 \| \| --- \| --- \| --- \| --- \| | | |
| \|  \|  \|  \|  \| reachable_group > over18_group > hiv_positive > c6_7 > c6_f1group \| \| --- \| --- \| --- \| --- \| --- \| | | |
| \|  \|  \|  \|  \|  \| c6_f1label \| \| --- \| --- \| --- \| --- \| --- \| --- \| | For the last Faith healer visit, how much did you spend for:  Please record the answers in TSh. If DK, enter -98. If RF, enter -99. |  |
| \|  \|  \|  \|  \|  \| c6_f2 (required) \| \| --- \| --- \| --- \| --- \| --- \| --- \| | Clinic/consultation fee |  |
| \|  \|  \|  \|  \|  \| c6_f3 (required) \| \| --- \| --- \| --- \| --- \| --- \| --- \| | Medical tests (e.g. blood tests and X-rays) |  |
| \|  \|  \|  \|  \|  \| c6_f4 (required) \| \| --- \| --- \| --- \| --- \| --- \| --- \| | Medicines |  |
| \|  \|  \|  \|  \|  \| c6_f5 (required) \| \| --- \| --- \| --- \| --- \| --- \| --- \| | Transport |  |
| \|  \|  \|  \|  \|  \| c6_f6 (required) \| \| --- \| --- \| --- \| --- \| --- \| --- \| | Payment for someone to look after your child while you were gone |  |
| \|  \|  \|  \|  \| reachable_group > over18_group > hiv_positive > c6_7 > c6_f7group \| \| --- \| --- \| --- \| --- \| --- \| | | |
| \|  \|  \|  \|  \|  \| c6_f7label \| \| --- \| --- \| --- \| --- \| --- \| --- \| | For the last Faith healer visit, how much did you spend for:  Please record the answers in TSh. If DK, enter -98. If RF, enter -99. |  |
| \|  \|  \|  \|  \|  \| c6_f7 (required) \| \| --- \| --- \| --- \| --- \| --- \| --- \| | Payment for someone to look after your house, garden/fields or animals while you were gone |  |
| \|  \|  \|  \|  \|  \| c6_f8 (required) \| \| --- \| --- \| --- \| --- \| --- \| --- \| | Food |  |
| \|  \|  \|  \|  \|  \| c6_f9 (required) \| \| --- \| --- \| --- \| --- \| --- \| --- \| | Phone calls/SMS |  |
| \|  \|  \|  \|  \|  \| c6_f10 (required) \| \| --- \| --- \| --- \| --- \| --- \| --- \| | Accommodation if you needed to stay the night nearby |  |
| \|  \|  \|  \|  \|  \| c6_f11 (required) \| \| --- \| --- \| --- \| --- \| --- \| --- \| | Other (specify below) |  |
| \|  \|  \|  \| c6_f11_other (required) \| \| --- \| --- \| --- \| --- \| | Please specify 'Other' payment related to faith healer visit(s) |  |
| \|  \|  \|  \| c116 (required) \| \| --- \| --- \| --- \| --- \| | How much did you spend on other health care in the past SIX months, such as traditional medicines, medicines from a convenience store, special food, etc.?  Please record the amount in TSh. If DK, enter -98. If RF, enter -99. |  |
| \|  \|  \|  \| c117 (required) \| \| --- \| --- \| --- \| --- \| | In the past six months did you have to borrow money to pay for healthcare? | \|  \| 1 \| Yes \| \| --- \| --- \| --- \| \|  \| 2 \| No \| \|  \| 99 \| REFUSED \| |
| \|  \|  \|  \| c118 (required) \| \| --- \| --- \| --- \| --- \| | How much money did you borrow?  Please record the amount in TSh. If DK, enter -98. If RF, enter -99. |  |
| \|  \|  \|  \| c119 (required) \| \| --- \| --- \| --- \| --- \| | In the past six months did you have to sell personal or household items in order to pay for healthcare? | \|  \| 1 \| Yes \| \| --- \| --- \| --- \| \|  \| 2 \| No \| \|  \| 99 \| REFUSED \| |
| \|  \|  \|  \| c120 \| \| --- \| --- \| --- \| --- \| | At what time did you arrive at the clinic today?  Please enter the time. |  |
| \|  \|  \|  \| reachable_group > over18_group > hiv_positive > time_clinic \| \| --- \| --- \| --- \| --- \| | | |
| \|  \|  \|  \|  \| c121 \| \| --- \| --- \| --- \| --- \| --- \| | How much time did it take you today to get to the clinic?  For example, if it took the respondent 1 hour and 30 minutes to get to the clinic, input '1' HOUR and '30' MINUTES. If respondent does not know, enter '98' HOURS and '98' MINUTES. If respondent refuses, enter '99' HOURS and '99' MINUTES. |  |
| \|  \|  \|  \|  \| c121_hr (required) \| \| --- \| --- \| --- \| --- \| --- \| | HOURS: |  |
| \|  \|  \|  \|  \| c121_min (required) \| \| --- \| --- \| --- \| --- \| --- \| | MINUTES: |  |
| \|  \|  \|  \| c122 (required) \| \| --- \| --- \| --- \| --- \| | Approximately how many minutes did you spend waiting to be seen by a nurse or physician today?  Please enter the number of minutes. If DK, enter -98. If RF, enter -99. |  |
| \|  \|  \|  \| c123 (required) \| \| --- \| --- \| --- \| --- \| | Approximately how many minutes did you spend with the nurse or physician today?  Please enter the number of minutes. If DK, enter -98. If RF, enter -99. |  |
| \|  \|  \|  \| reachable_group > over18_group > hiv_positive > group_c124 \| \| --- \| --- \| --- \| --- \| | | |
| \|  \|  \|  \|  \| c124 (required) \| \| --- \| --- \| --- \| --- \| --- \| | What would you have been doing if you had not gone to the ART clinic today?  Read out each option and select all that apply. | \|  \| 1 \| Earning money \| \| --- \| --- \| --- \| \|  \| 2 \| Doing unpaid community work or volunteer work \| \|  \| 3 \| Doing household chores such as cleaning, cooking, shopping for food, maintenance and repairs, working in the garden, gathering wood, gathering water, housework, etc \| \|  \| 4 \| Taking care of children \| \|  \| 5 \| Leisure activities (sport, watching TV, listening to music, reading, visiting friends and family, going to movies, etc.) \| \|  \| 6 \| Attending school or other educational institution \| \|  \| 7 \| Nothing \| \|  \| 8 \| Other (specify below) \| \|  \| 99 \| REFUSED \| |
| \|  \|  \|  \|  \| c124_other \| \| --- \| --- \| --- \| --- \| --- \| | Specify if 'Other': |  |
| \|  \|  \|  \| c125 (required) \| \| --- \| --- \| --- \| --- \| | Did you lose any money from the time you took to come to the clinic? | \|  \| 1 \| Yes \| \| --- \| --- \| --- \| \|  \| 2 \| No \| \|  \| 99 \| REFUSED \| |
| \|  \|  \|  \| c126 (required) \| \| --- \| --- \| --- \| --- \| | How much money did you lose?  Please record the amount in TSh. If DK, enter -98. If RF, enter -99. |  |
| \|  \|  \|  \| note_d \| \| --- \| --- \| --- \| --- \| | PART 4: COVERAGE OF, AND SATISFACTION WITH HBC SERVICES  I would now like to ask you a few questions about home-based carers. Please bear in mind that all your answers will be treated as highly confidential. No one outside the immediate study team will be told about any answers you gave. |  |
| \|  \|  \|  \| d1 (required) \| \| --- \| --- \| --- \| --- \| | A home-based carer is someone from the community who regularly visits households. Home-based carers provide information on how to stay healthy and help care for ill people at their home.  Have you ever been visited by a home-based carer? | \|  \| 1 \| Yes \| \| --- \| --- \| --- \| \|  \| 2 \| No \| \|  \| 99 \| REFUSED \| |
| \|  \|  \|  \| d2 (required) \| \| --- \| --- \| --- \| --- \| | Has your household ever been visited by a home-based carer? | \|  \| 1 \| Yes \| \| --- \| --- \| --- \| \|  \| 2 \| No \| \|  \| 98 \| Don't know \| \|  \| 99 \| REFUSED \| |
| \|  \|  \|  \| reachable_group > over18_group > hiv_positive > d3_group \| \| --- \| --- \| --- \| --- \| | | |
| \|  \|  \|  \|  \| d3 (required) \| \| --- \| --- \| --- \| --- \| --- \| | In your opinion, why has this household never been visited by a home-based carer?  Read out each option and select all that apply. | \|  \| 1 \| The home-based carer has got too many households to take care of \| \| --- \| --- \| --- \| \|  \| 2 \| We have asked the home-based carer not to visit this household \| \|  \| 3 \| No one in our household has been ill \| \|  \| 4 \| The home-based carer mostly visits wealthy households \| \|  \| 5 \| This community does not have a home-based carer \| \|  \| 6 \| We have personal differences with the home-based carer \| \|  \| 7 \| The home-based carer is too old or sick to do his/her job \| \|  \| 8 \| The home-based carer died \| \|  \| 9 \| The home-based carer only visits his/her friends \| \|  \| 10 \| The home-based carer only visits his/her neighbors \| \|  \| 11 \| The home-based carer is lazy \| \|  \| 98 \| I don't know \| \|  \| 97 \| Other (specify below) \| \|  \| 99 \| REFUSED \| |
| \|  \|  \|  \| d3_other (required) \| \| --- \| --- \| --- \| --- \| | Specify if other reason: |  |
| \|  \|  \|  \| reachable_group > over18_group > hiv_positive > skip_d17 \| \| --- \| --- \| --- \| --- \| | | |
| \|  \|  \|  \|  \| d4 (required) \| \| --- \| --- \| --- \| --- \| --- \| | When was the last time that a home-based carer came to visit your household?  If respondent DK, enter February 1980. If respondent RF, enter March 1980. |  |
| \|  \|  \|  \|  \| d5 (required) \| \| --- \| --- \| --- \| --- \| --- \| | Were you present at the time of the last visit? | \|  \| 1 \| Yes \| \| --- \| --- \| --- \| \|  \| 2 \| No \| \|  \| 99 \| REFUSED \| |
| \|  \|  \|  \|  \| d6 (required) \| \| --- \| --- \| --- \| --- \| --- \| | When was the last time you were present during a visit by a home-based carer?  If respondent DK, enter February 1980. If respondent RF, enter March 1980. |  |
| \|  \|  \|  \|  \| d7 (required) \| \| --- \| --- \| --- \| --- \| --- \| | During the last six months, how many times did a home-based carer visit your household?  If respondent DK, enter "8888." If respondent RF, enter "9999." |  |
| \|  \|  \|  \|  \| reachable_group > over18_group > hiv_positive > skip_d17 > d8_group \| \| --- \| --- \| --- \| --- \| --- \| | | |
| \|  \|  \|  \|  \|  \| d8 (required) \| \| --- \| --- \| --- \| --- \| --- \| --- \| | In your opinion, why does the home-based carer not visit your household more frequently?  Read out each option and select all that apply. | \|  \| 1 \| The home-based carer has got too many households to take care of \| \| --- \| --- \| --- \| \|  \| 2 \| We have asked the home-based carer not to visit this household \| \|  \| 3 \| No one in our household has been ill \| \|  \| 4 \| The home-based carer mostly visits wealthy households \| \|  \| 5 \| This community does not have a home-based carer \| \|  \| 6 \| We have personal differences with the home-based carer \| \|  \| 7 \| The home-based carer is too old or sick to do his/her job \| \|  \| 8 \| The home-based carer died \| \|  \| 9 \| The home-based carer only visits his/her friends \| \|  \| 10 \| The home-based carer only visits his/her neighbors \| \|  \| 11 \| The home-based carer is lazy \| \|  \| 98 \| I don't know \| \|  \| 97 \| Other (specify below) \| \|  \| 99 \| REFUSED \| |
| \|  \|  \|  \|  \| d8_other (required) \| \| --- \| --- \| --- \| --- \| --- \| | Specify if other reason: |  |
| \|  \|  \|  \|  \| d9 (required) \| \| --- \| --- \| --- \| --- \| --- \| | During the last six months, on average, how much time did the home-based carer spend on one visit to your household?  Please enter the number of minutes. If respondent DK, enter "8888." If respondent RF, enter "9999." |  |
| \|  \|  \|  \|  \| d10 (required) \| \| --- \| --- \| --- \| --- \| --- \| | In the last six months, have you always been visited by the same home-based carer? | \|  \| 1 \| Yes \| \| --- \| --- \| --- \| \|  \| 2 \| No \| \|  \| 99 \| REFUSED \| |
| \|  \|  \|  \|  \| d11 (required) \| \| --- \| --- \| --- \| --- \| --- \| | How many different home-based carers have come to visit you in the last six months?  Please enter the number. If respondent DK, enter "8888." If respondent RF, enter "9999." |  |
| \|  \|  \|  \|  \| d12 (required) \| \| --- \| --- \| --- \| --- \| --- \| | Overall, how satisfied or dissatisfied are you with the services provided by the home-based carers in your community?  Ask the respondent to refer to scale 1. Ensure that respondent knows 0=Very dissatisfied, and 10=Very satisfied. | \|  \| 0 \| 0 (Very dissatisfied) \| \| --- \| --- \| --- \| \|  \| 1 \| 1 \| \|  \| 2 \| 2 \| \|  \| 3 \| 3 \| \|  \| 4 \| 4 \| \|  \| 5 \| 5 \| \|  \| 6 \| 6 \| \|  \| 7 \| 7 \| \|  \| 8 \| 8 \| \|  \| 9 \| 9 \| \|  \| 10 \| 10 (Very satisfied) \| \|  \| 99 \| REFUSED \| |
| \|  \|  \|  \|  \| d13 (required) \| \| --- \| --- \| --- \| --- \| --- \| | How satisfied or dissatisfied are you with the accessibility of the home-based carers in your community? With accessibility we mean your ability to see a home-based carer when you are ill or looking for advice.  Ask the respondent to refer to scale 1. Ensure that respondent knows 0=Very dissatisfied, and 10=Very satisfied | \|  \| 0 \| 0 (Very dissatisfied) \| \| --- \| --- \| --- \| \|  \| 1 \| 1 \| \|  \| 2 \| 2 \| \|  \| 3 \| 3 \| \|  \| 4 \| 4 \| \|  \| 5 \| 5 \| \|  \| 6 \| 6 \| \|  \| 7 \| 7 \| \|  \| 8 \| 8 \| \|  \| 9 \| 9 \| \|  \| 10 \| 10 (Very satisfied) \| \|  \| 99 \| REFUSED \| |
| \|  \|  \|  \|  \| d14 (required) \| \| --- \| --- \| --- \| --- \| --- \| | How satisfied or dissatisfied are you with the quality of the advice and care given by the home-based carers in your community?  Ask the respondent to refer to scale 1. Ensure that respondent knows 0=Very dissatisfied, and 10=Very satisfied | \|  \| 0 \| 0 (Very dissatisfied) \| \| --- \| --- \| --- \| \|  \| 1 \| 1 \| \|  \| 2 \| 2 \| \|  \| 3 \| 3 \| \|  \| 4 \| 4 \| \|  \| 5 \| 5 \| \|  \| 6 \| 6 \| \|  \| 7 \| 7 \| \|  \| 8 \| 8 \| \|  \| 9 \| 9 \| \|  \| 10 \| 10 (Very satisfied) \| \|  \| 99 \| REFUSED \| |
| \|  \|  \|  \|  \| d15 (required) \| \| --- \| --- \| --- \| --- \| --- \| | How satisfied or dissatisfied are you with being treated respectfully by home-based carers?  Ask the respondent to refer to scale 1. Ensure that respondent knows 0=Very dissatisfied, and 10=Very satisfied | \|  \| 0 \| 0 (Very dissatisfied) \| \| --- \| --- \| --- \| \|  \| 1 \| 1 \| \|  \| 2 \| 2 \| \|  \| 3 \| 3 \| \|  \| 4 \| 4 \| \|  \| 5 \| 5 \| \|  \| 6 \| 6 \| \|  \| 7 \| 7 \| \|  \| 8 \| 8 \| \|  \| 9 \| 9 \| \|  \| 10 \| 10 (Very satisfied) \| \|  \| 99 \| REFUSED \| |
| \|  \|  \|  \| d16 (required) \| \| --- \| --- \| --- \| --- \| | Do you trust the home-based carers in your area to keep information about your health confidential? With confidential, we mean that the home-based carer does not tell other people about your health without your permission. | \|  \| 1 \| Yes, I trust the home-based carers with my medical information \| \| --- \| --- \| --- \| \|  \| 2 \| No, I don’t trust the home-based carers with my medical information \| \|  \| 3 \| I can’t answer this question because I don’t know who the home-based carers are \| \|  \| 99 \| REFUSED \| |
| \|  \|  \|  \| d17 (required) \| \| --- \| --- \| --- \| --- \| | Would you recommend the home-based carer program to other communities? | \|  \| 1 \| Yes \| \| --- \| --- \| --- \| \|  \| 2 \| No \| \|  \| 99 \| REFUSED \| |
| \|  \|  \|  \| d18 (required) \| \| --- \| --- \| --- \| --- \| | Apart from home-based carers, have any other people come to your house to provide information about health, to offer testing for an illness, or to ask you to come to a healthcare facility? | \|  \| 1 \| Yes \| \| --- \| --- \| --- \| \|  \| 2 \| No \| \|  \| 99 \| REFUSED \| |
| \|  \|  \|  \| d19 (required) \| \| --- \| --- \| --- \| --- \| | Let us refer to this person or these people as “community health workers” in the next few questions. Other than home-based carers, how many community health workers have come to visit you at your home in the last one year?  Please enter a number. If respondent DK, enter "8888." If respondent RF, enter "9999." |  |
| \|  \|  \|  \| d20 (required) \| \| --- \| --- \| --- \| --- \| | During the last one year, how many times did these community health workers come to visit your household?  Please enter a number. If respondent DK, enter "8888." If respondent RF, enter "9999." |  |
| \|  \|  \|  \| d21 (required) \| \| --- \| --- \| --- \| --- \| | IS THIS THE BASELINE QUESTIONNAIRE? | \|  \| 1 \| Yes \| \| --- \| --- \| --- \| \|  \| 2 \| No \| |
| \|  \|  \|  \| d22 (required) \| \| --- \| --- \| --- \| --- \| | The following questions try to find out if patients are interested in community health worker services. But this does NOT influence any care you will receive other than what we have already told you when telling you about this study.  Please think of a community health worker as someone who visits households on a regular basis to provide advice on health issues or to care for those who are ill. Would you like to receive home visits from a community health worker? | \|  \| 1 \| Yes \| \| --- \| --- \| --- \| \|  \| 2 \| No \| \|  \| 99 \| REFUSED \| |
| \|  \|  \|  \| d23 (required) \| \| --- \| --- \| --- \| --- \| | If you had a choice, would you like to receive these home visits by a community health worker once a week, once a month, once every 3 months, or once a year? | \|  \| 1 \| Once a week \| \| --- \| --- \| --- \| \|  \| 2 \| Once a month \| \|  \| 3 \| Once every 3 months \| \|  \| 4 \| Once every 6 months \| \|  \| 99 \| REFUSED \| |
| \|  \|  \|  \| reachable_group > over18_group > hiv_positive > d24_group \| \| --- \| --- \| --- \| --- \| | | |
| \|  \|  \|  \|  \| d24 \| \| --- \| --- \| --- \| --- \| --- \| | If you had the choice, which of the following services would you like to receive from a community health worker who visits your household?  Read out each option and select ‘Yes’ ‘No’ 'Don't know' or 'REFUSED' for each. | \|  \| 1 \| Yes \| \| --- \| --- \| --- \| \|  \| 2 \| No \| \|  \| 98 \| Don't know \| \|  \| 99 \| REFUSED \| |
| \|  \|  \|  \|  \| d24_1 (required) \| \| --- \| --- \| --- \| --- \| --- \| | Information on family planning | \|  \| 1 \| Yes \| \| --- \| --- \| --- \| \|  \| 2 \| No \| \|  \| 98 \| Don't know \| \|  \| 99 \| REFUSED \| |
| \|  \|  \|  \|  \| d24_2 (required) \| \| --- \| --- \| --- \| --- \| --- \| | Providing contraceptives | \|  \| 1 \| Yes \| \| --- \| --- \| --- \| \|  \| 2 \| No \| \|  \| 98 \| Don't know \| \|  \| 99 \| REFUSED \| |
| \|  \|  \|  \|  \| d24_3 (required) \| \| --- \| --- \| --- \| --- \| --- \| | Injections for contraception | \|  \| 1 \| Yes \| \| --- \| --- \| --- \| \|  \| 2 \| No \| \|  \| 98 \| Don't know \| \|  \| 99 \| REFUSED \| |
| \|  \|  \|  \|  \| d24_4 (required) \| \| --- \| --- \| --- \| --- \| --- \| | Providing condoms | \|  \| 1 \| Yes \| \| --- \| --- \| --- \| \|  \| 2 \| No \| \|  \| 98 \| Don't know \| \|  \| 99 \| REFUSED \| |
| \|  \|  \|  \|  \| d24_5 (required) \| \| --- \| --- \| --- \| --- \| --- \| | Providing a pregnancy test | \|  \| 1 \| Yes \| \| --- \| --- \| --- \| \|  \| 2 \| No \| \|  \| 98 \| Don't know \| \|  \| 99 \| REFUSED \| |
| \|  \|  \|  \|  \| d24_6 (required) \| \| --- \| --- \| --- \| --- \| --- \| | Providing medication for HIV | \|  \| 1 \| Yes \| \| --- \| --- \| --- \| \|  \| 2 \| No \| \|  \| 98 \| Don't know \| \|  \| 99 \| REFUSED \| |
| \|  \|  \|  \|  \| d24_7 (required) \| \| --- \| --- \| --- \| --- \| --- \| | Providing medication for tuberculosis | \|  \| 1 \| Yes \| \| --- \| --- \| --- \| \|  \| 2 \| No \| \|  \| 98 \| Don't know \| \|  \| 99 \| REFUSED \| |
| \|  \|  \|  \|  \| d24_8 (required) \| \| --- \| --- \| --- \| --- \| --- \| | Providing other medication | \|  \| 1 \| Yes \| \| --- \| --- \| --- \| \|  \| 2 \| No \| \|  \| 98 \| Don't know \| \|  \| 99 \| REFUSED \| |
| \|  \|  \|  \|  \| d24_9 (required) \| \| --- \| --- \| --- \| --- \| --- \| | HIV-testing | \|  \| 1 \| Yes \| \| --- \| --- \| --- \| \|  \| 2 \| No \| \|  \| 98 \| Don't know \| \|  \| 99 \| REFUSED \| |
| \|  \|  \|  \|  \| d24_10 (required) \| \| --- \| --- \| --- \| --- \| --- \| | Screening for tuberculosis | \|  \| 1 \| Yes \| \| --- \| --- \| --- \| \|  \| 2 \| No \| \|  \| 98 \| Don't know \| \|  \| 99 \| REFUSED \| |
| \|  \|  \|  \| d25 (required) \| \| --- \| --- \| --- \| --- \| | IS THIS A STUDY PARTICIPANT WHO RECEIVES ARVs AT HOME, AND IS THIS THE STUDY EXIT QUESTIONNAIRE? | \|  \| 1 \| Yes \| \| --- \| --- \| --- \| \|  \| 2 \| No \| |
| \|  \|  \|  \| reachable_group > over18_group > hiv_positive > not_exit \| \| --- \| --- \| --- \| --- \| | | |
| \|  \|  \|  \|  \| d26 (required) \| \| --- \| --- \| --- \| --- \| --- \| | How many times in the last six months did a home-based carer come to deliver your HIV medicines to your home?  Please enter the number of visits. If respondent does not know, enter "8888." If respondent refuses, enter "9999." |  |
| \|  \|  \|  \|  \| d27 (required) \| \| --- \| --- \| --- \| --- \| --- \| | Did the home-based carer deliver the HIV medicines on time? Please answer with always, usually, sometimes or never. | \|  \| 1 \| Always \| \| --- \| --- \| --- \| \|  \| 2 \| Usually \| \|  \| 3 \| Sometimes \| \|  \| 4 \| Never \| \|  \| 99 \| REFUSED \| |
| \|  \|  \|  \|  \| d28 (required) \| \| --- \| --- \| --- \| --- \| --- \| | Have you ever had to miss a dose of ARVs because the home-based carer did not deliver the ARVs to your home on time? | \|  \| 1 \| Yes \| \| --- \| --- \| --- \| \|  \| 2 \| No \| \|  \| 99 \| REFUSED \| |
| \|  \|  \|  \|  \| d29 (required) \| \| --- \| --- \| --- \| --- \| --- \| | How many times did this happen?  If respondent DK, enter "8888." If respondent RF, enter "9999." |  |
| \|  \|  \|  \|  \| d30 (required) \| \| --- \| --- \| --- \| --- \| --- \| | If you could choose, would you like to continue receiving HIV medicines brought to your home by a home-based carer, or would you prefer picking them up from the facility yourself? | \|  \| 1 \| Delivered by home-based carer \| \| --- \| --- \| --- \| \|  \| 2 \| Picking up from the facility myself \| \|  \| 98 \| Don't know \| \|  \| 99 \| REFUSED \| |
| \|  \|  \|  \|  \| d31 (required) \| \| --- \| --- \| --- \| --- \| --- \| | Why?  Please read out each option and select all that apply. | \|  \| 1 \| I’m afraid that because of the home-based carers’ visits someone may learn about my HIV status whom I don’t want to know \| \| --- \| --- \| --- \| \|  \| 2 \| The home-based carers are unreliable in delivering the HIV medicines on time \| \|  \| 3 \| I would like to be seen by a physician or a nurse rather than a home-based carer \| \|  \| 4 \| The home-based carers don’t treat me with respect \| \|  \| 5 \| The visits by the home-based carer take up too much of my time \| \|  \| 97 \| Other (SPECIFY ON THE NEXT PAGE) \| \|  \| 99 \| REFUSED \| |
| \|  \|  \|  \|  \| d31other \| \| --- \| --- \| --- \| --- \| --- \| | PLEASE SPECIFY 'Other' REASON. |  |
| \|  \|  \|  \|  \| d32 (required) \| \| --- \| --- \| --- \| --- \| --- \| | Would you recommend this program of home-based carers delivering HIV medicines to people’s homes to other communities? | \|  \| 1 \| Yes \| \| --- \| --- \| --- \| \|  \| 2 \| No \| \|  \| 99 \| REFUSED \| |
| \|  \|  \|  \|  \| reachable_group > over18_group > hiv_positive > not_exit > d33_group \| \| --- \| --- \| --- \| --- \| --- \| | | |
| \|  \|  \|  \|  \|  \| d33_label \| \| --- \| --- \| --- \| --- \| --- \| --- \| | Why not?  Read out each option and select ‘Yes’ ‘No' or 'REFUSED' for each. | \|  \| 1 \| Yes \| \| --- \| --- \| --- \| \|  \| 2 \| No \| \|  \| 99 \| REFUSED \| |
| \|  \|  \|  \|  \|  \| d33_1 (required) \| \| --- \| --- \| --- \| --- \| --- \| --- \| | There is a risk of disclosure of my HIV infection to someone who I don’t want to know about my HIV | \|  \| 1 \| Yes \| \| --- \| --- \| --- \| \|  \| 2 \| No \| \|  \| 99 \| REFUSED \| |
| \|  \|  \|  \|  \|  \| d33_2 (required) \| \| --- \| --- \| --- \| --- \| --- \| --- \| | The home-based carers are unreliable in delivering the HIV medicines on time | \|  \| 1 \| Yes \| \| --- \| --- \| --- \| \|  \| 2 \| No \| \|  \| 99 \| REFUSED \| |
| \|  \|  \|  \|  \|  \| d33_3 (required) \| \| --- \| --- \| --- \| --- \| --- \| --- \| | The home-based carers are not qualified to provide HIV medicines | \|  \| 1 \| Yes \| \| --- \| --- \| --- \| \|  \| 2 \| No \| \|  \| 99 \| REFUSED \| |
| \|  \|  \|  \|  \|  \| d33_4 (required) \| \| --- \| --- \| --- \| --- \| --- \| --- \| | The home-based carers don’t treat me with respect | \|  \| 1 \| Yes \| \| --- \| --- \| --- \| \|  \| 2 \| No \| \|  \| 99 \| REFUSED \| |
| \|  \|  \|  \|  \|  \| d33_5 (required) \| \| --- \| --- \| --- \| --- \| --- \| --- \| | Other (SPECIFY BELOW) | \|  \| 1 \| Yes \| \| --- \| --- \| --- \| \|  \| 2 \| No \| \|  \| 99 \| REFUSED \| |
| \|  \|  \|  \|  \|  \| d33_5other \| \| --- \| --- \| --- \| --- \| --- \| --- \| | Please specify if 'Other' reason: |  |
| \|  \|  \|  \|  \| d34 (required) \| \| --- \| --- \| --- \| --- \| --- \| | Were you ever worried that the delivery of HIV medicines by home-based carers may disclose your HIV status to people who you do not want to know about your HIV infection? | \|  \| 1 \| Yes \| \| --- \| --- \| --- \| \|  \| 2 \| No \| \|  \| 99 \| REFUSED \| |
| \|  \|  \|  \|  \| d35 (required) \| \| --- \| --- \| --- \| --- \| --- \| | Has the home-delivery of HIV medicines actually caused a disclosure of your HIV status to people who you do not want to know about your HIV infections? | \|  \| 1 \| Yes \| \| --- \| --- \| --- \| \|  \| 2 \| No \| \|  \| 99 \| REFUSED \| |
| \|  \|  \|  \|  \| reachable_group > over18_group > hiv_positive > not_exit > d36_group \| \| --- \| --- \| --- \| --- \| --- \| | | |
| \|  \|  \|  \|  \|  \| d36_label \| \| --- \| --- \| --- \| --- \| --- \| --- \| | Are you worried that you may experience any of the following social problems from the home-delivery of HIV medicines by home-based carers?  Read out each option and select ‘Yes’ ‘No' or 'REFUSED' for each. | \|  \| 1 \| Yes \| \| --- \| --- \| --- \| \|  \| 2 \| No \| \|  \| 99 \| REFUSED \| |
| \|  \|  \|  \|  \|  \| d36_1 (required) \| \| --- \| --- \| --- \| --- \| --- \| --- \| | Conflict with spouse or partner | \|  \| 1 \| Yes \| \| --- \| --- \| --- \| \|  \| 2 \| No \| \|  \| 99 \| REFUSED \| |
| \|  \|  \|  \|  \|  \| d36_2 (required) \| \| --- \| --- \| --- \| --- \| --- \| --- \| | Separation or divorce from spouse or partner | \|  \| 1 \| Yes \| \| --- \| --- \| --- \| \|  \| 2 \| No \| \|  \| 99 \| REFUSED \| |
| \|  \|  \|  \|  \|  \| d36_3 (required) \| \| --- \| --- \| --- \| --- \| --- \| --- \| | Abandonment by spouse or partner | \|  \| 1 \| Yes \| \| --- \| --- \| --- \| \|  \| 2 \| No \| \|  \| 99 \| REFUSED \| |
| \|  \|  \|  \|  \|  \| d36_4 (required) \| \| --- \| --- \| --- \| --- \| --- \| --- \| | Beating or other forms of physical violence by spouse or partner | \|  \| 1 \| Yes \| \| --- \| --- \| --- \| \|  \| 2 \| No \| \|  \| 99 \| REFUSED \| |
| \|  \|  \|  \|  \|  \| d36_5 (required) \| \| --- \| --- \| --- \| --- \| --- \| --- \| | Isolation and/or lack of support from family or friends | \|  \| 1 \| Yes \| \| --- \| --- \| --- \| \|  \| 2 \| No \| \|  \| 99 \| REFUSED \| |
| \|  \|  \|  \|  \|  \| d36_6 (required) \| \| --- \| --- \| --- \| --- \| --- \| --- \| | Being a burden or source of worry for others | \|  \| 1 \| Yes \| \| --- \| --- \| --- \| \|  \| 2 \| No \| \|  \| 99 \| REFUSED \| |
| \|  \|  \|  \|  \|  \| d36_7 (required) \| \| --- \| --- \| --- \| --- \| --- \| --- \| | Teasing or insulting | \|  \| 1 \| Yes \| \| --- \| --- \| --- \| \|  \| 2 \| No \| \|  \| 99 \| REFUSED \| |
| \|  \|  \|  \|  \|  \| d36_8 (required) \| \| --- \| --- \| --- \| --- \| --- \| --- \| | Loss of respect or standing with the family and/or community | \|  \| 1 \| Yes \| \| --- \| --- \| --- \| \|  \| 2 \| No \| \|  \| 99 \| REFUSED \| |
| \|  \|  \|  \|  \|  \| d36_9 (required) \| \| --- \| --- \| --- \| --- \| --- \| --- \| | Loss of customers | \|  \| 1 \| Yes \| \| --- \| --- \| --- \| \|  \| 2 \| No \| \|  \| 99 \| REFUSED \| |
| \|  \|  \|  \|  \|  \| d36_10 (required) \| \| --- \| --- \| --- \| --- \| --- \| --- \| | Loss of a job | \|  \| 1 \| Yes \| \| --- \| --- \| --- \| \|  \| 2 \| No \| \|  \| 99 \| REFUSED \| |
| \|  \|  \|  \|  \|  \| d36_11 (required) \| \| --- \| --- \| --- \| --- \| --- \| --- \| | Taking away of property | \|  \| 1 \| Yes \| \| --- \| --- \| --- \| \|  \| 2 \| No \| \|  \| 99 \| REFUSED \| |
| \|  \|  \|  \|  \|  \| d36_12 (required) \| \| --- \| --- \| --- \| --- \| --- \| --- \| | Taking away of a child | \|  \| 1 \| Yes \| \| --- \| --- \| --- \| \|  \| 2 \| No \| \|  \| 99 \| REFUSED \| |
| \|  \|  \|  \|  \| d37 (required) \| \| --- \| --- \| --- \| --- \| --- \| | Have you actually experienced any of these social problems?  Select all that apply. Probe with: any others? | \|  \| 1 \| Conflict with spouse or partner \| \| --- \| --- \| --- \| \|  \| 2 \| Separation or divorce from spouse or partner \| \|  \| 3 \| Abandonment by spouse or partner \| \|  \| 4 \| Beating or other forms of physical violence by spouse or partner \| \|  \| 5 \| Isolation and/or lack of support from family or friends \| \|  \| 6 \| Being a burden or source of worry for others \| \|  \| 7 \| Teasing or insulting \| \|  \| 8 \| Loss of respect or standing with the family and/or community \| \|  \| 9 \| Loss of customers \| \|  \| 10 \| Loss of a job \| \|  \| 11 \| Taking away of property \| \|  \| 12 \| Taking away of a child \| \|  \| 99 \| REFUSED \| |
| \|  \|  \|  \| e_intro \| \| --- \| --- \| --- \| --- \| | PART 5: PATIENT SATISFACTION  The following section asks you about your experience today at the clinic. For these questions, you are asked to rate your experience with very good, good, moderate, bad or very bad.  Ask the patient to refer to scale 2. |  |
| \|  \|  \|  \| e1 (required) \| \| --- \| --- \| --- \| --- \| | Please think about your visit to the clinic today beginning from the time you arrived up until now.  How would you rate your experience with the service you received today?  1=Very good  2=Good  3=Moderate  4=Bad  5=Very bad | \|  \| 1 \| 1 \| \| --- \| --- \| --- \| \|  \| 2 \| 2 \| \|  \| 3 \| 3 \| \|  \| 4 \| 4 \| \|  \| 5 \| 5 \| \|  \| 99 \| REFUSED \| |
| \|  \|  \|  \| e2 (required) \| \| --- \| --- \| --- \| --- \| | Would you recommend your friend or relative to attend this facility for HIV care? | \|  \| 1 \| Yes \| \| --- \| --- \| --- \| \|  \| 2 \| No \| \|  \| 99 \| REFUSED \| |
| \|  \|  \|  \| e3_a (required) \| \| --- \| --- \| --- \| --- \| | Overall, how would you rate the amount of time you waited before being attended to?  1=Very good  2=Good  3=Moderate  4=Bad  5=Very bad | \|  \| 1 \| 1 \| \| --- \| --- \| --- \| \|  \| 2 \| 2 \| \|  \| 3 \| 3 \| \|  \| 4 \| 4 \| \|  \| 5 \| 5 \| \|  \| 99 \| REFUSED \| |
| \|  \|  \|  \| e3_b (required) \| \| --- \| --- \| --- \| --- \| | John always attends his visits to the HIV clinic. Each time he visits the clinic he has to wait about 30 minutes before he can see the nurse or doctor.  Overall, how would you rate the amount of time John waited before being attended to?  1=Very good  2=Good  3=Moderate  4=Bad  5=Very bad | \|  \| 1 \| 1 \| \| --- \| --- \| --- \| \|  \| 2 \| 2 \| \|  \| 3 \| 3 \| \|  \| 4 \| 4 \| \|  \| 5 \| 5 \| \|  \| 99 \| REFUSED \| |
| \|  \|  \|  \| e3_c (required) \| \| --- \| --- \| --- \| --- \| | Neema always attends her visits to the HIV clinic. The clinic is always busy. Each time she visits the clinic she usually has to wait about 1-2 hours before she can see the nurse or doctor. On occasion, she has waited for the whole day and then had to leave without seeing a doctor.  Overall, how would you rate the amount of time Neema waited before being attended to?  1=Very good  2=Good  3=Moderate  4=Bad  5=Very bad | \|  \| 1 \| 1 \| \| --- \| --- \| --- \| \|  \| 2 \| 2 \| \|  \| 3 \| 3 \| \|  \| 4 \| 4 \| \|  \| 5 \| 5 \| \|  \| 99 \| REFUSED \| |
| \|  \|  \|  \| e4_a (required) \| \| --- \| --- \| --- \| --- \| | Overall, how would you rate your experience of getting involved as much as you wanted to be in making decisions about your care or treatment?  1=Very good  2=Good  3=Moderate  4=Bad  5=Very bad | \|  \| 1 \| 1 \| \| --- \| --- \| --- \| \|  \| 2 \| 2 \| \|  \| 3 \| 3 \| \|  \| 4 \| 4 \| \|  \| 5 \| 5 \| \|  \| 99 \| REFUSED \| |
| \|  \|  \|  \| e4_b (required) \| \| --- \| --- \| --- \| --- \| | Mary attends the ART clinic regularly. She has discussed her preference to change one of her ARVs. Her doctor adjusted the prescriptions to take account of her preference. She was also pregnant and concerned about infecting her baby with HIV during delivery. The doctor discussed different delivery options with her and helped her prepare a birth plan.  Overall, how would you rate Mary’s experience of getting involved as much as she wanted to be in making decisions about her care or treatment?  1=Very good  2=Good  3=Moderate  4=Bad  5=Very bad | \|  \| 1 \| 1 \| \| --- \| --- \| --- \| \|  \| 2 \| 2 \| \|  \| 3 \| 3 \| \|  \| 4 \| 4 \| \|  \| 5 \| 5 \| \|  \| 99 \| REFUSED \| |
| \|  \|  \|  \| e4_c (required) \| \| --- \| --- \| --- \| --- \| | Mohamed attends the ART clinic regularly. On this visit, he complains to the doctor that he wasn’t reacting well to his ARVs and would like them changed. The doctor reassures him but still prescribes the same ARVs. He also asks him to perform some tests without explaining why or asking for his permission. He only tells him that the tests are important.  Overall, how would you rate Mohamed’s experience of getting involved as much as he wanted to be in making decisions about his care or treatment?  1=Very good  2=Good  3=Moderate  4=Bad  5=Very bad | \|  \| 1 \| 1 \| \| --- \| --- \| --- \| \|  \| 2 \| 2 \| \|  \| 3 \| 3 \| \|  \| 4 \| 4 \| \|  \| 5 \| 5 \| \|  \| 99 \| REFUSED \| |
| \|  \|  \|  \| e5_a (required) \| \| --- \| --- \| --- \| --- \| | Overall, how would you rate the experience of how much time you spent seeing the health provider?  1=Very good  2=Good  3=Moderate  4=Bad  5=Very bad | \|  \| 1 \| 1 \| \| --- \| --- \| --- \| \|  \| 2 \| 2 \| \|  \| 3 \| 3 \| \|  \| 4 \| 4 \| \|  \| 5 \| 5 \| \|  \| 99 \| REFUSED \| |
| \|  \|  \|  \| e5_b (required) \| \| --- \| --- \| --- \| --- \| | Lawrence goes to the clinic for his regular HIV care visit. When it gets to his turn to see the nurse, the nurse spends over 20 minutes talking with him. The nurse asks him some questions, listens to his answers, and also answers all the questions Lawrence asks.  Overall, how would you rate the experience of how much time [name] spent seeing the health provider?  1=Very good  2=Good  3=Moderate  4=Bad  5=Very bad | \|  \| 1 \| 1 \| \| --- \| --- \| --- \| \|  \| 2 \| 2 \| \|  \| 3 \| 3 \| \|  \| 4 \| 4 \| \|  \| 5 \| 5 \| \|  \| 99 \| REFUSED \| |
| \|  \|  \|  \| e5_c (required) \| \| --- \| --- \| --- \| --- \| | Farida goes to the clinic for her regular HIV care visit. When it gets to her turn, the nurse asks her a few questions without looking up, writes down some notes and dismisses her in less than 10 minutes.  Overall, how would you rate the experience of how much time Farida spent seeing the health provider?  1=Very good  2=Good  3=Moderate  4=Bad  5=Very bad | \|  \| 1 \| 1 \| \| --- \| --- \| --- \| \|  \| 2 \| 2 \| \|  \| 3 \| 3 \| \|  \| 4 \| 4 \| \|  \| 5 \| 5 \| \|  \| 99 \| REFUSED \| |
| \|  \|  \|  \| e6_a (required) \| \| --- \| --- \| --- \| --- \| | Overall, how would you rate your experience of being greeted and talked to respectfully?  1=Very good  2=Good  3=Moderate  4=Bad  5=Very bad | \|  \| 1 \| 1 \| \| --- \| --- \| --- \| \|  \| 2 \| 2 \| \|  \| 3 \| 3 \| \|  \| 4 \| 4 \| \|  \| 5 \| 5 \| \|  \| 99 \| REFUSED \| |
| \|  \|  \|  \| e6_b (required) \| \| --- \| --- \| --- \| --- \| | Nuru attends the ART clinic for the first time. She didn't know how the clinic worked so she spoke to the first person she saw in nurse uniform. The person greeted her and escorted her to the room where she could receive HIV care. When she got there, she was greeted by a nurse and asked what she wanted. Throughout the discussion, the nurse gave Nuru her full attention.  Overall, how would you rate Nuru’s experience of being greeted and talked to respectfully?  1=Very good  2=Good  3=Moderate  4=Bad  5=Very bad | \|  \| 1 \| 1 \| \| --- \| --- \| --- \| \|  \| 2 \| 2 \| \|  \| 3 \| 3 \| \|  \| 4 \| 4 \| \|  \| 5 \| 5 \| \|  \| 99 \| REFUSED \| |
| \|  \|  \|  \| e6_c (required) \| \| --- \| --- \| --- \| --- \| | Joseph attends the ART clinic for the first time. He didn't know how the clinic worked so he spoke to the first person he saw in nurse uniform who told him: "go sit there and wait, we will call you". After a few minutes, the nurse pointed to him and said "come". Without looking at him, the nurse made some notes, and kept on talking to another nurse about something that had happened to a friend of hers.  Overall, how would you rate Joseph’s experience of being greeted and talked to respectfully? Please circle a number.  1=Very good  2=Good  3=Moderate  4=Bad  5=Very bad | \|  \| 1 \| 1 \| \| --- \| --- \| --- \| \|  \| 2 \| 2 \| \|  \| 3 \| 3 \| \|  \| 4 \| 4 \| \|  \| 5 \| 5 \| \|  \| 99 \| REFUSED \| |
| \|  \|  \|  \| reachable_group > over18_group > hiv_positive > f1-6_group \| \| --- \| --- \| --- \| --- \| | | |
| \|  \|  \|  \|  \| f_note \| \| --- \| --- \| --- \| --- \| --- \| | PART 6: HIV KNOWLEDGE AND ATTITUDE  Now, I would like to talk with you a bit more about HIV or AIDS. | \|  \| 1 \| Yes \| \| --- \| --- \| --- \| \|  \| 2 \| No \| \|  \| 99 \| REFUSED \| |
| \|  \|  \|  \|  \| f1 (required) \| \| --- \| --- \| --- \| --- \| --- \| | Can people reduce their chance of getting the AIDS virus by having just one uninfected sex partner who has no other sex partners? | \|  \| 1 \| Yes \| \| --- \| --- \| --- \| \|  \| 2 \| No \| \|  \| 99 \| REFUSED \| |
| \|  \|  \|  \|  \| f2 (required) \| \| --- \| --- \| --- \| --- \| --- \| | Can people get the AIDS virus because of witchcraft or other supernatural means? | \|  \| 1 \| Yes \| \| --- \| --- \| --- \| \|  \| 2 \| No \| \|  \| 99 \| REFUSED \| |
| \|  \|  \|  \|  \| f3 (required) \| \| --- \| --- \| --- \| --- \| --- \| | Can people reduce their chance of getting the AIDS virus by using a condom every time they have sex? | \|  \| 1 \| Yes \| \| --- \| --- \| --- \| \|  \| 2 \| No \| \|  \| 99 \| REFUSED \| |
| \|  \|  \|  \|  \| f4 (required) \| \| --- \| --- \| --- \| --- \| --- \| | Can people get the AIDS virus from mosquito bites? | \|  \| 1 \| Yes \| \| --- \| --- \| --- \| \|  \| 2 \| No \| \|  \| 99 \| REFUSED \| |
| \|  \|  \|  \|  \| f5 (required) \| \| --- \| --- \| --- \| --- \| --- \| | Can people get the AIDS virus by sharing food with a person who has the AIDS virus? | \|  \| 1 \| Yes \| \| --- \| --- \| --- \| \|  \| 2 \| No \| \|  \| 99 \| REFUSED \| |
| \|  \|  \|  \|  \| f6 (required) \| \| --- \| --- \| --- \| --- \| --- \| | Is it possible for a healthy-looking person to have the AIDS virus? | \|  \| 1 \| Yes \| \| --- \| --- \| --- \| \|  \| 2 \| No \| \|  \| 99 \| REFUSED \| |
| \|  \|  \|  \| reachable_group > over18_group > hiv_positive > f7_group \| \| --- \| --- \| --- \| --- \| | | |
| \|  \|  \|  \|  \| f7_note \| \| --- \| --- \| --- \| --- \| --- \| | Can the virus that causes AIDS be transmitted from a mother to her baby: | \|  \| 1 \| Yes \| \| --- \| --- \| --- \| \|  \| 2 \| No \| \|  \| 98 \| Don't know \| \|  \| 99 \| REFUSED \| |
| \|  \|  \|  \|  \| f7_1 (required) \| \| --- \| --- \| --- \| --- \| --- \| | During pregnancy? | \|  \| 1 \| Yes \| \| --- \| --- \| --- \| \|  \| 2 \| No \| \|  \| 98 \| Don't know \| \|  \| 99 \| REFUSED \| |
| \|  \|  \|  \|  \| f7_2 (required) \| \| --- \| --- \| --- \| --- \| --- \| | During delivery? | \|  \| 1 \| Yes \| \| --- \| --- \| --- \| \|  \| 2 \| No \| \|  \| 98 \| Don't know \| \|  \| 99 \| REFUSED \| |
| \|  \|  \|  \|  \| f7_3 (required) \| \| --- \| --- \| --- \| --- \| --- \| | By breastfeeding? | \|  \| 1 \| Yes \| \| --- \| --- \| --- \| \|  \| 2 \| No \| \|  \| 98 \| Don't know \| \|  \| 99 \| REFUSED \| |
| \|  \|  \|  \| reachable_group > over18_group > hiv_positive > f8-10_group \| \| --- \| --- \| --- \| --- \| | | |
| \|  \|  \|  \|  \| f8 (required) \| \| --- \| --- \| --- \| --- \| --- \| | In your opinion, if a female teacher has the AIDS virus but is not sick, should she be allowed to continue teaching in school? | \|  \| 1 \| Yes \| \| --- \| --- \| --- \| \|  \| 2 \| No \| \|  \| 98 \| Don’t know/Not sure/Depends \| \|  \| 99 \| REFUSED \| |
| \|  \|  \|  \|  \| f9 (required) \| \| --- \| --- \| --- \| --- \| --- \| | If another member of your family got infected with the AIDS virus, would you want it to remain a secret? | \|  \| 1 \| Yes \| \| --- \| --- \| --- \| \|  \| 2 \| No \| \|  \| 98 \| Don’t know/Not sure/Depends \| \|  \| 99 \| REFUSED \| |
| \|  \|  \|  \|  \| f10 (required) \| \| --- \| --- \| --- \| --- \| --- \| | Do you think children living with HIV should be allowed  to attend school with children who do not have HIV? | \|  \| 1 \| Yes \| \| --- \| --- \| --- \| \|  \| 2 \| No \| \|  \| 98 \| Don’t know/Not sure/Depends \| \|  \| 99 \| REFUSED \| |
| \|  \|  \|  \| g_note \| \| --- \| --- \| --- \| --- \| | PART 7. FAMILY PLANNING |  |
| \|  \|  \|  \| g1 (required) \| \| --- \| --- \| --- \| --- \| | Do you have any children? | \|  \| 1 \| Yes \| \| --- \| --- \| --- \| \|  \| 2 \| No \| \|  \| 99 \| REFUSED \| |
| \|  \|  \|  \| g2 (required) \| \| --- \| --- \| --- \| --- \| | How many children do you have?  If RF, enter 9999. |  |
| \|  \|  \|  \| g3 (required) \| \| --- \| --- \| --- \| --- \| | Now I have some questions about the future. Would you like to have or another child, or would you prefer not to have any more children? | \|  \| 1 \| Have (a/another) child \| \| --- \| --- \| --- \| \|  \| 2 \| No more/none \| \|  \| 3 \| She/partner/wife can’t get pregnant \| \|  \| 98 \| Undecided/don’t know \| \|  \| 99 \| REFUSED \| |
| \|  \|  \|  \| g4 (required) \| \| --- \| --- \| --- \| --- \| | Are you or your partner currently doing something or using any method to delay or avoid getting pregnant? | \|  \| 1 \| Yes \| \| --- \| --- \| --- \| \|  \| 2 \| No \| \|  \| 99 \| REFUSED \| \|  \| 97 \| Not currently sexually active \| |
| \|  \|  \|  \| reachable_group > over18_group > hiv_positive > g5_group \| \| --- \| --- \| --- \| --- \| | | |
| \|  \|  \|  \|  \| g5 (required) \| \| --- \| --- \| --- \| --- \| --- \| | Which method are you using?  Select all methods mentioned. Probe with: any others? | \|  \| 1 \| Female sterilization \| \| --- \| --- \| --- \| \|  \| 2 \| Male sterilization \| \|  \| 3 \| IUD \| \|  \| 4 \| Injectables \| \|  \| 5 \| Implants \| \|  \| 6 \| Pill \| \|  \| 7 \| Condom \| \|  \| 8 \| Female condom \| \|  \| 9 \| Emergency contraception \| \|  \| 10 \| Standard days method \| \|  \| 11 \| Lactational amenorrhea method \| \|  \| 12 \| Rhythm method \| \|  \| 13 \| Withdrawal \| \|  \| 97 \| Other method (specify below) \| \|  \| 99 \| REFUSED \| |
| \|  \|  \|  \|  \| g5_other \| \| --- \| --- \| --- \| --- \| --- \| | Please specify 'Other' method: |  |
| \|  \|  \|  \| reachable_group > over18_group > hiv_positive > g6_group \| \| --- \| --- \| --- \| --- \| | | |
| \|  \|  \|  \|  \| g6 (required) \| \| --- \| --- \| --- \| --- \| --- \| | Where did you obtain (CURRENT METHODS) the last time? | \|  \| 1 \| Public sector: Government hospital \| \| --- \| --- \| --- \| \|  \| 2 \| Public sector: Government health center \| \|  \| 3 \| Public sector: Family planning clinic \| \|  \| 4 \| Public sector: Mobile clinic \| \|  \| 5 \| Public sector: Home-based carer \| \|  \| 6 \| Other community health worker (SPECIFY BELOW) \| \|  \| 8 \| Other public sector (SPECIFY BELOW) \| \|  \| 9 \| Private sector: private hospital/clinic \| \|  \| 10 \| Private sector: Pharmacy \| \|  \| 11 \| Private sector: Private doctor \| \|  \| 12 \| Private sector: Mobile clinic \| \|  \| 13 \| Other private medical sector (SPECIFY BELOW) \| \|  \| 14 \| Shop \| \|  \| 15 \| Church \| \|  \| 16 \| Friends/relative \| \|  \| 97 \| Unable to determine if public or private sector (SPECIFY BELOW) \| \|  \| 98 \| DON'T KNOW \| \|  \| 99 \| REFUSED \| |
| \|  \|  \|  \|  \| g6_text \| \| --- \| --- \| --- \| --- \| --- \| | If unable to determine if public or private sector, write the name of the place. If you selected an "Other" option, please specify here. |  |
| \|  \|  \|  \| g7 (required) \| \| --- \| --- \| --- \| --- \| | Have you ever used anything or tried in any way to delay or avoid getting pregnant? | \|  \| 1 \| Yes \| \| --- \| --- \| --- \| \|  \| 2 \| No \| \|  \| 99 \| REFUSED \| |
| \|  \|  \|  \| g8 (required) \| \| --- \| --- \| --- \| --- \| | Where did you get it at that time? | \|  \| 1 \| Public sector: Government hospital \| \| --- \| --- \| --- \| \|  \| 2 \| Public sector: Government health center \| \|  \| 3 \| Public sector: Family planning clinic \| \|  \| 4 \| Public sector: Mobile clinic \| \|  \| 5 \| Public sector: Home-based carer \| \|  \| 6 \| Other community health worker (SPECIFY BELOW) \| \|  \| 8 \| Other public sector (SPECIFY BELOW) \| \|  \| 9 \| Private sector: private hospital/clinic \| \|  \| 10 \| Private sector: Pharmacy \| \|  \| 11 \| Private sector: Private doctor \| \|  \| 12 \| Private sector: Mobile clinic \| \|  \| 13 \| Other private medical sector (SPECIFY BELOW) \| \|  \| 14 \| Shop \| \|  \| 15 \| Church \| \|  \| 16 \| Friends/relative \| \|  \| 97 \| Unable to determine if public or private sector (SPECIFY BELOW) \| \|  \| 98 \| DON'T KNOW \| \|  \| 99 \| REFUSED \| |
| \|  \|  \|  \| g8_text (required) \| \| --- \| --- \| --- \| --- \| | If unable to determine if public or private sector, write the name of the place. If you selected an "Other" option, please specify here. |  |
| \|  \|  \|  \| g9 (required) \| \| --- \| --- \| --- \| --- \| | Do you know of a place where you can obtain a method of family planning? | \|  \| 1 \| Yes \| \| --- \| --- \| --- \| \|  \| 2 \| No \| \|  \| 99 \| REFUSED \| |
| \|  \|  \|  \| g10-11_note \| \| --- \| --- \| --- \| --- \| | I will now read you some statements about contraception. Please tell me if you agree or disagree with each one. |  |
| \|  \|  \|  \| g10 (required) \| \| --- \| --- \| --- \| --- \| | Contraception is a woman’s concern and a man should not have to worry about it. | \|  \| 1 \| Agree \| \| --- \| --- \| --- \| \|  \| 2 \| Disagree \| \|  \| 98 \| Don't know \| \|  \| 99 \| REFUSED \| |
| \|  \|  \|  \| g11 (required) \| \| --- \| --- \| --- \| --- \| | Women who use contraception may become promiscuous. | \|  \| 1 \| Agree \| \| --- \| --- \| --- \| \|  \| 2 \| Disagree \| \|  \| 98 \| Don't know \| \|  \| 99 \| REFUSED \| |
| \|  \|  \|  \| hB_title \| \| --- \| --- \| --- \| --- \| | PART 8: NUTRITION-RELATED QUESTIONS  PART 8A: ANTHROPOMETRY |  |
| \|  \|  \|  \| reachable_group > over18_group > hiv_positive > - \| \| --- \| --- \| --- \| --- \| | | |
| \|  \|  \|  \|  \| hA1 (required) \| \| --- \| --- \| --- \| --- \| --- \| | 8.A1 Weigh the individual. Record the weight in kg: |  |
| \|  \|  \|  \|  \| hA2 (required) \| \| --- \| --- \| --- \| --- \| --- \| | 8.A2 Measure the height of the individual. Record the height in cm: |  |
| \|  \|  \|  \|  \| hA3 (required) \| \| --- \| --- \| --- \| --- \| --- \| | 8.A3 Measure the mid upper arm circumference (MUAC) of the individual. Record the MUAC in cm: |  |
| \|  \|  \|  \| hB_note \| \| --- \| --- \| --- \| --- \| | PART 8B: DIETARY INTAKE ASSESSMENT  Please read out each option PLUS examples, and circle yes or no. |  |
| \|  \|  \|  \| hB1-16_note \| \| --- \| --- \| --- \| --- \| | In the past 24 hours have you eaten any of the following foods?  Please read out each option PLUS examples, and circle yes or no. |  |
| \|  \|  \|  \| hB1 \| \| --- \| --- \| --- \| --- \| | Cereals  Examples: corn/maize, rice, wheat, sorghum, millet or any other grains or foods made from these (e.g. bread, noodles, porridge or other grain products) + insert local foods e.g. ugali, nshima, porridge or paste | \|  \| 1 \| Yes \| \| --- \| --- \| --- \| \|  \| 2 \| No \| \|  \| 99 \| REFUSED \| |
| \|  \|  \|  \| hB2 (required) \| \| --- \| --- \| --- \| --- \| | White roots and tubers  Examples: white potatoes, white yam, white cassava, or other foods made from roots | \|  \| 1 \| Yes \| \| --- \| --- \| --- \| \|  \| 2 \| No \| \|  \| 99 \| REFUSED \| |
| \|  \|  \|  \| hB3 (required) \| \| --- \| --- \| --- \| --- \| | Vitamin A rich vegetables and tubers  Examples: pumpkin, carrot, squash, or sweet potato that are orange inside + other locally available vitamin A rich vegetables (e.g. red sweet pepper) | \|  \| 1 \| Yes \| \| --- \| --- \| --- \| \|  \| 2 \| No \| \|  \| 99 \| REFUSED \| |
| \|  \|  \|  \| hB4 (required) \| \| --- \| --- \| --- \| --- \| | Dark green leafy vegetables  Examples: dark green leafy vegetables, including wild forms + locally available vitamin A rich leaves such as amaranth, cassava leaves,  kale, spinach | \|  \| 1 \| Yes \| \| --- \| --- \| --- \| \|  \| 2 \| No \| \|  \| 99 \| REFUSED \| |
| \|  \|  \|  \| hB5 (required) \| \| --- \| --- \| --- \| --- \| | Other vegetables  Examples: other vegetables (e.g. tomato, onion, eggplant) + other locally available vegetables | \|  \| 1 \| Yes \| \| --- \| --- \| --- \| \|  \| 2 \| No \| \|  \| 99 \| REFUSED \| |
| \|  \|  \|  \| hB6 (required) \| \| --- \| --- \| --- \| --- \| | Vitamin A rich fruits  Examples: ripe mango, cantaloupe, apricot (fresh or dried), ripe papaya, dried peach, and 100% fruit juice made from these + other locally available vitamin A rich fruits | \|  \| 1 \| Yes \| \| --- \| --- \| --- \| \|  \| 2 \| No \| \|  \| 99 \| REFUSED \| |
| \|  \|  \|  \| hB7 (required) \| \| --- \| --- \| --- \| --- \| | Other fruits  Examples: other fruits, including wild fruits and 100% fruit juice made from these | \|  \| 1 \| Yes \| \| --- \| --- \| --- \| \|  \| 2 \| No \| \|  \| 99 \| REFUSED \| |
| \|  \|  \|  \| hB8 (required) \| \| --- \| --- \| --- \| --- \| | Organ meat  Examples: liver, kidney, heart or other organ meats or blood-based foods | \|  \| 1 \| Yes \| \| --- \| --- \| --- \| \|  \| 2 \| No \| \|  \| 99 \| REFUSED \| |
| \|  \|  \|  \| hB9 (required) \| \| --- \| --- \| --- \| --- \| | Flesh meats  Examples: beef, pork, lamb, goat, rabbit, game, chicken, duck, other birds, insects | \|  \| 1 \| Yes \| \| --- \| --- \| --- \| \|  \| 2 \| No \| \|  \| 99 \| REFUSED \| |
| \|  \|  \|  \| hB10 (required) \| \| --- \| --- \| --- \| --- \| | Eggs  Examples: eggs from chicken, duck, guinea fowl or any other egg | \|  \| 1 \| Yes \| \| --- \| --- \| --- \| \|  \| 2 \| No \| \|  \| 99 \| REFUSED \| |
| \|  \|  \|  \| hB11 (required) \| \| --- \| --- \| --- \| --- \| | Fish and seafood  Examples: fresh or dried fish or shellfish | \|  \| 1 \| Yes \| \| --- \| --- \| --- \| \|  \| 2 \| No \| \|  \| 99 \| REFUSED \| |
| \|  \|  \|  \| hB12 (required) \| \| --- \| --- \| --- \| --- \| | Legumes, nuts and seeds  Examples: dried beans, dried peas, lentils, nuts, seeds or foods made from these (eg. hummus, peanut butter) | \|  \| 1 \| Yes \| \| --- \| --- \| --- \| \|  \| 2 \| No \| \|  \| 99 \| REFUSED \| |
| \|  \|  \|  \| hB13 (required) \| \| --- \| --- \| --- \| --- \| | Milk and milk products  Examples: milk, cheese, yogurt or other milk products | \|  \| 1 \| Yes \| \| --- \| --- \| --- \| \|  \| 2 \| No \| \|  \| 99 \| REFUSED \| |
| \|  \|  \|  \| hB14 (required) \| \| --- \| --- \| --- \| --- \| | Oils and fats  Examples: oil, fats or butter added to food or used for cooking | \|  \| 1 \| Yes \| \| --- \| --- \| --- \| \|  \| 2 \| No \| \|  \| 99 \| REFUSED \| |
| \|  \|  \|  \| hB15 (required) \| \| --- \| --- \| --- \| --- \| | Sweets  Examples: sugar, honey, sweetened soda or sweetened juice drinks, sugary foods such as chocolates, candies, cookies and cakes | \|  \| 1 \| Yes \| \| --- \| --- \| --- \| \|  \| 2 \| No \| \|  \| 99 \| REFUSED \| |
| \|  \|  \|  \| hB16 (required) \| \| --- \| --- \| --- \| --- \| | Spices, Condiments, Beverages  Examples: spices (black pepper, salt), condiments (soy sauce, hot sauce), coffee, tea, alcoholic beverages | \|  \| 1 \| Yes \| \| --- \| --- \| --- \| \|  \| 2 \| No \| \|  \| 99 \| REFUSED \| |
| \|  \|  \|  \| alcohol_consent (required) \| \| --- \| --- \| --- \| --- \| | We will now ask you three questions about your alcohol intake. Do you agree to answer them? | \|  \| 1 \| Yes \| \| --- \| --- \| --- \| \|  \| 2 \| No \| |
| \|  \|  \|  \| reachable_group > over18_group > hiv_positive > group_alcohol \| \| --- \| --- \| --- \| --- \| | | |
| \|  \|  \|  \|  \| hB138 (required) \| \| --- \| --- \| --- \| --- \| --- \| | 8.B138: How often do you have a drink containing alcohol? | \|  \| 0 \| Never \| \| --- \| --- \| --- \| \|  \| 1 \| Monthly or less \| \|  \| 2 \| 2-4 times a month \| \|  \| 3 \| 2-3 times a week \| \|  \| 4 \| 4 or more times a week \| |
| \|  \|  \|  \|  \| hB139 (required) \| \| --- \| --- \| --- \| --- \| --- \| | 8.B139: How many standard drinks do you have on a typical day when drinking?  (Use the Standard alcohol card provided). | \|  \| 0 \| 1 or 2 \| \| --- \| --- \| --- \| \|  \| 1 \| 3 or 4 \| \|  \| 2 \| 5 or 6 \| \|  \| 3 \| 7 or 9 \| \|  \| 4 \| 10 or more \| |
| \|  \|  \|  \|  \| hB140 (required) \| \| --- \| --- \| --- \| --- \| --- \| | 8.B140: How often do you have six or more drinks on one occasion?  (Use the Standard alcohol card provided). | \|  \| 0 \| Never \| \| --- \| --- \| --- \| \|  \| 1 \| Less than monthly \| \|  \| 2 \| Monthly \| \|  \| 3 \| Weekly \| \|  \| 4 \| Daily or almost daily \| |
| \|  \|  \|  \|  \| hB140_note_noalc \| \| --- \| --- \| --- \| --- \| --- \| | The total alcohol score for this respondent is 0.  If the total alcohol score is 5 or more the client has risky alcohol intake and should be counseled and encouraged to reduce alcohol consumption on the non-risky alcohol use using the WHO guidelines. |  |
| \|  \|  \|  \|  \| hB140_note \| \| --- \| --- \| --- \| --- \| --- \| | The total alcohol score for this respondent is [alcohol_score].  If the total alcohol score is 5 or more the client has risky alcohol intake and should be counseled and encouraged to reduce alcohol consumption on the non-risky alcohol use using the WHO guidelines. |  |
| \|  \|  \|  \| hC_note \| \| --- \| --- \| --- \| --- \| | PART 8C. QUESTIONS ASSESSING FOOD SECURITY, CURRENT FOOD SOURCES, CURRENT STATUS OF OWN-PRODUCTION OF NUTRITIOUS FOODS, AND WILLINGNESS TO PRODUCE FOOD FOR OWN-CONSUMPTION |  |
| \|  \|  \|  \| reachable_group > over18_group > hiv_positive > hC1-3_group \| \| --- \| --- \| --- \| --- \| | | |
| \|  \|  \|  \|  \| hC1 (required) \| \| --- \| --- \| --- \| --- \| --- \| | In the past four weeks, did you worry that your household would not have enough food? | \|  \| 1 \| Never \| \| --- \| --- \| --- \| \|  \| 2 \| Rarely (once or twice in the past four weeks) \| \|  \| 3 \| Sometimes (three to ten times in the past four weeks) \| \|  \| 4 \| Often (more than ten times in the past four weeks) \| \|  \| 99 \| REFUSED \| |
| \|  \|  \|  \|  \| hC2 (required) \| \| --- \| --- \| --- \| --- \| --- \| | In the past four weeks, were you or any household member not able to eat the kinds of foods you preferred because of a lack of resources? | \|  \| 1 \| Never \| \| --- \| --- \| --- \| \|  \| 2 \| Rarely (once or twice in the past four weeks) \| \|  \| 3 \| Sometimes (three to ten times in the past four weeks) \| \|  \| 4 \| Often (more than ten times in the past four weeks) \| \|  \| 99 \| REFUSED \| |
| \|  \|  \|  \|  \| hC3 (required) \| \| --- \| --- \| --- \| --- \| --- \| | In the past four weeks, did you or any household member have to eat a limited variety of foods due to a lack of resources? | \|  \| 1 \| Never \| \| --- \| --- \| --- \| \|  \| 2 \| Rarely (once or twice in the past four weeks) \| \|  \| 3 \| Sometimes (three to ten times in the past four weeks) \| \|  \| 4 \| Often (more than ten times in the past four weeks) \| \|  \| 99 \| REFUSED \| |
| \|  \|  \|  \| reachable_group > over18_group > hiv_positive > hC4-6_group \| \| --- \| --- \| --- \| --- \| | | |
| \|  \|  \|  \|  \| hC4 (required) \| \| --- \| --- \| --- \| --- \| --- \| | In the past four weeks, did you or any household member have to eat some foods that you really did not want to eat because of a lack of resources to obtain other types of food? | \|  \| 1 \| Never \| \| --- \| --- \| --- \| \|  \| 2 \| Rarely (once or twice in the past four weeks) \| \|  \| 3 \| Sometimes (three to ten times in the past four weeks) \| \|  \| 4 \| Often (more than ten times in the past four weeks) \| \|  \| 99 \| REFUSED \| |
| \|  \|  \|  \|  \| hC5 (required) \| \| --- \| --- \| --- \| --- \| --- \| | In the past four weeks, did you or any household member have to eat a smaller meal than you felt you needed because there was not enough food? | \|  \| 1 \| Never \| \| --- \| --- \| --- \| \|  \| 2 \| Rarely (once or twice in the past four weeks) \| \|  \| 3 \| Sometimes (three to ten times in the past four weeks) \| \|  \| 4 \| Often (more than ten times in the past four weeks) \| \|  \| 99 \| REFUSED \| |
| \|  \|  \|  \|  \| hC6 (required) \| \| --- \| --- \| --- \| --- \| --- \| | In the past four weeks, did you or any household member have to eat fewer meals in a day because there was not enough food? | \|  \| 1 \| Never \| \| --- \| --- \| --- \| \|  \| 2 \| Rarely (once or twice in the past four weeks) \| \|  \| 3 \| Sometimes (three to ten times in the past four weeks) \| \|  \| 4 \| Often (more than ten times in the past four weeks) \| \|  \| 99 \| REFUSED \| |
| \|  \|  \|  \| reachable_group > over18_group > hiv_positive > hC7-9_group \| \| --- \| --- \| --- \| --- \| | | |
| \|  \|  \|  \|  \| hC7 (required) \| \| --- \| --- \| --- \| --- \| --- \| | In the past four weeks, was there ever no food to eat of any kind in your household because of lack of resources to get food? | \|  \| 1 \| Never \| \| --- \| --- \| --- \| \|  \| 2 \| Rarely (once or twice in the past four weeks) \| \|  \| 3 \| Sometimes (three to ten times in the past four weeks) \| \|  \| 4 \| Often (more than ten times in the past four weeks) \| \|  \| 99 \| REFUSED \| |
| \|  \|  \|  \|  \| hC8 (required) \| \| --- \| --- \| --- \| --- \| --- \| | In the past four weeks, did you or any household member go to sleep at night hungry because there was not enough food? | \|  \| 1 \| Never \| \| --- \| --- \| --- \| \|  \| 2 \| Rarely (once or twice in the past four weeks) \| \|  \| 3 \| Sometimes (three to ten times in the past four weeks) \| \|  \| 4 \| Often (more than ten times in the past four weeks) \| \|  \| 99 \| REFUSED \| |
| \|  \|  \|  \|  \| hC9 (required) \| \| --- \| --- \| --- \| --- \| --- \| | In the past four weeks, did you or any household member go a whole day and night without eating anything because there was not enough food? | \|  \| 1 \| Never \| \| --- \| --- \| --- \| \|  \| 2 \| Rarely (once or twice in the past four weeks) \| \|  \| 3 \| Sometimes (three to ten times in the past four weeks) \| \|  \| 4 \| Often (more than ten times in the past four weeks) \| \|  \| 99 \| REFUSED \| |
| \|  \|  \|  \| hC10 (required) \| \| --- \| --- \| --- \| --- \| | Could you please tell me the primary source for obtaining grain foods or cereals like rice, millet, or maize for your household to consume? | \|  \| 1 \| Own production, gathering, hunting, fishing \| \| --- \| --- \| --- \| \|  \| 2 \| Purchased \| \|  \| 3 \| Borrowed, bartered, exchanged for labor, gift from friends or relatives \| \|  \| 4 \| Food aid \| \|  \| 5 \| Other \| \|  \| 6 \| We do not have a means of obtaining this food \| \|  \| 98 \| Don't know \| \|  \| 99 \| REFUSED \| |
| \|  \|  \|  \| hC11 (required) \| \| --- \| --- \| --- \| --- \| | Could you please tell me the primary source for obtaining tubers and roots like potatoes, cassava or taro for your household to consume? | \|  \| 1 \| Own production, gathering, hunting, fishing \| \| --- \| --- \| --- \| \|  \| 2 \| Purchased \| \|  \| 3 \| Borrowed, bartered, exchanged for labor, gift from friends or relatives \| \|  \| 4 \| Food aid \| \|  \| 5 \| Other \| \|  \| 6 \| We do not have a means of obtaining this food \| \|  \| 98 \| Don't know \| \|  \| 99 \| REFUSED \| |
| \|  \|  \|  \| hC12 (required) \| \| --- \| --- \| --- \| --- \| | Could you please tell me the primary source for obtaining legumes like beans, lentils, or groundnuts for your household to consume? | \|  \| 1 \| Own production, gathering, hunting, fishing \| \| --- \| --- \| --- \| \|  \| 2 \| Purchased \| \|  \| 3 \| Borrowed, bartered, exchanged for labor, gift from friends or relatives \| \|  \| 4 \| Food aid \| \|  \| 5 \| Other \| \|  \| 6 \| We do not have a means of obtaining this food \| \|  \| 98 \| Don't know \| \|  \| 99 \| REFUSED \| |
| \|  \|  \|  \| hC13 (required) \| \| --- \| --- \| --- \| --- \| | Could you please tell me the primary source for obtaining fruits and vegetables for your household to consume? | \|  \| 1 \| Own production, gathering, hunting, fishing \| \| --- \| --- \| --- \| \|  \| 2 \| Purchased \| \|  \| 3 \| Borrowed, bartered, exchanged for labor, gift from friends or relatives \| \|  \| 4 \| Food aid \| \|  \| 5 \| Other \| \|  \| 6 \| We do not have a means of obtaining this food \| \|  \| 98 \| Don't know \| \|  \| 99 \| REFUSED \| |
| \|  \|  \|  \| hC14 (required) \| \| --- \| --- \| --- \| --- \| | Could you please tell me the primary source for obtaining meat, poultry or fish for your household to consume? | \|  \| 1 \| Own production, gathering, hunting, fishing \| \| --- \| --- \| --- \| \|  \| 2 \| Purchased \| \|  \| 3 \| Borrowed, bartered, exchanged for labor, gift from friends or relatives \| \|  \| 4 \| Food aid \| \|  \| 5 \| Other \| \|  \| 6 \| We do not have a means of obtaining this food \| \|  \| 98 \| Don't know \| \|  \| 99 \| REFUSED \| |
| \|  \|  \|  \| hC15 (required) \| \| --- \| --- \| --- \| --- \| | Could you please tell me the primary source for obtaining eggs, milk and other dairy products for your household to consume? | \|  \| 1 \| Own production, gathering, hunting, fishing \| \| --- \| --- \| --- \| \|  \| 2 \| Purchased \| \|  \| 3 \| Borrowed, bartered, exchanged for labor, gift from friends or relatives \| \|  \| 4 \| Food aid \| \|  \| 5 \| Other \| \|  \| 6 \| We do not have a means of obtaining this food \| \|  \| 98 \| Don't know \| \|  \| 99 \| REFUSED \| |
| \|  \|  \|  \| hC16 (required) \| \| --- \| --- \| --- \| --- \| | Does any member of this household own any agricultural land? | \|  \| 1 \| Yes \| \| --- \| --- \| --- \| \|  \| 2 \| No \| \|  \| 98 \| Don't know \| \|  \| 99 \| REFUSED \| |
| \|  \|  \|  \| hC17_1 (required) \| \| --- \| --- \| --- \| --- \| | Do members of this household own more than 1 acre of land? | \|  \| 1 \| Yes \| \| --- \| --- \| --- \| \|  \| 2 \| No \| \|  \| 99 \| REFUSED \| |
| \|  \|  \|  \| hC17_2 (required) \| \| --- \| --- \| --- \| --- \| | How many square meters of agricultural land do members of this household own?  If DK enter '999.8', and if RF enter '999.9' |  |
| \|  \|  \|  \| hC17_3 (required) \| \| --- \| --- \| --- \| --- \| | We will now ask about the amount of land that members of this household own.  Do you prefer to answer in acres or hectares? | \|  \| 1 \| Acres \| \| --- \| --- \| --- \| \|  \| 2 \| Hectares \| |
| \|  \|  \|  \| hC17_4 (required) \| \| --- \| --- \| --- \| --- \| | How many acres of agricultural land do members of this household own?  If DK enter '998.0', if RF enter '999.0' and if respondent answers >1000 acres then enter 999.9 |  |
| \|  \|  \|  \| hC17_5 (required) \| \| --- \| --- \| --- \| --- \| | How many hectares of agricultural land do members of this household own?  If DK enter '998.0', if RF enter '999.0' and if respondent answers >400 hectares then enter 999.9 |  |
| \|  \|  \|  \| hC18 (required) \| \| --- \| --- \| --- \| --- \| | Do you have access to a garden or a plot of land where you could grow vegetables or fruits? | \|  \| 1 \| Yes \| \| --- \| --- \| --- \| \|  \| 2 \| No \| \|  \| 99 \| REFUSED \| |
| \|  \|  \|  \| hC19_1 (required) \| \| --- \| --- \| --- \| --- \| | Is this garden or plot of land more than 1 acre? | \|  \| 1 \| Yes \| \| --- \| --- \| --- \| \|  \| 2 \| No \| \|  \| 99 \| REFUSED \| |
| \|  \|  \|  \| hC19_2 (required) \| \| --- \| --- \| --- \| --- \| | How many square meters is this garden or plot of land?  If DK enter '999.8', and if RF enter '999.9' |  |
| \|  \|  \|  \| hC19_3 (required) \| \| --- \| --- \| --- \| --- \| | We will now ask about the garden or plot of land where you grow fruits or vegetables.  Do you prefer to answer in acres or hectares? | \|  \| 1 \| Acres \| \| --- \| --- \| --- \| \|  \| 2 \| Hectares \| |
| \|  \|  \|  \| hC19_4 (required) \| \| --- \| --- \| --- \| --- \| | How many acres is the garden or plot of land?  If DK enter '998.0', if RF enter '999.0' and if respondent answers >1000 acres then enter 999.9 |  |
| \|  \|  \|  \| hC19_5 (required) \| \| --- \| --- \| --- \| --- \| | How many hectares is the garden or plot of land?  If DK enter '998.0', if RF enter '999.0' and if respondent answers >400 hectares then enter 999.9 |  |
| \|  \|  \|  \| reachable_group > over18_group > hiv_positive > hC20 \| \| --- \| --- \| --- \| --- \| | | |
| \|  \|  \|  \|  \| hC20_note \| \| --- \| --- \| --- \| --- \| --- \| | In the last 12 months, did you grow any of the following crops: | \|  \| 1 \| Yes \| \| --- \| --- \| --- \| \|  \| 2 \| No \| |
| \|  \|  \|  \|  \| hC20_1 (required) \| \| --- \| --- \| --- \| --- \| --- \| | Cereals (e.g. maize, sorghum, millet, rice, amaranth grain, etc.)? | \|  \| 1 \| Yes \| \| --- \| --- \| --- \| \|  \| 2 \| No \| |
| \|  \|  \|  \|  \| hC20_2 (required) \| \| --- \| --- \| --- \| --- \| --- \| | Bananas or plantains? | \|  \| 1 \| Yes \| \| --- \| --- \| --- \| \|  \| 2 \| No \| |
| \|  \|  \|  \|  \| hC20_3 (required) \| \| --- \| --- \| --- \| --- \| --- \| | Tubers or roots (e.g. cassava, Irish potato, sweet potato, yam, etc.)? | \|  \| 1 \| Yes \| \| --- \| --- \| --- \| \|  \| 2 \| No \| |
| \|  \|  \|  \|  \| hC20_4 (required) \| \| --- \| --- \| --- \| --- \| --- \| | Legumes (e.g. beans, soybeans, cowpea, groundnuts, etc.)? | \|  \| 1 \| Yes \| \| --- \| --- \| --- \| \|  \| 2 \| No \| |
| \|  \|  \|  \|  \| hC20_5 (required) \| \| --- \| --- \| --- \| --- \| --- \| | Vegetables (e.g. tomato, pepper, pumpkin, carrot, onion, okra, cabbage, dark green leafy vegetables, etc.)? | \|  \| 1 \| Yes \| \| --- \| --- \| --- \| \|  \| 2 \| No \| |
| \|  \|  \|  \|  \| hC20_6 (required) \| \| --- \| --- \| --- \| --- \| --- \| | Fruits (e.g. mangoes, pawpaw, avocado, oranges, pineapple, watermelon, etc.)? | \|  \| 1 \| Yes \| \| --- \| --- \| --- \| \|  \| 2 \| No \| |
| \|  \|  \|  \| hC21 (required) \| \| --- \| --- \| --- \| --- \| | On a scale from 0 to 4, with 0 meaning you are very unwilling and 4 meaning you are very willing, how willing are you to grow your own food, fruits and vegetables for your household to consume? | \|  \| 0 \| 0 (Very unwilling) \| \| --- \| --- \| --- \| \|  \| 1 \| 1 (Somewhat willing) \| \|  \| 2 \| 2 (Neutral) \| \|  \| 3 \| 3 (Somewhat willing) \| \|  \| 4 \| 4 (Very willing) \| \|  \| 98 \| DON'T KNOW \| \|  \| 99 \| REFUSED \| |
| \|  \|  \|  \| hC22 (required) \| \| --- \| --- \| --- \| --- \| | Does this household own any livestock, herds, other farm animals, or poultry? | \|  \| 1 \| Yes \| \| --- \| --- \| --- \| \|  \| 2 \| No \| \|  \| 99 \| REFUSED \| |
| \|  \|  \|  \| hC23 (required) \| \| --- \| --- \| --- \| --- \| | On a scale from 0 to 4, with 0 meaning you are very unwilling and 4 meaning you are very willing, how willing are you to raise livestock or poultry for your household to consume? | \|  \| 0 \| 0 (Very unwilling) \| \| --- \| --- \| --- \| \|  \| 1 \| 1 (Somewhat willing) \| \|  \| 2 \| 2 (Neutral) \| \|  \| 3 \| 3 (Somewhat willing) \| \|  \| 4 \| 4 (Very willing) \| \|  \| 98 \| DON'T KNOW \| \|  \| 99 \| REFUSED \| |
| \|  \|  \|  \| hC24 (required) \| \| --- \| --- \| --- \| --- \| | In the last six months, did you grow any vegetables or fruits in a sac, bag, or bucket? | \|  \| 1 \| Yes \| \| --- \| --- \| --- \| \|  \| 2 \| No \| \|  \| 99 \| REFUSED \| |
| \|  \|  \|  \| reachable_group > over18_group > hiv_positive > hC25_group \| \| --- \| --- \| --- \| --- \| | | |
| \|  \|  \|  \|  \| hC25 \| \| --- \| --- \| --- \| --- \| --- \| | In the last six months, did you grow any of the following in a sac, bag, or bucket? | \|  \| 1 \| Yes \| \| --- \| --- \| --- \| \|  \| 2 \| No \| |
| \|  \|  \|  \|  \| hC25_1 (required) \| \| --- \| --- \| --- \| --- \| --- \| | Cereals (e.g. maize, sorghum, millet, rice, amaranth grain, etc.)? | \|  \| 1 \| Yes \| \| --- \| --- \| --- \| \|  \| 2 \| No \| |
| \|  \|  \|  \|  \| hC25_2 (required) \| \| --- \| --- \| --- \| --- \| --- \| | Bananas or plantains? | \|  \| 1 \| Yes \| \| --- \| --- \| --- \| \|  \| 2 \| No \| |
| \|  \|  \|  \|  \| hC25_3 (required) \| \| --- \| --- \| --- \| --- \| --- \| | Tubers or roots (e.g. cassava, Irish potato, sweet potato, yam, etc.)? | \|  \| 1 \| Yes \| \| --- \| --- \| --- \| \|  \| 2 \| No \| |
| \|  \|  \|  \|  \| hC25_4 (required) \| \| --- \| --- \| --- \| --- \| --- \| | Legumes (e.g. beans, soybeans, cowpea, groundnuts, etc.)? | \|  \| 1 \| Yes \| \| --- \| --- \| --- \| \|  \| 2 \| No \| |
| \|  \|  \|  \|  \| hC25_5 (required) \| \| --- \| --- \| --- \| --- \| --- \| | Vegetables (e.g. tomato, pepper, pumpkin, carrot, onion, okra, cabbage, dark green leafy vegetables, etc.)? | \|  \| 1 \| Yes \| \| --- \| --- \| --- \| \|  \| 2 \| No \| |
| \|  \|  \|  \|  \| hC25_6 (required) \| \| --- \| --- \| --- \| --- \| --- \| | Fruits (e.g. mangoes, pawpaw, avocado, oranges, pineapple, watermelon, etc.)? | \|  \| 1 \| Yes \| \| --- \| --- \| --- \| \|  \| 2 \| No \| |
| \|  \|  \|  \| hC26 (required) \| \| --- \| --- \| --- \| --- \| | On a scale from 0 to 4, with 0 meaning you are very unwilling and 4 meaning you are very willing, how willing are you to grow fruits or vegetables in a sac, bag or bucket for your own consumption? | \|  \| 0 \| 0 (Very unwilling) \| \| --- \| --- \| --- \| \|  \| 1 \| 1 (Somewhat willing) \| \|  \| 2 \| 2 (Neutral) \| \|  \| 3 \| 3 (Somewhat willing) \| \|  \| 4 \| 4 (Very willing) \| \|  \| 98 \| DON'T KNOW \| \|  \| 99 \| REFUSED \| |
| \|  \|  \|  \| hC27 (required) \| \| --- \| --- \| --- \| --- \| | Have you contacted an agricultural extension worker in the last 12 months? | \|  \| 1 \| Yes \| \| --- \| --- \| --- \| \|  \| 2 \| No \| \|  \| 99 \| REFUSED \| |
| \|  \|  \|  \| hC28 (required) \| \| --- \| --- \| --- \| --- \| | Have you used any services from an agricultural extension worker in the last 12 months? | \|  \| 1 \| Yes \| \| --- \| --- \| --- \| \|  \| 2 \| No \| \|  \| 99 \| REFUSED \| |
| \|  \|  \|  \| hD_note \| \| --- \| --- \| --- \| --- \| | PART 8D: PHYSICAL ACTIVITY  Now I am going to ask you about the time you spend doing different types of physical activity in a typical week. Please answer these questions even if you do not consider yourself to be a physically active person. Think first about the time you spend doing work. Think of work as the things that you have to do such as paid or unpaid work, study/training, household chores, farming, harvesting food/crops, fishing or hunting for food, or seeking employment. In answering the following questions 'vigorous-intensity activities' are activities that require hard physical effort and cause large increases in breathing or heart rate, 'moderate-intensity activities' are activities that require moderate physical effort and cause small increases in breathing or heart rate. |  |
| \|  \|  \|  \| hD1 (required) \| \| --- \| --- \| --- \| --- \| | Does your work involve vigorous-intensity activity that causes large increases in breathing or heart rate like carrying or lifting heavy loads, digging or construction work, for at least 10 minutes continuously?  (Use showcard) | \|  \| 1 \| Yes \| \| --- \| --- \| --- \| \|  \| 2 \| No \| \|  \| 99 \| REFUSED \| |
| \|  \|  \|  \| hD2 (required) \| \| --- \| --- \| --- \| --- \| | In a typical week, on how many days do you do vigorous-intensity activities as part of your work?  Enter '998' if respondent does not know. Enter '999' if respondent refuses. |  |
| \|  \|  \|  \| reachable_group > over18_group > hiv_positive > hD3 \| \| --- \| --- \| --- \| --- \| | | |
| \|  \|  \|  \|  \| hD3_note \| \| --- \| --- \| --- \| --- \| --- \| | How much time do you spend doing vigorous-intensity activities at work on a typical day?  For example, if the respondent spends 1 hour and 30 minutes, input '1' HOUR and '30' MINUTES. If respondent does not know, enter '98' HOURS and '98' MINUTES. If respondent refuses, enter '99' HOURS and '99' MINUTES. |  |
| \|  \|  \|  \|  \| hD3_hours (required) \| \| --- \| --- \| --- \| --- \| --- \| | HOURS: |  |
| \|  \|  \|  \|  \| hD3_minutes (required) \| \| --- \| --- \| --- \| --- \| --- \| | MINUTES: |  |
| \|  \|  \|  \| hD4 (required) \| \| --- \| --- \| --- \| --- \| | Does your work involve moderate-intensity activity, that causes small increases in breathing or heart rate such as brisk walking or carrying light loads for at least 10 minutes continuously?  (Use showcard). | \|  \| 1 \| Yes \| \| --- \| --- \| --- \| \|  \| 2 \| No \| \|  \| 99 \| REFUSED \| |
| \|  \|  \|  \| hD5 (required) \| \| --- \| --- \| --- \| --- \| | In a typical week, on how many days do you do moderate-intensity activities as part of your work?  Enter '998' if respondent does not know. Enter '999' if respondent refuses. |  |
| \|  \|  \|  \| reachable_group > over18_group > hiv_positive > hD6 \| \| --- \| --- \| --- \| --- \| | | |
| \|  \|  \|  \|  \| hD6_note \| \| --- \| --- \| --- \| --- \| --- \| | How much time do you spend doing moderate-intensity activities at work on a typical day?  For example, if the respondent spends 1 hour and 30 minutes, input '1' HOUR and '30' MINUTES. If respondent does not know, enter '98' HOURS and '98' MINUTES. If respondent refuses, enter '99' HOURS and '99' MINUTES. |  |
| \|  \|  \|  \|  \| hD6_hours (required) \| \| --- \| --- \| --- \| --- \| --- \| | HOURS: |  |
| \|  \|  \|  \|  \| hD6_minutes (required) \| \| --- \| --- \| --- \| --- \| --- \| | MINUTES: |  |
| \|  \|  \|  \| hD_note3 \| \| --- \| --- \| --- \| --- \| | The next questions exclude the physical activities at work that you have already mentioned.  Now I would like to ask you about the usual way you travel to and from places. For example to work, for shopping, to market, to place of worship. |  |
| \|  \|  \|  \| hD7 (required) \| \| --- \| --- \| --- \| --- \| | Do you walk or use a bicycle for at least 10 minutes continuously to get to and from places? | \|  \| 1 \| Yes \| \| --- \| --- \| --- \| \|  \| 2 \| No \| \|  \| 99 \| REFUSED \| |
| \|  \|  \|  \| hD8 (required) \| \| --- \| --- \| --- \| --- \| | In a typical week, on how many days do you walk or bicycle for at least 10 minutes continuously to get to and from places?  Enter '998' if respondent does not know. Enter '999' if respondent refuses. |  |
| \|  \|  \|  \| reachable_group > over18_group > hiv_positive > hD9_group \| \| --- \| --- \| --- \| --- \| | | |
| \|  \|  \|  \|  \| hD9 \| \| --- \| --- \| --- \| --- \| --- \| | How much time do you spend walking or bicycling for travel on a typical day?  For example, if the respondent spends 1 hour and 30 minutes, input '1' HOUR and '30' MINUTES. If respondent does not know, enter '98' HOURS and '98' MINUTES. If respondent refuses, enter '99' HOURS and '99' MINUTES. |  |
| \|  \|  \|  \|  \| hD9_hours (required) \| \| --- \| --- \| --- \| --- \| --- \| | HOURS: |  |
| \|  \|  \|  \|  \| hD9_minutes (required) \| \| --- \| --- \| --- \| --- \| --- \| | MINUTES: |  |
| \|  \|  \|  \| hD_note4 \| \| --- \| --- \| --- \| --- \| | The next questions exclude the work and transport activities that you have already mentioned.  Now I would like to ask you about sports, fitness, leisure and recreational activities. |  |
| \|  \|  \|  \| hD10 (required) \| \| --- \| --- \| --- \| --- \| | Do you do any vigorous-intensity sports, fitness or recreational or leisure activities that cause large increases in breathing or heart rate, like running or football, for at least 10 minutes continuously?  (Use showcard). | \|  \| 1 \| Yes \| \| --- \| --- \| --- \| \|  \| 2 \| No \| \|  \| 99 \| REFUSED \| |
| \|  \|  \|  \| hD11 (required) \| \| --- \| --- \| --- \| --- \| | In a typical week, on how many days do you do vigorous-intensity sports, fitness or recreational activities?  Enter '998' if respondent does not knew. Enter '999' if respondent refuses. |  |
| \|  \|  \|  \| reachable_group > over18_group > hiv_positive > hD12_group \| \| --- \| --- \| --- \| --- \| | | |
| \|  \|  \|  \|  \| hD12 \| \| --- \| --- \| --- \| --- \| --- \| | How much time do you spend doing vigorous-intensity sports, fitness or recreational activities on a typical day?  For example, if the respondent spends 1 hour and 30 minutes, input '1' HOUR and '30' MINUTES. If respondent does not know, enter '98' HOURS and '98' MINUTES. If respondent refuses, enter '99' HOURS and '99' MINUTES. |  |
| \|  \|  \|  \|  \| hD12_hours (required) \| \| --- \| --- \| --- \| --- \| --- \| | HOURS: |  |
| \|  \|  \|  \|  \| hD12_minutes (required) \| \| --- \| --- \| --- \| --- \| --- \| | MINUTES: |  |
| \|  \|  \|  \| hD13 (required) \| \| --- \| --- \| --- \| --- \| | Do you do any moderate-intensity sports, fitness or recreational or leisure activities that cause a small increase in breathing or heart rate such as brisk walking, cycling, swimming, volleyball, for at least 10 minutes continuously?  (Use showcard). | \|  \| 1 \| Yes \| \| --- \| --- \| --- \| \|  \| 2 \| No \| \|  \| 99 \| REFUSED \| |
| \|  \|  \|  \| hD14 (required) \| \| --- \| --- \| --- \| --- \| | In a typical week, on how many days do you do moderate-intensity sports, fitness or recreational activities? |  |
| \|  \|  \|  \| reachable_group > over18_group > hiv_positive > hD15_group \| \| --- \| --- \| --- \| --- \| | | |
| \|  \|  \|  \|  \| hD15 \| \| --- \| --- \| --- \| --- \| --- \| | How much time do you spend doing moderate-intensity sports, fitness or recreational activities on a typical day?  For example, if the respondent spends 1 hour and 30 minutes, input '1' HOUR and '30' MINUTES. If respondent does not know, enter '98' HOURS and '98' MINUTES. If respondent refuses, enter '99' HOURS and '99' MINUTES. |  |
| \|  \|  \|  \|  \| hD15_hours (required) \| \| --- \| --- \| --- \| --- \| --- \| | HOURS: |  |
| \|  \|  \|  \|  \| hD15_minutes (required) \| \| --- \| --- \| --- \| --- \| --- \| | MINUTES: |  |
| \|  \|  \|  \| reachable_group > over18_group > hiv_positive > hD16_group \| \| --- \| --- \| --- \| --- \| | | |
| \|  \|  \|  \|  \| hD16_note \| \| --- \| --- \| --- \| --- \| --- \| | The following question is about sitting or reclining at work, at home, getting to and from places, or with friends including time spent sitting at a desk, sitting with friends, traveling in a car or bus, reading, playing cards or watching television, but do not include time spent sleeping. |  |
| \|  \|  \|  \|  \| hD16 \| \| --- \| --- \| --- \| --- \| --- \| | How much time do you usually spend sitting or reclining on a typical day?  For example, if the respondent spends 1 hour and 30 minutes, input '1' HOUR and '30' MINUTES. If respondent does not know, enter '98' HOURS and '98' MINUTES. If respondent refuses, enter '99' HOURS and '99' MINUTES. |  |
| \|  \|  \|  \|  \| hD16_hours (required) \| \| --- \| --- \| --- \| --- \| --- \| | HOURS: |  |
| \|  \|  \|  \|  \| hD16_minutes (required) \| \| --- \| --- \| --- \| --- \| --- \| | MINUTES: |  |
| \|  \|  \|  \| time_end \| \| --- \| --- \| --- \| --- \| | Time at end of interview |  |
| \|  \|  \|  \| respondent_comments \| \| --- \| --- \| --- \| --- \| | Thank you very much for your effort and time!  Do you have any comments or feedback for us? |  |
| \|  \|  \|  \| seeds_accept (required) \| \| --- \| --- \| --- \| --- \| | This patient is eligible to receive seeds. Did the patient accept the seeds? | \|  \| 1 \| Yes \| \| --- \| --- \| --- \| \|  \| 2 \| No \| |
| \|  \|  \|  \| seeds_whynot (required) \| \| --- \| --- \| --- \| --- \| | Why did the patient not accept the seeds? | \|  \| 1 \| Patient is already growing something else in the garden/land \| \| --- \| --- \| --- \| \|  \| 2 \| Patient does not have time for growing vegetables \| \|  \| 97 \| Other reason \| |
| \|  \|  \|  \| seeds_specify_whynot (required) \| \| --- \| --- \| --- \| --- \| | Specify other reason for patient not accepting the seeds. |  |
| \|  \|  \|  \| reachable_group > over18_group > hiv_positive > eligibility \| \| --- \| --- \| --- \| --- \| | | |
| \|  \|  \|  \|  \| aa1 (required) \| \| --- \| --- \| --- \| --- \| --- \| | Eligibility  Patient CTC2: [ctc2]  Patient Study ID: [rspid_final]  Has this participant been receiving ART for at least 6 months? | \|  \| 1 \| Yes \| \| --- \| --- \| --- \| \|  \| 2 \| No \| |
| \|  \|  \|  \|  \| aa2 (required) \| \| --- \| --- \| --- \| --- \| --- \| | Does this patient have a viral load taken in the last 12 months? | \|  \| 1 \| Yes \| \| --- \| --- \| --- \| \|  \| 2 \| No \| |
| \|  \|  \|  \|  \| aa3 (required) \| \| --- \| --- \| --- \| --- \| --- \| | Is the viral load suppressed?  A viral load is suppressed if it is less than 1,000 copies/ml. | \|  \| 1 \| Yes \| \| --- \| --- \| --- \| \|  \| 2 \| No \| |
| \|  \|  \|  \|  \| aa4 (required) \| \| --- \| --- \| --- \| --- \| --- \| | Had the participant been receiving ART for at least 6 months when the most current viral load was measured? | \|  \| 1 \| Yes \| \| --- \| --- \| --- \| \|  \| 2 \| No \| |
| \|  \|  \|  \|  \| aa5 (required) \| \| --- \| --- \| --- \| --- \| --- \| | Does this patient have a CD4-count taken in the last 12 months? | \|  \| 1 \| Yes \| \| --- \| --- \| --- \| \|  \| 2 \| No \| |
| \|  \|  \|  \|  \| aa6 (required) \| \| --- \| --- \| --- \| --- \| --- \| | Is the most current CD4-count greater than 350? | \|  \| 1 \| Yes \| \| --- \| --- \| --- \| \|  \| 2 \| No \| |
| \|  \|  \|  \|  \| aa7 (required) \| \| --- \| --- \| --- \| --- \| --- \| | Had the participant been receiving ART for at least 6 months when the most current CD4-count was measured? | \|  \| 1 \| Yes \| \| --- \| --- \| --- \| \|  \| 2 \| No \| |
| \|  \|  \|  \|  \| aa8 (required) \| \| --- \| --- \| --- \| --- \| --- \| | Has this healthcare facility been assigned to ART home delivery? | \|  \| 1 \| Yes \| \| --- \| --- \| --- \| \|  \| 2 \| No \| |
| \|  \|  \|  \|  \| aa9 (required) \| \| --- \| --- \| --- \| --- \| --- \| | This participant is eligible to receive ART at home. Please explain to the participant the ART home delivery program.  Does this patient want to receive ART at home? | \|  \| 1 \| Yes \| \| --- \| --- \| --- \| \|  \| 2 \| No \| |
| \|  \|  \|  \|  \| aa10 (required) \| \| --- \| --- \| --- \| --- \| --- \| | Why does the participant NOT want to receive ART at home?  Please type the answer given by the respondent VERBATIM into the provided space. |  |
| \|  \|  \|  \|  \| aa11 (required) \| \| --- \| --- \| --- \| --- \| --- \| | Please give the patient an appointment to be seen by you and the nurse in SIX MONTHS’ time. For patients not receiving home-delivery of ARVs, make sure the patient understands that he/she will need to continue attending the healthcare facility as per their normal schedule.  Please enter the date of the appointment here: |  |
| \|  \|  \|  \| reachable_group > over18_group > hiv_positive > labs_group \| \| --- \| --- \| --- \| --- \| | | |
| \|  \|  \|  \|  \| started_ARVs \| \| --- \| --- \| --- \| --- \| --- \| | Has this patient been started on ARVs at this healthcare facility? | \|  \| 1 \| Yes \| \| --- \| --- \| --- \| \|  \| 2 \| No \| |
| \|  \|  \|  \| date_started_ARVs (required) \| \| --- \| --- \| --- \| --- \| | Please enter the date on which the patient was started on ARVs. |  |
| \|  \|  \|  \| need_VL_CD4 \| \| --- \| --- \| --- \| --- \| | Please take a blood sample and send it for a viral load and a CD4-count measurement. |  |
| \|  \|  \|  \| need_CD4 \| \| --- \| --- \| --- \| --- \| | Please take a blood sample and send it for a CD4-count measurement. |  |
| \|  \|  \|  \| reachable_group > over18_group > hiv_positive > confirm_group \| \| --- \| --- \| --- \| --- \| | | |
| \|  \|  \|  \|  \| confirm_note \| \| --- \| --- \| --- \| --- \| --- \| | Before finalizing this survey on the next page, PLEASE CONFIRM: |  |
| \|  \|  \|  \|  \| eligibility_done (required) \| \| --- \| --- \| --- \| --- \| --- \| | Did you fill out the patient eligibility questions? | \|  \| 1 \| Yes \| \| --- \| --- \| --- \| |
