# Supplementary material for: The impact of community health worker-led home delivery of antiretroviral therapy on virological suppression: a non-inferiority cluster-randomized health systems trial in Dar es Salaam, Tanzania
Source: BMC Health Serv Res. 2017 Feb 22;17:160. doi: 10.1186/s12913-017-2032-7 (PMC5322683; doi:10.1186/s12913-017-2032-7)
Supplement: Additional file 2: — Patient Exit Questionnaire. (DOCX 325 kb) [file 12913_2017_2032_MOESM2_ESM.docx]

#### Patient Exit Questionnaire

| Field | Question | Answer |
| --- | --- | --- |
| intronote | Welcome to the Patient Exit Questionnaire. Please swipe forward to continue. |  |
| 0.1: INTERVIEWER INFORMATION | | |
| \|  \| interviewer_id (required) \| \| --- \| --- \| | Select interviewer name: | \|  \| 1 \| Grace Joseph Matemu \| \| --- \| --- \| --- \| \|  \| 2 \| Geofrey Isdory \| \|  \| 3 \| Glory William \| \|  \| 4 \| Tunkine Sanga \| \|  \| 5 \| Willy Ulkaye \| \|  \| 6 \| Lilian Lwanda \| \|  \| 7 \| Sakina Hamisi \| \|  \| 8 \| Happiness Madadi \| \|  \| 9 \| Mgalama Jaqueline \| \|  \| 10 \| Jeila Maulid \| \|  \| 11 \| Aidath Murusuri \| \|  \| 12 \| Judith Mmari \| \|  \| 13 \| Joan Reno Mori \| \|  \| 14 \| Paul Msale \| \|  \| 15 \| Charles Kafula \| \|  \| 16 \| Josephine Uiso \| \|  \| 17 \| Irene Machume \| \|  \| 18 \| Flora Musa \| \|  \| 97 \| OTHER \| |
| \|  \| 0.1: INTERVIEWER INFORMATION > other_interviewer  Group relevant when: ${interviewer_id} =97 \| \| --- \| --- \| | | |
| \|  \|  \| other_interviewer_note \| \| --- \| --- \| --- \| | You selected 'OTHER' interviewer. Please enter your first and last name. |  |
| \|  \|  \| other_interviewer_first (required) \| \| --- \| --- \| --- \| | First name of interviewer: |  |
| \|  \|  \| other_interviewer_last (required) \| \| --- \| --- \| --- \| | Last name of interviewer: |  |
| \|  \| facility (required) \| \| --- \| --- \| | Select facility: | \|  \| 1 \| MAJI MATITU \| \| --- \| --- \| --- \| \|  \| 2 \| KINGUGI \| \|  \| 3 \| KIMBIJI DISP \| \|  \| 4 \| KISARAWE II \| \|  \| 5 \| KIGAMBONI HOSP \| \|  \| 6 \| MJI MWEMA DISP \| \|  \| 7 \| T/RELI DISP \| \|  \| 8 \| TEMEKE HOSP \| \|  \| 9 \| BUZA DISP \| \|  \| 10 \| ARAFA UGWENO \| \|  \| 11 \| KIBADA DISP \| \|  \| 12 \| SANDALI DESP \| \|  \| 13 \| MBAGALA R/TATU \| \|  \| 14 \| KICHEMCHEM DISP \| \|  \| 15 \| KEKO DISP \| \|  \| 16 \| MAKANGARAWE \| \|  \| 17 \| TOA NGOMA \| \|  \| 18 \| MBAGALA ROUND TABLE \| \|  \| 19 \| Magomeni Health Center \| \|  \| 20 \| Kimara Dispensary \| \|  \| 21 \| Bunju Dispensary \| \|  \| 22 \| Kawe Dispensary \| \|  \| 23 \| Kijitonyama Dispensary \| \|  \| 24 \| Kinondoni Hospital - Other Hospital \| \|  \| 25 \| Makuburi Dispensary \| \|  \| 27 \| Ununio Dispensary \| \|  \| 28 \| Tandale Dispensary \| \|  \| 29 \| Mburahati Dispensary \| \|  \| 30 \| Mwenge Dispensary \| \|  \| 31 \| Mbezi Dispensary \| \|  \| 32 \| Hananasif Dispensary \| \|  \| 33 \| Kigogo Dispensary \| \|  \| 34 \| Mabibo Dispensary \| \|  \| 35 \| Goba Dispensary \| |
| \|  \| ctc2 (required) \| \| --- \| --- \| | What is the CTC2 number of the respondent?  Enter 14 digits.  Response constrained to: string-length(.)<15 and string-length(.)>13 |  |
| \|  \| interview_date (required) \| \| --- \| --- \| | Confirm today's date: |  |
| rsp_id (required) | Counting all of the people you have interviewed today, what number survey is this? |  |
| introduction | Hello, my name is [NAME] and I am working with MDH Tanzania. The reason I am here is because we are conducting health research in Temeke district. |  |
| literate | Can the participant read and write? | \|  \| 1 \| Yes \| \| --- \| --- \| --- \| \|  \| 2 \| No \| |
| \|  \| consented_group > group_munici \| \| --- \| --- \| | | |
| \|  \|  \| note_munici \| \| --- \| --- \| --- \| | In which municipality do you currently live? |  |
| \|  \|  \| municipality_residence (required) \| \| --- \| --- \| --- \| | Select municipality: | \|  \| 1 \| Kinondoni \| \| --- \| --- \| --- \| \|  \| 2 \| Ilala \| \|  \| 3 \| Temeke \| \|  \| 4 \| outside of Dar es Salaam \| |
| \|  \| consented_group > group_ward  Group relevant when: selected( ${municipality_residence} , 1) or selected( ${municipality_residence} , 3) \| \| --- \| --- \| | | |
| \|  \|  \| note_ward \| \| --- \| --- \| --- \| | In which ward do you currently live? |  |
| \|  \|  \| ward_residence (required) \| \| --- \| --- \| --- \| | Select ward: | \|  \| 1 \| Charambe \| \| --- \| --- \| --- \| \|  \| 2 \| Kimbiji \| \|  \| 3 \| Kisarawe II \| \|  \| 4 \| Kigamboni \| \|  \| 5 \| Mjimwema \| \|  \| 6 \| Azimio \| \|  \| 7 \| Mibulani \| \|  \| 8 \| Buza \| \|  \| 9 \| Tandika \| \|  \| 10 \| Kibada \| \|  \| 11 \| Sandali \| \|  \| 12 \| Mbagala Kuu \| \|  \| 13 \| Keko \| \|  \| 14 \| Makangarawe \| \|  \| 15 \| Toangoma \| \|  \| 16 \| Mbagala \| \|  \| 97 \| OTHER Temeke \| \|  \| 17 \| Bunju \| \|  \| 18 \| Goba \| \|  \| 19 \| Hananasifu \| \|  \| 20 \| Kawe \| \|  \| 21 \| Kibamba \| \|  \| 22 \| Kigogo \| \|  \| 23 \| Kijitonyama \| \|  \| 24 \| Kimara \| \|  \| 25 \| Kinondoni \| \|  \| 26 \| Kunduchi \| \|  \| 27 \| Kwembe \| \|  \| 28 \| Mabibo \| \|  \| 29 \| Mabwe Pande \| \|  \| 30 \| Magomeni \| \|  \| 31 \| Makongo \| \|  \| 32 \| Makuburi \| \|  \| 33 \| Makumbusho \| \|  \| 34 \| Makurumla \| \|  \| 35 \| Manzese \| \|  \| 36 \| Mbezi \| \|  \| 37 \| Mbezi Juu \| \|  \| 38 \| Mburahati \| \|  \| 39 \| Mikocheni \| \|  \| 40 \| Msasani \| \|  \| 41 \| Msigani \| \|  \| 42 \| Mwananyamala \| \|  \| 43 \| Mzimuni \| \|  \| 44 \| Ndugumbi \| \|  \| 45 \| Saranga \| \|  \| 46 \| Sinza \| \|  \| 47 \| Tandale \| \|  \| 48 \| Ubungo \| \|  \| 49 \| Wazo \| \|  \| 98 \| OTHER Kinondoni \| |
| \|  \|  \| other_ward \| \| --- \| --- \| --- \| | Please specify if "OTHER" ward:  Question relevant when: selected( ${ward_residence} , 97) or selected( ${ward_residence} , 98) |  |
| \|  \| consented_group > group_mtaa  Group relevant when: ${ward_residence} <17 \| \| --- \| --- \| | | |
| \|  \|  \| note_mtaa \| \| --- \| --- \| --- \| | In which mtaa do you currently live? |  |
| \|  \|  \| mtaa_residence (required) \| \| --- \| --- \| --- \| | Select mtaa: | \|  \| 1 \| Kimbangulile \| \| --- \| --- \| --- \| \|  \| 2 \| Kurasini Mjimpya \| \|  \| 3 \| Nzasa \| \|  \| 4 \| Rangi Tatu \| \|  \| 5 \| Nzasa 'B' \| \|  \| 6 \| Mianzini \| \|  \| 7 \| Majimatitu \| \|  \| 8 \| Mchikichini \| \|  \| 9 \| Majimatitu B' \| \|  \| 10 \| Majimatitu \| \|  \| 971 \| OTHER IN CHARAMBE \| \|  \| 12 \| Mikenge \| \|  \| 13 \| Kizito \| \|  \| 14 \| Ngobanya \| \|  \| 15 \| Kwa chale \| \|  \| 972 \| OTHER IN KIMBIJI \| \|  \| 16 \| Vumilia Ukooni \| \|  \| 17 \| Mwasonga \| \|  \| 973 \| OTHER IN KISARAWE II \| \|  \| 18 \| Kigamboni \| \|  \| 19 \| Ferry \| \|  \| 20 \| Tuamoyo \| \|  \| 974 \| OTHER IN KIGAMBONI \| \|  \| 21 \| Mjimwema \| \|  \| 22 \| Maweni \| \|  \| 23 \| Ungindoni \| \|  \| 24 \| Kibugumo \| \|  \| 975 \| OTHER IN MJIMWEMA \| \|  \| 25 \| Kichangani \| \|  \| 26 \| Tambukareli \| \|  \| 27 \| Mjimpya \| \|  \| 28 \| Mbuyuni \| \|  \| 29 \| Azimio \| \|  \| 30 \| Azimio Kurasini \| \|  \| 31 \| Mtongani \| \|  \| 976 \| OTHER IN AZIMIO \| \|  \| 32 \| Keko Juu \| \|  \| 33 \| Wailes \| \|  \| 34 \| Mibulani \| \|  \| 977 \| OTHER IN MIBULANI \| \|  \| 35 \| Buza \| \|  \| 36 \| Machine ya Maji \| \|  \| 37 \| Mjimpya \| \|  \| 978 \| OTHER IN BUZA \| \|  \| 38 \| Tamla \| \|  \| 39 \| Kilimahewa \| \|  \| 40 \| Mabatini \| \|  \| 979 \| OTHER IN TANDIKA \| \|  \| 41 \| Kifurukwe \| \|  \| 42 \| Uvumba \| \|  \| 43 \| Nyakwale \| \|  \| 44 \| Kiziza \| \|  \| 45 \| Kichangani \| \|  \| 46 \| Sokoni \| \|  \| 9710 \| OTHER IN KIBADA \| \|  \| 47 \| Mkwinda \| \|  \| 48 \| Mnofu \| \|  \| 49 \| Mamboleo \| \|  \| 50 \| Tindwa \| \|  \| 51 \| Mpogo \| \|  \| 52 \| Vetenary \| \|  \| 53 \| Mwembeladu \| \|  \| 54 \| Kimbunga \| \|  \| 55 \| Usalama \| \|  \| 9711 \| OTHER IN SANDALI \| \|  \| 56 \| Mbagala Kuu \| \|  \| 57 \| Mbagala Kuu Kaskazini \| \|  \| 58 \| Mbagala Kuu Mashariki \| \|  \| 59 \| Jeshi La Wokovu \| \|  \| 60 \| Kichemichemi \| \|  \| 61 \| Kizuiani \| \|  \| 62 \| Makuka \| \|  \| 9712 \| OTHER IN MBAGALA KUU \| \|  \| 63 \| Magurumbasi A \| \|  \| 64 \| Keko Mwanga B' \| \|  \| 65 \| Magurumbasi B \| \|  \| 9713 \| OTHER IN KEKO \| \|  \| 66 \| Yombo dovya \| \|  \| 89 \| Makangarawe \| \|  \| 67 \| Makangarawe \| \|  \| 9714 \| OTHER IN MAKANGARAWE \| \|  \| 68 \| Kongowe \| \|  \| 69 \| Masaki \| \|  \| 70 \| Mponde \| \|  \| 71 \| Goroka \| \|  \| 72 \| Toangoma \| \|  \| 73 \| Mikwambe \| \|  \| 74 \| Masuliza \| \|  \| 75 \| Ponde \| \|  \| 76 \| Mzinga \| \|  \| 9715 \| OTHER IN TOANGOMA \| \|  \| 77 \| Kizinga \| \|  \| 78 \| Bughdadi \| \|  \| 79 \| Mangaya \| \|  \| 80 \| Moringe \| \|  \| 81 \| Mbagala \| \|  \| 9716 \| OTHER IN MBAGALA \| |
| \|  \|  \| other_mtaa \| \| --- \| --- \| --- \| | Please specify if 'OTHER' mtaa:  Question relevant when: ${mtaa_residence} >970 |  |
| \|  \| a1 (required) \| \| --- \| --- \| | WHAT IS THE SEX OF THE RESPONDENT?  Only ask if not obvious. | \|  \| 1 \| Female \| \| --- \| --- \| --- \| \|  \| 2 \| Male \| |
| \|  \| a2 (required) \| \| --- \| --- \| | How old are you?  Please enter the age in years. If respondent does not know, but confirms that he/she is over 18, enter 1111. |  |
| below18 | Thank you for your time. Have a nice day.  You cannot continue because the participant is under 18. Save and exit this form.  Question relevant when: ${a2} <18 |  |
| over18_group  Group relevant when: ${a2} >17 and ${consent_yn} =1 | | |
| \|  \| a3 (required) \| \| --- \| --- \| | Are you married? | \|  \| 1 \| Yes (incl. civil and custom marriage) \| \| --- \| --- \| --- \| \|  \| 2 \| No, but I live with my partner \| \|  \| 3 \| No, and I live alone or with other family members \| \|  \| 4 \| Divorced/separated from husband/wife \| \|  \| 5 \| Widowed \| \|  \| 99 \| REFUSED \| \|  \| 6 \| Other (specify below) \| |
| \|  \| a3_other (required) \| \| --- \| --- \| | Please specify 'Other':  Question relevant when: ${a3} =6 |  |
| \|  \| a4 (required) \| \| --- \| --- \| | What type of clinic visit did you attend today? | \|  \| 1 \| HIV testing and counseling \| \| --- \| --- \| --- \| \|  \| 2 \| For Post-Exposure Prophylaxis (PEP) \| \|  \| 3 \| Pre-ART enrolment \| \|  \| 4 \| Pre-ART client for follow up visit \| \|  \| 5 \| ART initiation \| \|  \| 6 \| Follow up for existing ART client with ART pick up \| \|  \| 7 \| Follow up for existing ART client without ART pickup \| \|  \| 8 \| PMTCT with ART pick-up \| \|  \| 9 \| PMTCT without ART pick-up \| \|  \| 97 \| Other (specify on next page) \| \|  \| 99 \| REFUSED \| |
| \|  \| a4_other (required) \| \| --- \| --- \| | Please specify 'Other' reason for attending clinic today:  Question relevant when: ${a4} =97 |  |
| \|  \| a4_hivrelate (required) \| \| --- \| --- \| | DID RESPONDENT COME TO CLINIC TODAY FOR HIV-RELATED SERVICES?  Question relevant when: ${a4} =97 | \|  \| 1 \| Yes \| \| --- \| --- \| --- \| \|  \| 2 \| No \| |
| \|  \| a4_hivno \| \| --- \| --- \| | Thank you for your time. Have a nice day.  You cannot continue because the participant did not come for HIV-related services. Please save and exit this questionnaire.  Question relevant when: ${a4_hivrelate} =2 |  |
| \|  \| over18_group > hivyes  Group relevant when: ${a4} <97 or ${a4_hivrelate} =1 \| \| --- \| --- \| | | |
| \|  \|  \| over18_group > hivyes > a5-13_group \| \| --- \| --- \| --- \| | | |
| \|  \|  \|  \| a5_pre (required) \| \| --- \| --- \| --- \| --- \| | IS THE RESPONDENT HIV-POSITIVE? | \|  \| 1 \| Yes \| \| --- \| --- \| --- \| \|  \| 2 \| No \| |
| \|  \|  \|  \| not_hiv_positive \| \| --- \| --- \| --- \| --- \| | Thank you for your time. Have a nice day.  You cannot continue because the participant is not HIV-positive. Swipe forward to save and exit this form.  Question relevant when: ${a5_pre} =2 |  |
| \|  \|  \|  \| over18_group > hivyes > a5-13_group > hiv_positive  Group relevant when: ${a5_pre} =1 \| \| --- \| --- \| --- \| --- \| | | |
| \|  \|  \|  \|  \| a5 (required) \| \| --- \| --- \| --- \| --- \| --- \| | When did you find out that you are HIV-positive?  If respondent DK, enter February 1980. If respondent RF, enter March 1980.  Response constrained to: . <= today() |  |
| \|  \|  \|  \|  \| a6 (required) \| \| --- \| --- \| --- \| --- \| --- \| | When did you first attend an ART clinic?  Ensure that the patient understands that coming to the ART clinics can be for both pre-ART or ART services If respondent only ever came for PMTCT, enter January 1980. If respondent DK, enter February 1980. If respondent RF, enter March 1980.  Response constrained to: . <= today() |  |
| \|  \|  \|  \|  \| a7 (required) \| \| --- \| --- \| --- \| --- \| --- \| | Are you currently receiving antiretroviral treatment (ART)? | \|  \| 1 \| Yes \| \| --- \| --- \| --- \| \|  \| 2 \| No \| \|  \| 99 \| REFUSED \| |
| \|  \|  \|  \|  \| over18_group > hivyes > a5-13_group > hiv_positive > yes_ART  Group relevant when: ${a7} =1 \| \| --- \| --- \| --- \| --- \| --- \| | | |
| \|  \|  \|  \|  \|  \| over18_group > hivyes > a5-13_group > hiv_positive > yes_ART > a8_group \| \| --- \| --- \| --- \| --- \| --- \| --- \| | | |
| \|  \|  \|  \|  \|  \|  \| a8 \| \| --- \| --- \| --- \| --- \| --- \| --- \| --- \| | For how many months or years have you been attending this clinic to get your ART? |  |
| \|  \|  \|  \|  \|  \|  \| a8_months \| \| --- \| --- \| --- \| --- \| --- \| --- \| --- \| | Months:  Response constrained to: .>-1 |  |
| \|  \|  \|  \|  \|  \|  \| a8_years (required) \| \| --- \| --- \| --- \| --- \| --- \| --- \| --- \| | Years:  Question relevant when: ${a8_months} = null  Response constrained to: if(string-length(.) >= 1, string-length( ${a8_months} ) < 1, string-length( ${a8_months} ) >=1) and .>-1 |  |
| \|  \|  \|  \|  \|  \| over18_group > hivyes > a5-13_group > hiv_positive > yes_ART > less_than_year_ART  Group relevant when: ${a8_months} <13 or ${a8_years} <2 \| \| --- \| --- \| --- \| --- \| --- \| --- \| | | |
| \|  \|  \|  \|  \|  \|  \| a9 (required) \| \| --- \| --- \| --- \| --- \| --- \| --- \| --- \| | Which clinic were you attending for your ART before? | \|  \| 1 \| MAJI MATITU \| \| --- \| --- \| --- \| \|  \| 2 \| KINGUGI \| \|  \| 3 \| KIMBIJI DISP \| \|  \| 4 \| KISARAWE II \| \|  \| 5 \| KIGAMBONI HOSP \| \|  \| 6 \| MJI MWEMA DISP \| \|  \| 7 \| T/RELI DISP \| \|  \| 8 \| TEMEKE HOSP \| \|  \| 9 \| BUZA DISP \| \|  \| 10 \| ARAFA UGWENO \| \|  \| 11 \| KIBADA DISP \| \|  \| 12 \| SANDALI DESP \| \|  \| 13 \| MBAGALA R/TATU \| \|  \| 14 \| KICHEMCHEM DISP \| \|  \| 15 \| KEKO DISP \| \|  \| 16 \| MAKANGARAWE \| \|  \| 17 \| TOA NGOMA \| \|  \| 18 \| MBAGALA ROUND TABLE \| \|  \| 96 \| None \| \|  \| 97 \| Other \| \|  \| 98 \| Don't know \| \|  \| 99 \| Refused \| |
| \|  \|  \|  \|  \|  \|  \| a9_other (required) \| \| --- \| --- \| --- \| --- \| --- \| --- \| --- \| | Please specify 'Other' clinic you attended for ART before.  Question relevant when: ${a9} =97 |  |
| \|  \|  \|  \|  \|  \|  \| over18_group > hivyes > a5-13_group > hiv_positive > yes_ART > less_than_year_ART > a10_group  Group relevant when: ${a9} <96 or ${a9} =97 \| \| --- \| --- \| --- \| --- \| --- \| --- \| --- \| | | |
| \|  \|  \|  \|  \|  \|  \|  \| a10 \| \| --- \| --- \| --- \| --- \| --- \| --- \| --- \| --- \| | For how many months or years had you been attending that clinic to get your ART? |  |
| \|  \|  \|  \|  \|  \|  \|  \| a10_months \| \| --- \| --- \| --- \| --- \| --- \| --- \| --- \| --- \| | Months:  Response constrained to: .>-1 |  |
| \|  \|  \|  \|  \|  \|  \|  \| a10_years (required) \| \| --- \| --- \| --- \| --- \| --- \| --- \| --- \| --- \| | Years:  Question relevant when: ${a10_months} = null  Response constrained to: if(string-length(.) >= 1, string-length( ${a10_months} ) < 1, string-length( ${a10_months} ) >=1) and .>-1 |  |
| \|  \|  \|  \|  \|  \| a11 (required) \| \| --- \| --- \| --- \| --- \| --- \| --- \| | When did you first begin receiving ART?  If respondent DK, enter February 1980. If respondent RF, enter March 1980.  Question relevant when: ${a7} =1  Response constrained to: . <= today() |  |
| \|  \|  \|  \|  \| over18_group > hivyes > a5-13_group > hiv_positive > no_response_a11  Group relevant when: string-length( ${a11} )<1 \| \| --- \| --- \| --- \| --- \| --- \| | | |
| \|  \|  \|  \|  \|  \| a12 (required) \| \| --- \| --- \| --- \| --- \| --- \| --- \| | Have you been on ART at any time in the past? | \|  \| 1 \| Yes \| \| --- \| --- \| --- \| \|  \| 2 \| No \| \|  \| 99 \| REFUSED \| |
| \|  \|  \|  \|  \|  \| over18_group > hivyes > a5-13_group > hiv_positive > no_response_a11 > a12_yes  Group relevant when: ${a12} =1 \| \| --- \| --- \| --- \| --- \| --- \| --- \| | | |
| \|  \|  \|  \|  \|  \|  \| a13 (required) \| \| --- \| --- \| --- \| --- \| --- \| --- \| --- \| | Why did you receive ART?  If patient answers for treatment of HIV, probe to ensure that this was not for preventing infection of a baby (i.e. PMTCT).  Question relevant when: ${a12} =1 | \|  \| 1 \| For treatment of HIV \| \| --- \| --- \| --- \| \|  \| 2 \| For PMTCT \| \|  \| 3 \| As Post-Exposure Prophylaxis (PEP) \| \|  \| 97 \| Other (specify on next page) \| \|  \| 99 \| REFUSED \| |
| \|  \|  \|  \|  \|  \|  \| a13_other (required) \| \| --- \| --- \| --- \| --- \| --- \| --- \| --- \| | Please specify 'Other' reason for receiving ART.  Question relevant when: ${a13} =97 |  |
| \|  \|  \|  \|  \|  \|  \| a14 (required) \| \| --- \| --- \| --- \| --- \| --- \| --- \| --- \| | Why are you not on ART currently?  Question relevant when: ${a7} =2 and ${a13} !=3 | \|  \| 1 \| Patient choice \| \| --- \| --- \| --- \| \|  \| 2 \| Stopped by doctor \| \|  \| 97 \| Other (specify on next page) \| \|  \| 99 \| REFUSED \| |
| \|  \|  \|  \|  \| a15 (required) \| \| --- \| --- \| --- \| --- \| --- \| | Are you currently in full-time or part-time education? | \|  \| 1 \| Yes, full-time \| \| --- \| --- \| --- \| \|  \| 2 \| Yes, part-time \| \|  \| 3 \| Not currently in education \| \|  \| 99 \| REFUSED \| |
| \|  \|  \|  \|  \| over18_group > hivyes > a5-13_group > hiv_positive > education_current \| \| --- \| --- \| --- \| --- \| --- \| | | |
| \|  \|  \|  \|  \|  \| a16 (required) \| \| --- \| --- \| --- \| --- \| --- \| --- \| | What type of education facility are you attending?  Question relevant when: ${a15} =1 or ${a15} =2 | \|  \| 1 \| Lower primary school (Grades 1-2) \| \| --- \| --- \| --- \| \|  \| 2 \| Higher primary school (Standard 1-5) \| \|  \| 3 \| Junior secondary school (Form 1-3) \| \|  \| 4 \| Senior secondary school (Form 4-5) \| \|  \| 5 \| Teacher-training College \| \|  \| 6 \| Nursing Assistant College \| \|  \| 7 \| Nursing College \| \|  \| 8 \| Other technical or vocational college \| \|  \| 9 \| University (studying towards Bachelor) \| \|  \| 10 \| University (studying towards Master) \| \|  \| 11 \| University (studying medicine or law) \| \|  \| 12 \| University (studying towards PhD) \| \|  \| 99 \| REFUSED \| \|  \| 13 \| Other (specify) \| |
| \|  \|  \|  \|  \| a16_other (required) \| \| --- \| --- \| --- \| --- \| --- \| | Please specify 'Other' education facility type:  Question relevant when: ${a16} =13 |  |
| \|  \|  \|  \|  \| over18_group > hivyes > a5-13_group > hiv_positive > education_highest \| \| --- \| --- \| --- \| --- \| --- \| | | |
| \|  \|  \|  \|  \|  \| a17 (required) \| \| --- \| --- \| --- \| --- \| --- \| --- \| | What is the highest level of school you attended: preschool, primary school, secondary school, or high school? | \|  \| 1 \| None \| \| --- \| --- \| --- \| \|  \| 2 \| Preschool \| \|  \| 3 \| Primary school \| \|  \| 4 \| Secondary school \| \|  \| 5 \| High school \| \|  \| 99 \| REFUSED \| \|  \| 97 \| Other (specify) \| |
| \|  \|  \|  \|  \| a17_other (required) \| \| --- \| --- \| --- \| --- \| --- \| | Please specify 'Other' education level:  Question relevant when: ${a17} =97 |  |
| \|  \|  \|  \|  \| over18_group > hivyes > a5-13_group > hiv_positive > education_level \| \| --- \| --- \| --- \| --- \| --- \| | | |
| \|  \|  \|  \|  \|  \| a18_a (required) \| \| --- \| --- \| --- \| --- \| --- \| --- \| | What is the highest grade/form/standard you completed at that level?  If completed less than one year at that level, record ‘100’. |  |
| \|  \|  \|  \|  \|  \| a18_b (required) \| \| --- \| --- \| --- \| --- \| --- \| --- \| | Select grade, form, or standard: | \|  \| Grade \| Grade \| \| --- \| --- \| --- \| \|  \| Form \| Form \| \|  \| Standard \| Standard \| |
| \|  \|  \|  \|  \| a19 (required) \| \| --- \| --- \| --- \| --- \| --- \| | Have you completed any education after school? | \|  \| 1 \| Yes \| \| --- \| --- \| --- \| \|  \| 2 \| No \| \|  \| 99 \| REFUSED \| |
| \|  \|  \|  \|  \| over18_group > hivyes > a5-13_group > hiv_positive > highest_after \| \| --- \| --- \| --- \| --- \| --- \| | | |
| \|  \|  \|  \|  \|  \| a20 (required) \| \| --- \| --- \| --- \| --- \| --- \| --- \| | What is the highest level of education you completed after school?  Question relevant when: ${a19} =1 | \|  \| 1 \| Teacher-training College \| \| --- \| --- \| --- \| \|  \| 2 \| Nursing Assistant College \| \|  \| 3 \| Nursing College \| \|  \| 4 \| Other technical or vocational college \| \|  \| 5 \| University (studying towards Bachelor) \| \|  \| 6 \| University (studying towards Master) \| \|  \| 7 \| University (studying medicine or law) \| \|  \| 8 \| University (studying towards PhD) \| \|  \| 99 \| REFUSED \| \|  \| 97 \| Other (specify) \| |
| \|  \|  \|  \|  \| a20_other (required) \| \| --- \| --- \| --- \| --- \| --- \| | Please specify 'Other' education level:  Question relevant when: ${a20} =97 |  |
| \|  \|  \|  \|  \| a21 (required) \| \| --- \| --- \| --- \| --- \| --- \| | Have you done any work in the last six months? With work, we mean any activity to earn money or obtain food. | \|  \| 1 \| Yes \| \| --- \| --- \| --- \| \|  \| 2 \| No \| \|  \| 99 \| REFUSED \| |
| \|  \|  \|  \|  \| a22 (required) \| \| --- \| --- \| --- \| --- \| --- \| | What is your occupation? That is, what kind of work do you mainly do?  Question relevant when: ${a21} =1 | \|  \| 1 \| Farm work \| \| --- \| --- \| --- \| \|  \| 2 \| Domestic work \| \|  \| 3 \| Construction work \| \|  \| 4 \| Security work \| \|  \| 5 \| Cleaning work \| \|  \| 6 \| Small business owner \| \|  \| 7 \| Mine work \| \|  \| 8 \| Teacher \| \|  \| 9 \| Traditional healer \| \|  \| 10 \| Nurse or Nurse assistant \| \|  \| 11 \| Physician or Surgeon \| \|  \| 12 \| Other healthcare worker \| \|  \| 13 \| Game farm / game reserve (eg ranger) \| \|  \| 14 \| Driver \| \|  \| 15 \| Skiller worker (eg plumber, mechanic, electrician) \| \|  \| 16 \| Cook / chef / catering \| \|  \| 17 \| Unskilled worker (eg general labourer) \| \|  \| 18 \| Artisan (eg carpenter, woodcarver, weaver) \| \|  \| 19 \| Waiter / barman \| \|  \| 20 \| Informal selling \| \|  \| 21 \| Small business assistant \| \|  \| 22 \| Clerical and office work \| \|  \| 23 \| Cattle herder \| \|  \| 24 \| Sewing, hairdressing, baking, brewing \| \|  \| 25 \| Police, soldier, fireman \| \|  \| 26 \| Petrol attendant \| \|  \| 27 \| Timber, sawmill, poles \| \|  \| 28 \| Gardening services \| \|  \| 29 \| Fieldworker - NGO or university \| \|  \| 30 \| Art, craft, photography, fashion design \| \|  \| 31 \| Senior administrator, manager, professional \| \|  \| 32 \| Priest / pastor \| \|  \| 33 \| Other \| \|  \| 98 \| Don't know \| \|  \| RF \| Refused \| |
| \|  \|  \|  \|  \| a22_other (required) \| \| --- \| --- \| --- \| --- \| --- \| | YOU SELECTED 'Other healthcare worker' OR 'Other'. PLEASE SPECIFY:  Question relevant when: ${a22} =12 or ${a22} =33 |  |
| \|  \|  \|  \|  \| over18_group > hivyes > a5-13_group > hiv_positive > not_working \| \| --- \| --- \| --- \| --- \| --- \| | | |
| \|  \|  \|  \|  \|  \| a23 (required) \| \| --- \| --- \| --- \| --- \| --- \| --- \| | Why have you not worked in the last 12 months?  Question relevant when: ${a21} =2 | \|  \| 1 \| Was in full-time education \| \| --- \| --- \| --- \| \|  \| 2 \| Unable to work (disabled) \| \|  \| 3 \| Unemployed \| \|  \| 4 \| Homemaker \| \|  \| 5 \| Looked after my (grand) children \| \|  \| 6 \| Could not work because of pregnancy \| \|  \| 7 \| Retired \| \|  \| 8 \| Sick leave \| \|  \| 9 \| Other leave \| \|  \| 97 \| Other (specify) \| \|  \| 99 \| REFUSED \| |
| \|  \|  \|  \|  \| a23_other (required) \| \| --- \| --- \| --- \| --- \| --- \| | Please specify 'Other' reason for not working in the last 12 months:  Question relevant when: ${a23} =97 |  |
| \|  \|  \|  \|  \| a24 (required) \| \| --- \| --- \| --- \| --- \| --- \| | Overall, how satisfied or dissatisfied are you with the healthcare services in your area?  Ask the respondent to refer to scale 1.  0=Very dissatisfied  10=Very satisfied | \|  \| 0 \| 0 (Very dissatisfied) \| \| --- \| --- \| --- \| \|  \| 1 \| 1 \| \|  \| 2 \| 2 \| \|  \| 3 \| 3 \| \|  \| 4 \| 4 \| \|  \| 5 \| 5 \| \|  \| 6 \| 6 \| \|  \| 7 \| 7 \| \|  \| 8 \| 8 \| \|  \| 9 \| 9 \| \|  \| 10 \| 10 (Very satisfied) \| \|  \| 99 \| REFUSED \| |
| \|  \|  \|  \|  \| a25 (required) \| \| --- \| --- \| --- \| --- \| --- \| | How satisfied or dissatisfied are you with the healthcare services for HIV in your area?  Ask the respondent to refer to scale 1.  0=Very dissatisfied  10=Very satisfied | \|  \| 0 \| 0 (Very dissatisfied) \| \| --- \| --- \| --- \| \|  \| 1 \| 1 \| \|  \| 2 \| 2 \| \|  \| 3 \| 3 \| \|  \| 4 \| 4 \| \|  \| 5 \| 5 \| \|  \| 6 \| 6 \| \|  \| 7 \| 7 \| \|  \| 8 \| 8 \| \|  \| 9 \| 9 \| \|  \| 10 \| 10 (Very satisfied) \| \|  \| 99 \| REFUSED \| |
| \|  \|  \|  \|  \| b_intro \| \| --- \| --- \| --- \| --- \| --- \| | PART 2: PATIENT SATISFACTION  The following section asks you about your experience today at the clinic. For these questions, you are asked to rate your experience with very good, good, moderate, bad or very bad.  Ask the patient to refer to scale 2. |  |
| \|  \|  \|  \|  \| b1 (required) \| \| --- \| --- \| --- \| --- \| --- \| | Please think about your visit to the clinic today beginning from the time you arrived up until now.  How would you rate your experience with the service you received today?  1=Very good  2=Good  3=Moderate  4=Bad  5=Very bad | \|  \| 1 \| 1 \| \| --- \| --- \| --- \| \|  \| 2 \| 2 \| \|  \| 3 \| 3 \| \|  \| 4 \| 4 \| \|  \| 5 \| 5 \| \|  \| 99 \| REFUSED \| |
| \|  \|  \|  \|  \| b2 (required) \| \| --- \| --- \| --- \| --- \| --- \| | Would you recommend your friend or relative to attend this facility for HIV care? | \|  \| 1 \| Yes \| \| --- \| --- \| --- \| \|  \| 2 \| No \| \|  \| 99 \| REFUSED \| |
| \|  \|  \|  \|  \| b3_a (required) \| \| --- \| --- \| --- \| --- \| --- \| | Overall, how would you rate the amount of time you waited before being attended to?  1=Very good  2=Good  3=Moderate  4=Bad  5=Very bad | \|  \| 1 \| 1 \| \| --- \| --- \| --- \| \|  \| 2 \| 2 \| \|  \| 3 \| 3 \| \|  \| 4 \| 4 \| \|  \| 5 \| 5 \| \|  \| 99 \| REFUSED \| |
| \|  \|  \|  \|  \| b3_b (required) \| \| --- \| --- \| --- \| --- \| --- \| | John always attends his visits to the HIV clinic. Each time he visits the clinic he has to wait about 30 minutes before he can see the nurse or doctor.  Overall, how would you rate the amount of time John waited before being attended to?  1=Very good  2=Good  3=Moderate  4=Bad  5=Very bad | \|  \| 1 \| 1 \| \| --- \| --- \| --- \| \|  \| 2 \| 2 \| \|  \| 3 \| 3 \| \|  \| 4 \| 4 \| \|  \| 5 \| 5 \| \|  \| 99 \| REFUSED \| |
| \|  \|  \|  \|  \| b3_c (required) \| \| --- \| --- \| --- \| --- \| --- \| | Neema always attends her visits to the HIV clinic. The clinic is always busy. Each time she visits the clinic she usually has to wait about 1-2 hours before she can see the nurse or doctor. On occasion, she has waited for the whole day and then had to leave without seeing a doctor.  Overall, how would you rate the amount of time Neema waited before being attended to?  1=Very good  2=Good  3=Moderate  4=Bad  5=Very bad | \|  \| 1 \| 1 \| \| --- \| --- \| --- \| \|  \| 2 \| 2 \| \|  \| 3 \| 3 \| \|  \| 4 \| 4 \| \|  \| 5 \| 5 \| \|  \| 99 \| REFUSED \| |
| \|  \|  \|  \|  \| b4_a (required) \| \| --- \| --- \| --- \| --- \| --- \| | Overall, how would you rate your experience of getting involved as much as you wanted to be in making decisions about your care or treatment?  1=Very good  2=Good  3=Moderate  4=Bad  5=Very bad | \|  \| 1 \| 1 \| \| --- \| --- \| --- \| \|  \| 2 \| 2 \| \|  \| 3 \| 3 \| \|  \| 4 \| 4 \| \|  \| 5 \| 5 \| \|  \| 99 \| REFUSED \| |
| \|  \|  \|  \|  \| b4_b (required) \| \| --- \| --- \| --- \| --- \| --- \| | Mary attends the ART clinic regularly. She has discussed her preference to change one of her ARVs. Her doctor adjusted the prescriptions to take account of her preference. She was also pregnant and concerned about infecting her baby with HIV during delivery. The doctor discussed different delivery options with her and helped her prepare a birth plan.  Overall, how would you rate Mary’s experience of getting involved as much as she wanted to be in making decisions about her care or treatment?  1=Very good  2=Good  3=Moderate  4=Bad  5=Very bad | \|  \| 1 \| 1 \| \| --- \| --- \| --- \| \|  \| 2 \| 2 \| \|  \| 3 \| 3 \| \|  \| 4 \| 4 \| \|  \| 5 \| 5 \| \|  \| 99 \| REFUSED \| |
| \|  \|  \|  \|  \| b4_c (required) \| \| --- \| --- \| --- \| --- \| --- \| | Mohamed attends the ART clinic regularly. On this visit, he complains to the doctor that he wasn’t reacting well to his ARVs and would like them changed. The doctor reassures him but still prescribes the same ARVs. He also asks him to perform some tests without explaining why or asking for his permission. He only tells him that the tests are important.  Overall, how would you rate Mohamed’s experience of getting involved as much as he wanted to be in making decisions about his care or treatment?  1=Very good  2=Good  3=Moderate  4=Bad  5=Very bad | \|  \| 1 \| 1 \| \| --- \| --- \| --- \| \|  \| 2 \| 2 \| \|  \| 3 \| 3 \| \|  \| 4 \| 4 \| \|  \| 5 \| 5 \| \|  \| 99 \| REFUSED \| |
| \|  \|  \|  \|  \| b5_a (required) \| \| --- \| --- \| --- \| --- \| --- \| | Overall, how would you rate the experience of how much time you spent seeing the health provider?  1=Very good  2=Good  3=Moderate  4=Bad  5=Very bad | \|  \| 1 \| 1 \| \| --- \| --- \| --- \| \|  \| 2 \| 2 \| \|  \| 3 \| 3 \| \|  \| 4 \| 4 \| \|  \| 5 \| 5 \| \|  \| 99 \| REFUSED \| |
| \|  \|  \|  \|  \| b5_b (required) \| \| --- \| --- \| --- \| --- \| --- \| | Lawrence goes to the clinic for his regular HIV care visit. When it gets to his turn to see the nurse, the nurse spends over 20 minutes talking with him. The nurse asks him some questions, listens to his answers, and also answers all the questions Lawrence asks.  Overall, how would you rate the experience of how much time [name] spent seeing the health provider?  1=Very good  2=Good  3=Moderate  4=Bad  5=Very bad | \|  \| 1 \| 1 \| \| --- \| --- \| --- \| \|  \| 2 \| 2 \| \|  \| 3 \| 3 \| \|  \| 4 \| 4 \| \|  \| 5 \| 5 \| \|  \| 99 \| REFUSED \| |
| \|  \|  \|  \|  \| b5_c (required) \| \| --- \| --- \| --- \| --- \| --- \| | Farida goes to the clinic for her regular HIV care visit. When it gets to her turn, the nurse asks her a few questions without looking up, writes down some notes and dismisses her in less than 10 minutes.  Overall, how would you rate the experience of how much time Farida spent seeing the health provider?  1=Very good  2=Good  3=Moderate  4=Bad  5=Very bad | \|  \| 1 \| 1 \| \| --- \| --- \| --- \| \|  \| 2 \| 2 \| \|  \| 3 \| 3 \| \|  \| 4 \| 4 \| \|  \| 5 \| 5 \| \|  \| 99 \| REFUSED \| |
| \|  \|  \|  \|  \| b6_a (required) \| \| --- \| --- \| --- \| --- \| --- \| | Overall, how would you rate your experience of being greeted and talked to respectfully?  1=Very good  2=Good  3=Moderate  4=Bad  5=Very bad | \|  \| 1 \| 1 \| \| --- \| --- \| --- \| \|  \| 2 \| 2 \| \|  \| 3 \| 3 \| \|  \| 4 \| 4 \| \|  \| 5 \| 5 \| \|  \| 99 \| REFUSED \| |
| \|  \|  \|  \|  \| b6_b (required) \| \| --- \| --- \| --- \| --- \| --- \| | Nuru attends the ART clinic for the first time. She didn't know how the clinic worked so she spoke to the first person she saw in nurse uniform. The person greeted her and escorted her to the room where she could receive HIV care. When she got there, she was greeted by a nurse and asked what she wanted. Throughout the discussion, the nurse gave Nuru her full attention.  Overall, how would you rate Nuru’s experience of being greeted and talked to respectfully?  1=Very good  2=Good  3=Moderate  4=Bad  5=Very bad | \|  \| 1 \| 1 \| \| --- \| --- \| --- \| \|  \| 2 \| 2 \| \|  \| 3 \| 3 \| \|  \| 4 \| 4 \| \|  \| 5 \| 5 \| \|  \| 99 \| REFUSED \| |
| \|  \|  \|  \|  \| b6_c (required) \| \| --- \| --- \| --- \| --- \| --- \| | Joseph attends the ART clinic for the first time. He didn't know how the clinic worked so he spoke to the first person he saw in nurse uniform who told him: "go sit there and wait, we will call you". After a few minutes, the nurse pointed to him and said "come". Without looking at him, the nurse made some notes, and kept on talking to another nurse about something that had happened to a friend of hers.  Overall, how would you rate Joseph’s experience of being greeted and talked to respectfully? Please circle a number.  1=Very good  2=Good  3=Moderate  4=Bad  5=Very bad | \|  \| 1 \| 1 \| \| --- \| --- \| --- \| \|  \| 2 \| 2 \| \|  \| 3 \| 3 \| \|  \| 4 \| 4 \| \|  \| 5 \| 5 \| \|  \| 99 \| REFUSED \| |
| \|  \|  \|  \|  \| note_c \| \| --- \| --- \| --- \| --- \| --- \| | PART 3: HEALTH SERVICE UTILIZATION AND EXPENDITURE  I would now like to ask you a few questions about the costs you experience to access healthcare. This question is only about the cost for YOUR OWN healthcare, NOT for other people in your household. |  |
| \|  \|  \|  \|  \| c1 (required) \| \| --- \| --- \| --- \| --- \| --- \| | Are you covered by a scheme, such as an insurance, that helps you pay for healthcare? | \|  \| 1 \| Yes \| \| --- \| --- \| --- \| \|  \| 2 \| No \| \|  \| 99 \| REFUSED \| |
| \|  \|  \|  \|  \| c2 (required) \| \| --- \| --- \| --- \| --- \| --- \| | For your clinic visit TODAY, what type of expenses did you pay for?  Ensure that the respondent only counts expenses since he/she started travelling to the clinic until the time of the interview.  Response constrained to: if(selected(., '99'), count-selected(.)=1, count-selected(.)>=1) | \|  \| 1 \| Consultation fees \| \| --- \| --- \| --- \| \|  \| 2 \| Medical tests \| \|  \| 3 \| Medicines \| \|  \| 4 \| Transport to get to the clinic (one way) \| \|  \| 5 \| Payment for someone to look after your children while you are gone to the clinic \| \|  \| 6 \| Food since you started travelling to the clinic until the time of the interview \| \|  \| 7 \| Phone calls/SMS since you started travelling to the clinic until the time of the interview \| \|  \| 8 \| Others (specify on next page) \| \|  \| 99 \| REFUSED \| |
| \|  \|  \|  \|  \| c2_other (required) \| \| --- \| --- \| --- \| --- \| --- \| | Please specify 'Other' clinic expenses:  Question relevant when: selected( ${c2} , '8') |  |
| \|  \|  \|  \|  \| c2_a (required) \| \| --- \| --- \| --- \| --- \| --- \| | How much did you pay for: Consultation fee?  Please enter the amount in Tanzanian Shilling. If DK, enter -98. If RF, enter -99.  Question relevant when: selected( ${c2} , '1')  Response constrained to: .>-1 or .=-98 or .=-99 |  |
| \|  \|  \|  \|  \| c2_b (required) \| \| --- \| --- \| --- \| --- \| --- \| | How much did you pay for: Medical tests?  Please enter the amount in Tanzanian Shilling. If DK, enter -98. If RF, enter -99.  Question relevant when: selected( ${c2} , '2')  Response constrained to: .>-1 or .=-98 or .=-99 |  |
| \|  \|  \|  \|  \| c2_c (required) \| \| --- \| --- \| --- \| --- \| --- \| | How much did you pay for: Medicines?  Please enter the amount in Tanzanian Shilling. If DK, enter -98. If RF, enter -99.  Question relevant when: selected( ${c2} , '3')  Response constrained to: .>-1 or .=-98 or .=-99 |  |
| \|  \|  \|  \|  \| c2_d (required) \| \| --- \| --- \| --- \| --- \| --- \| | How much did you pay for: Transport to get to the clinic (one way)?  Please enter the amount in Tanzanian Shilling. If DK, enter -98. If RF, enter -99.  Question relevant when: selected( ${c2} , '4')  Response constrained to: .>-1 or .=-98 or .=-99 |  |
| \|  \|  \|  \|  \| c2_e (required) \| \| --- \| --- \| --- \| --- \| --- \| | How much did you pay for: Payment for someone to look after your children while you are gone to the clinic?  Please enter the amount in Tanzanian Shilling. If DK, enter -98. If RF, enter -99.  Question relevant when: selected( ${c2} , '5')  Response constrained to: .>-1 or .=-98 or .=-99 |  |
| \|  \|  \|  \|  \| c2_f (required) \| \| --- \| --- \| --- \| --- \| --- \| | How much did you pay for: Food since you started travelling to the clinic until the time of the interview?  Please enter the amount in Tanzanian Shilling. If DK, enter -98. If RF, enter -99.  Question relevant when: selected( ${c2} , '6')  Response constrained to: .>-1 or .=-98 or .=-99 |  |
| \|  \|  \|  \|  \| c2_g (required) \| \| --- \| --- \| --- \| --- \| --- \| | How much did you pay for: Phone calls/SMS since you started travelling to the clinic until the time of the interview  Please enter the amount in Tanzanian Shilling. If DK, enter -98. If RF, enter -99.  Question relevant when: selected( ${c2} , '7')  Response constrained to: .>-1 or .=-98 or .=-99 |  |
| \|  \|  \|  \|  \| c2_h (required) \| \| --- \| --- \| --- \| --- \| --- \| | How much did you pay for: Others?  Please enter the amount in Tanzanian Shilling. If DK, enter -98. If RF, enter -99.  Question relevant when: selected( ${c2} , '8')  Response constrained to: .>-1 or .=-98 or .=-99 |  |
| \|  \|  \|  \|  \| c3 (required) \| \| --- \| --- \| --- \| --- \| --- \| | In the past 12 months, were you admitted to a hospital? With being admitted to a hospital, I mean that you slept in the hospital and did not just come there for a few hours. | \|  \| 1 \| Yes \| \| --- \| --- \| --- \| \|  \| 2 \| No \| \|  \| 99 \| REFUSED \| |
| \|  \|  \|  \|  \| c4 (required) \| \| --- \| --- \| --- \| --- \| --- \| | How many times were you admitted to hospital in the last 12 months?  Please record the number of times. If DON'T KNOW enter '8888.' If REFUSED enter '9999'  Question relevant when: ${c3} =1  Response constrained to: .>-1 |  |
| \|  \|  \|  \|  \| over18_group > hivyes > a5-13_group > hiv_positive > Hospital stay (1)  Group relevant when: ${c4} <366 \| \| --- \| --- \| --- \| --- \| --- \| | | (Repeated group) |
| \|  \|  \|  \|  \|  \| c5_note \| \| --- \| --- \| --- \| --- \| --- \| --- \| | For hospital admission # 1, please write the date, number of nights you spent in hospital, and name of the facility. |  |
| \|  \|  \|  \|  \|  \| c5_a (required) \| \| --- \| --- \| --- \| --- \| --- \| --- \| | Date of hospital admission  If respondent DK, enter January 1st, 1980. If respondent RF, enter February 1st, 1980.  Response constrained to: . <= today() |  |
| \|  \|  \|  \|  \|  \| c5_b (required) \| \| --- \| --- \| --- \| --- \| --- \| --- \| | Number of nights spent in the hospital  If respondent DK, enter '8888'. If respondent RF, enter '9999'. |  |
| \|  \|  \|  \|  \|  \| c5_c (required) \| \| --- \| --- \| --- \| --- \| --- \| --- \| | Name of the facility |  |
| \|  \|  \|  \|  \|  \| c5_noteb \| \| --- \| --- \| --- \| --- \| --- \| --- \| | For this hospital admission, how much did you pay for:  Please record the answers in TSh. If DK, enter -98. If RF, enter -99. |  |
| \|  \|  \|  \|  \|  \| c5_d1 (required) \| \| --- \| --- \| --- \| --- \| --- \| --- \| | Hospital/consultation fees  Response constrained to: .>-1 or .=-98 or .=-99 |  |
| \|  \|  \|  \|  \|  \| c5_d2 (required) \| \| --- \| --- \| --- \| --- \| --- \| --- \| | Surgery  Response constrained to: .>-1 or .=-98 or .=-99 |  |
| \|  \|  \|  \|  \|  \| c5_d3 (required) \| \| --- \| --- \| --- \| --- \| --- \| --- \| | Medical tests (e.g. blood tests and X-rays)  Response constrained to: .>-1 or .=-98 or .=-99 |  |
| \|  \|  \|  \|  \|  \| c5_d4 (required) \| \| --- \| --- \| --- \| --- \| --- \| --- \| | Medicines  Response constrained to: .>-1 or .=-98 or .=-99 |  |
| \|  \|  \|  \|  \|  \| c5_d5 (required) \| \| --- \| --- \| --- \| --- \| --- \| --- \| | Other hospital fees  Response constrained to: .>-1 or .=-98 or .=-99 |  |
| \|  \|  \|  \|  \|  \| c5_d6 (required) \| \| --- \| --- \| --- \| --- \| --- \| --- \| | Transport including ambulance charges  Response constrained to: .>-1 or .=-98 or .=-99 |  |
| \|  \|  \|  \|  \|  \| c5_d7 (required) \| \| --- \| --- \| --- \| --- \| --- \| --- \| | Payment for someone to look after your child while you were in hospital  Response constrained to: .>-1 or .=-98 or .=-99 |  |
| \|  \|  \|  \|  \|  \| c5_d8 (required) \| \| --- \| --- \| --- \| --- \| --- \| --- \| | Payment for someone to look after your house, garden/fields or animals while you were in hospital  Response constrained to: .>-1 or .=-98 or .=-99 |  |
| \|  \|  \|  \|  \|  \| c5_d9 (required) \| \| --- \| --- \| --- \| --- \| --- \| --- \| | Food  Response constrained to: .>-1 or .=-98 or .=-99 |  |
| \|  \|  \|  \|  \|  \| c5_d10 (required) \| \| --- \| --- \| --- \| --- \| --- \| --- \| | Phone calls/SMS  Response constrained to: .>-1 or .=-98 or .=-99 |  |
| \|  \|  \|  \|  \|  \| c5_d11 (required) \| \| --- \| --- \| --- \| --- \| --- \| --- \| | Accommodation if you needed to stay the night nearby  Response constrained to: .>-1 or .=-98 or .=-99 |  |
| \|  \|  \|  \|  \|  \| c5_d12 (required) \| \| --- \| --- \| --- \| --- \| --- \| --- \| | Other (SPECIFY BELOW)  Response constrained to: .>-1 or .=-98 or .=-99 |  |
| \|  \|  \|  \|  \|  \| c5_d12other \| \| --- \| --- \| --- \| --- \| --- \| --- \| | Please specify 'Other'. |  |
| \|  \|  \|  \|  \| c6_label \| \| --- \| --- \| --- \| --- \| --- \| | The following questions ask about healthcare you accessed in the past SIX months. |  |
| \|  \|  \|  \|  \| over18_group > hivyes > a5-13_group > hiv_positive > c6 \| \| --- \| --- \| --- \| --- \| --- \| | | |
| \|  \|  \|  \|  \|  \| c6_note \| \| --- \| --- \| --- \| --- \| --- \| --- \| | In the past SIX MONTHS, have you visited… | \|  \| 1 \| Yes \| \| --- \| --- \| --- \| \|  \| 2 \| No \| \|  \| 99 \| REFUSED \| |
| \|  \|  \|  \|  \|  \| c6_a (required) \| \| --- \| --- \| --- \| --- \| --- \| --- \| | A public primary care clinic | \|  \| 1 \| Yes \| \| --- \| --- \| --- \| \|  \| 2 \| No \| \|  \| 99 \| REFUSED \| |
| \|  \|  \|  \|  \|  \| c6_b (required) \| \| --- \| --- \| --- \| --- \| --- \| --- \| | A private doctor | \|  \| 1 \| Yes \| \| --- \| --- \| --- \| \|  \| 2 \| No \| \|  \| 99 \| REFUSED \| |
| \|  \|  \|  \|  \|  \| c6_c (required) \| \| --- \| --- \| --- \| --- \| --- \| --- \| | Chemist / pharmacy | \|  \| 1 \| Yes \| \| --- \| --- \| --- \| \|  \| 2 \| No \| \|  \| 99 \| REFUSED \| |
| \|  \|  \|  \|  \|  \| c6_d (required) \| \| --- \| --- \| --- \| --- \| --- \| --- \| | Traditional healer | \|  \| 1 \| Yes \| \| --- \| --- \| --- \| \|  \| 2 \| No \| \|  \| 99 \| REFUSED \| |
| \|  \|  \|  \|  \|  \| c6_e (required) \| \| --- \| --- \| --- \| --- \| --- \| --- \| | Diviner | \|  \| 1 \| Yes \| \| --- \| --- \| --- \| \|  \| 2 \| No \| \|  \| 99 \| REFUSED \| |
| \|  \|  \|  \|  \|  \| c6_f (required) \| \| --- \| --- \| --- \| --- \| --- \| --- \| | A faith healer | \|  \| 1 \| Yes \| \| --- \| --- \| --- \| \|  \| 2 \| No \| \|  \| 99 \| REFUSED \| |
| \|  \|  \|  \|  \| c6_a1 (required) \| \| --- \| --- \| --- \| --- \| --- \| | In the past SIX MONTHS, how many times did you visit: a public primary care clinic?  Question relevant when: ${c6_a} =1  Response constrained to: .>-1 |  |
| \|  \|  \|  \|  \| c6_error \| \| --- \| --- \| --- \| --- \| --- \| | NOTE: YOU INPUT 0 VISITS, BUT IN A PREVIOUS QUESTION YOU INPUT THAT THE RESPONDENT VISITED THIS FACILITY / HEALER IN THE PAST MONTH. PLEASE GO BACK AND CONFIRM THE RESPONSES.  Question relevant when: ${c6_a1} =0 |  |
| \|  \|  \|  \|  \| over18_group > hivyes > a5-13_group > hiv_positive > c6_1  Group relevant when: ${c6_a} =1 \| \| --- \| --- \| --- \| --- \| --- \| | | |
| \|  \|  \|  \|  \|  \| c6_a1label \| \| --- \| --- \| --- \| --- \| --- \| --- \| | For the last public primary care clinic visit, how much did you pay for:  Please record the answers in TSh. If DK, enter -98. If RF, enter -99. |  |
| \|  \|  \|  \|  \|  \| c6_a2 (required) \| \| --- \| --- \| --- \| --- \| --- \| --- \| | Clinic/consultation fee  Response constrained to: .>-1 or .=-98 or .=-99 |  |
| \|  \|  \|  \|  \|  \| c6_a3 (required) \| \| --- \| --- \| --- \| --- \| --- \| --- \| | Medical tests (e.g. blood tests and X-rays)  Response constrained to: .>-1 or .=-98 or .=-99 |  |
| \|  \|  \|  \|  \|  \| c6_a4 (required) \| \| --- \| --- \| --- \| --- \| --- \| --- \| | Medicines  Response constrained to: .>-1 or .=-98 or .=-99 |  |
| \|  \|  \|  \|  \|  \| c6_a5 (required) \| \| --- \| --- \| --- \| --- \| --- \| --- \| | Transport  Response constrained to: .>-1 or .=-98 or .=-99 |  |
| \|  \|  \|  \|  \|  \| c6_a6 (required) \| \| --- \| --- \| --- \| --- \| --- \| --- \| | Payment for someone to look after your child while you were gone  Response constrained to: .>-1 or .=-98 or .=-99 |  |
| \|  \|  \|  \|  \|  \| c6_a7 (required) \| \| --- \| --- \| --- \| --- \| --- \| --- \| | Payment for someone to look after your house, garden/fields or animals while you were gone  Response constrained to: .>-1 or .=-98 or .=-99 |  |
| \|  \|  \|  \|  \|  \| c6_a8 (required) \| \| --- \| --- \| --- \| --- \| --- \| --- \| | Food  Response constrained to: .>-1 or .=-98 or .=-99 |  |
| \|  \|  \|  \|  \|  \| c6_a9 (required) \| \| --- \| --- \| --- \| --- \| --- \| --- \| | Phone calls/SMS  Response constrained to: .>-1 or .=-98 or .=-99 |  |
| \|  \|  \|  \|  \|  \| c6_a10 (required) \| \| --- \| --- \| --- \| --- \| --- \| --- \| | Accommodation if you needed to stay the night nearby  Response constrained to: .>-1 or .=-98 or .=-99 |  |
| \|  \|  \|  \|  \|  \| c6_a11 (required) \| \| --- \| --- \| --- \| --- \| --- \| --- \| | Other (SPECIFY ON THE NEXT PAGE)  Response constrained to: .>-1 or .=-98 or .=-99 |  |
| \|  \|  \|  \|  \| c6_a11_other (required) \| \| --- \| --- \| --- \| --- \| --- \| | Please specify 'Other' payment related to primary care clinic visit(s)  Question relevant when: ${c6_a11} >0 |  |
| \|  \|  \|  \|  \| c6_b1 (required) \| \| --- \| --- \| --- \| --- \| --- \| | In the past SIX MONTHS, how many times did you visit: a private doctor?  Question relevant when: ${c6_b} =1  Response constrained to: .>-1 |  |
| \|  \|  \|  \|  \| c6_errorb \| \| --- \| --- \| --- \| --- \| --- \| | NOTE: YOU INPUT 0 VISITS, BUT IN A PREVIOUS QUESTION YOU INPUT THAT THE RESPONDENT VISITED THIS FACILITY / HEALER IN THE PAST MONTH. PLEASE GO BACK AND CONFIRM THE RESPONSES.  Question relevant when: ${c6_b1} =0 |  |
| \|  \|  \|  \|  \| over18_group > hivyes > a5-13_group > hiv_positive > c6_3  Group relevant when: ${c6_b} =1 \| \| --- \| --- \| --- \| --- \| --- \| | | |
| \|  \|  \|  \|  \|  \| c6_b1label \| \| --- \| --- \| --- \| --- \| --- \| --- \| | For the last private doctor visit, how much did you spend for:  Please record the answers in TSh. If DK, enter -98. If RF, enter -99. |  |
| \|  \|  \|  \|  \|  \| c6_b2 (required) \| \| --- \| --- \| --- \| --- \| --- \| --- \| | Clinic/consultation fee  Response constrained to: .>-1 or .=-98 or .=-99 |  |
| \|  \|  \|  \|  \|  \| c6_b3 (required) \| \| --- \| --- \| --- \| --- \| --- \| --- \| | Medical tests (e.g. blood tests and X-rays)  Response constrained to: .>-1 or .=-98 or .=-99 |  |
| \|  \|  \|  \|  \|  \| c6_b4 (required) \| \| --- \| --- \| --- \| --- \| --- \| --- \| | Medicines  Response constrained to: .>-1 or .=-98 or .=-99 |  |
| \|  \|  \|  \|  \|  \| c6_b5 (required) \| \| --- \| --- \| --- \| --- \| --- \| --- \| | Transport  Response constrained to: .>-1 or .=-98 or .=-99 |  |
| \|  \|  \|  \|  \|  \| c6_b6 (required) \| \| --- \| --- \| --- \| --- \| --- \| --- \| | Payment for someone to look after your child while you were gone  Response constrained to: .>-1 or .=-98 or .=-99 |  |
| \|  \|  \|  \|  \|  \| c6_b7 (required) \| \| --- \| --- \| --- \| --- \| --- \| --- \| | Payment for someone to look after your house, garden/fields or animals while you were gone  Response constrained to: .>-1 or .=-98 or .=-99 |  |
| \|  \|  \|  \|  \|  \| c6_b8 (required) \| \| --- \| --- \| --- \| --- \| --- \| --- \| | Food  Response constrained to: .>-1 or .=-98 or .=-99 |  |
| \|  \|  \|  \|  \|  \| c6_b9 (required) \| \| --- \| --- \| --- \| --- \| --- \| --- \| | Phone calls/SMS  Response constrained to: .>-1 or .=-98 or .=-99 |  |
| \|  \|  \|  \|  \|  \| c6_b10 (required) \| \| --- \| --- \| --- \| --- \| --- \| --- \| | Accommodation if you needed to stay the night nearby  Response constrained to: .>-1 or .=-98 or .=-99 |  |
| \|  \|  \|  \|  \|  \| c6_b11 (required) \| \| --- \| --- \| --- \| --- \| --- \| --- \| | Other (specify below)  Response constrained to: .>-1 or .=-98 or .=-99 |  |
| \|  \|  \|  \|  \| c6_b11_other (required) \| \| --- \| --- \| --- \| --- \| --- \| | Please specify 'Other' payment related to private doctor visit(s)  Question relevant when: ${c6_b11} >0 |  |
| \|  \|  \|  \|  \| c6_c1 (required) \| \| --- \| --- \| --- \| --- \| --- \| | In the past SIX MONTHS, how many times did you visit: a chemist / pharmacy?  Question relevant when: ${c6_c} =1  Response constrained to: .>-1 |  |
| \|  \|  \|  \|  \| c6_errorc \| \| --- \| --- \| --- \| --- \| --- \| | NOTE: YOU INPUT 0 VISITS, BUT IN A PREVIOUS QUESTION YOU INPUT THAT THE RESPONDENT VISITED THIS FACILITY / HEALER IN THE PAST MONTH. PLEASE GO BACK AND CONFIRM THE RESPONSES.  Question relevant when: ${c6_c1} =0 |  |
| \|  \|  \|  \|  \| over18_group > hivyes > a5-13_group > hiv_positive > c6_4  Group relevant when: ${c6_c} =1 \| \| --- \| --- \| --- \| --- \| --- \| | | |
| \|  \|  \|  \|  \|  \| c6_c1label \| \| --- \| --- \| --- \| --- \| --- \| --- \| | For the last Chemist / pharmacy visit, how much did you spend for:  Please record the answers in TSh. If DK, enter -98. If RF, enter -99. |  |
| \|  \|  \|  \|  \|  \| c6_c2 (required) \| \| --- \| --- \| --- \| --- \| --- \| --- \| | Clinic/consultation fee  Response constrained to: .>-1 or .=-98 or .=-99 |  |
| \|  \|  \|  \|  \|  \| c6_c3 (required) \| \| --- \| --- \| --- \| --- \| --- \| --- \| | Medical tests (e.g. blood tests and X-rays)  Response constrained to: .>-1 or .=-98 or .=-99 |  |
| \|  \|  \|  \|  \|  \| c6_c4 (required) \| \| --- \| --- \| --- \| --- \| --- \| --- \| | Medicines  Response constrained to: .>-1 or .=-98 or .=-99 |  |
| \|  \|  \|  \|  \|  \| c6_c5 (required) \| \| --- \| --- \| --- \| --- \| --- \| --- \| | Transport  Response constrained to: .>-1 or .=-98 or .=-99 |  |
| \|  \|  \|  \|  \|  \| c6_c6 (required) \| \| --- \| --- \| --- \| --- \| --- \| --- \| | Payment for someone to look after your child while you were gone  Response constrained to: .>-1 or .=-98 or .=-99 |  |
| \|  \|  \|  \|  \|  \| c6_c7 (required) \| \| --- \| --- \| --- \| --- \| --- \| --- \| | Payment for someone to look after your house, garden/fields or animals while you were gone  Response constrained to: .>-1 or .=-98 or .=-99 |  |
| \|  \|  \|  \|  \|  \| c6_c8 (required) \| \| --- \| --- \| --- \| --- \| --- \| --- \| | Food  Response constrained to: .>-1 or .=-98 or .=-99 |  |
| \|  \|  \|  \|  \|  \| c6_c9 (required) \| \| --- \| --- \| --- \| --- \| --- \| --- \| | Phone calls/SMS  Response constrained to: .>-1 or .=-98 or .=-99 |  |
| \|  \|  \|  \|  \|  \| c6_c10 (required) \| \| --- \| --- \| --- \| --- \| --- \| --- \| | Accommodation if you needed to stay the night nearby  Response constrained to: .>-1 or .=-98 or .=-99 |  |
| \|  \|  \|  \|  \|  \| c6_c11 (required) \| \| --- \| --- \| --- \| --- \| --- \| --- \| | Other (specify below)  Response constrained to: .>-1 or .=-98 or .=-99 |  |
| \|  \|  \|  \|  \| c6_c11_other (required) \| \| --- \| --- \| --- \| --- \| --- \| | Please specify 'Other' payment related to Chemist / pharmacy(s)  Question relevant when: ${c6_c11} >0 |  |
| \|  \|  \|  \|  \| c6_d1 (required) \| \| --- \| --- \| --- \| --- \| --- \| | In the past SIX MONTHS, how many times did you visit: a traditional healer?  Question relevant when: ${c6_d} =1  Response constrained to: .>-1 |  |
| \|  \|  \|  \|  \| c6_errord \| \| --- \| --- \| --- \| --- \| --- \| | NOTE: YOU INPUT 0 VISITS, BUT IN A PREVIOUS QUESTION YOU INPUT THAT THE RESPONDENT VISITED THIS FACILITY / HEALER IN THE PAST MONTH. PLEASE GO BACK AND CONFIRM THE RESPONSES.  Question relevant when: ${c6_d1} =0 |  |
| \|  \|  \|  \|  \| over18_group > hivyes > a5-13_group > hiv_positive > c6_5  Group relevant when: ${c6_d} =1 \| \| --- \| --- \| --- \| --- \| --- \| | | |
| \|  \|  \|  \|  \|  \| c6_d1label \| \| --- \| --- \| --- \| --- \| --- \| --- \| | For the last Traditional healer visit, how much did you spend for:  Please record the answers in TSh. If DK, enter -98. If RF, enter -99. |  |
| \|  \|  \|  \|  \|  \| c6_d2 (required) \| \| --- \| --- \| --- \| --- \| --- \| --- \| | Clinic/consultation fee  Response constrained to: .>-1 or .=-98 or .=-99 |  |
| \|  \|  \|  \|  \|  \| c6_d3 (required) \| \| --- \| --- \| --- \| --- \| --- \| --- \| | Medical tests (e.g. blood tests and X-rays)  Response constrained to: .>-1 or .=-98 or .=-99 |  |
| \|  \|  \|  \|  \|  \| c6_d4 (required) \| \| --- \| --- \| --- \| --- \| --- \| --- \| | Medicines  Response constrained to: .>-1 or .=-98 or .=-99 |  |
| \|  \|  \|  \|  \|  \| c6_d5 (required) \| \| --- \| --- \| --- \| --- \| --- \| --- \| | Transport  Response constrained to: .>-1 or .=-98 or .=-99 |  |
| \|  \|  \|  \|  \|  \| c6_d6 (required) \| \| --- \| --- \| --- \| --- \| --- \| --- \| | Payment for someone to look after your child while you were gone  Response constrained to: .>-1 or .=-98 or .=-99 |  |
| \|  \|  \|  \|  \|  \| c6_d7 (required) \| \| --- \| --- \| --- \| --- \| --- \| --- \| | Payment for someone to look after your house, garden/fields or animals while you were gone  Response constrained to: .>-1 or .=-98 or .=-99 |  |
| \|  \|  \|  \|  \|  \| c6_d8 (required) \| \| --- \| --- \| --- \| --- \| --- \| --- \| | Food  Response constrained to: .>-1 or .=-98 or .=-99 |  |
| \|  \|  \|  \|  \|  \| c6_d9 (required) \| \| --- \| --- \| --- \| --- \| --- \| --- \| | Phone calls/SMS  Response constrained to: .>-1 or .=-98 or .=-99 |  |
| \|  \|  \|  \|  \|  \| c6_d10 (required) \| \| --- \| --- \| --- \| --- \| --- \| --- \| | Accommodation if you needed to stay the night nearby  Response constrained to: .>-1 or .=-98 or .=-99 |  |
| \|  \|  \|  \|  \|  \| c6_d11 (required) \| \| --- \| --- \| --- \| --- \| --- \| --- \| | Other (SPECIFY ON THE NEXT PAGE)  Response constrained to: .>-1 or .=-98 or .=-99 |  |
| \|  \|  \|  \|  \| c6_d11_other (required) \| \| --- \| --- \| --- \| --- \| --- \| | Please specify 'Other' payment related to Traditional healer visit(s)  Question relevant when: ${c6_d11} >0 |  |
| \|  \|  \|  \|  \| c6_e1 (required) \| \| --- \| --- \| --- \| --- \| --- \| | In the past SIX MONTHS, how many times did you visit: a diviner?  Question relevant when: ${c6_e} =1  Response constrained to: .>-1 |  |
| \|  \|  \|  \|  \| c6_errore \| \| --- \| --- \| --- \| --- \| --- \| | NOTE: YOU INPUT 0 VISITS, BUT IN A PREVIOUS QUESTION YOU INPUT THAT THE RESPONDENT VISITED THIS FACILITY / HEALER IN THE PAST MONTH. PLEASE GO BACK AND CONFIRM THE RESPONSES.  Question relevant when: ${c6_e1} =0 |  |
| \|  \|  \|  \|  \| over18_group > hivyes > a5-13_group > hiv_positive > c6_6  Group relevant when: ${c6_e} =1 \| \| --- \| --- \| --- \| --- \| --- \| | | |
| \|  \|  \|  \|  \|  \| c6_e1label \| \| --- \| --- \| --- \| --- \| --- \| --- \| | For the last Diviner visit, how much did you spend for:  Please record the answers in TSh. If DK, enter -98. If RF, enter -99. |  |
| \|  \|  \|  \|  \|  \| c6_e2 (required) \| \| --- \| --- \| --- \| --- \| --- \| --- \| | Clinic/consultation fee  Response constrained to: .>-1 or .=-98 or .=-99 |  |
| \|  \|  \|  \|  \|  \| c6_e3 (required) \| \| --- \| --- \| --- \| --- \| --- \| --- \| | Medical tests (e.g. blood tests and X-rays)  Response constrained to: .>-1 or .=-98 or .=-99 |  |
| \|  \|  \|  \|  \|  \| c6_e4 (required) \| \| --- \| --- \| --- \| --- \| --- \| --- \| | Medicines  Response constrained to: .>-1 or .=-98 or .=-99 |  |
| \|  \|  \|  \|  \|  \| c6_e5 (required) \| \| --- \| --- \| --- \| --- \| --- \| --- \| | Transport  Response constrained to: .>-1 or .=-98 or .=-99 |  |
| \|  \|  \|  \|  \|  \| c6_e6 (required) \| \| --- \| --- \| --- \| --- \| --- \| --- \| | Payment for someone to look after your child while you were gone  Response constrained to: .>-1 or .=-98 or .=-99 |  |
| \|  \|  \|  \|  \|  \| c6_e7 (required) \| \| --- \| --- \| --- \| --- \| --- \| --- \| | Payment for someone to look after your house, garden/fields or animals while you were gone  Response constrained to: .>-1 or .=-98 or .=-99 |  |
| \|  \|  \|  \|  \|  \| c6_e8 (required) \| \| --- \| --- \| --- \| --- \| --- \| --- \| | Food  Response constrained to: .>-1 or .=-98 or .=-99 |  |
| \|  \|  \|  \|  \|  \| c6_e9 (required) \| \| --- \| --- \| --- \| --- \| --- \| --- \| | Phone calls/SMS  Response constrained to: .>-1 or .=-98 or .=-99 |  |
| \|  \|  \|  \|  \|  \| c6_e10 (required) \| \| --- \| --- \| --- \| --- \| --- \| --- \| | Accommodation if you needed to stay the night nearby  Response constrained to: .>-1 or .=-98 or .=-99 |  |
| \|  \|  \|  \|  \|  \| c6_e11 (required) \| \| --- \| --- \| --- \| --- \| --- \| --- \| | Other (specify below)  Response constrained to: .>-1 or .=-98 or .=-99 |  |
| \|  \|  \|  \|  \| c6_e11_other (required) \| \| --- \| --- \| --- \| --- \| --- \| | Please specify 'Other' payment related to Diviner visit(s)  Question relevant when: ${c6_e11} >0 |  |
| \|  \|  \|  \|  \| c6_f1 (required) \| \| --- \| --- \| --- \| --- \| --- \| | In the past SIX MONTHS, how many times did you visit: a faith healer?  Question relevant when: ${c6_f} =1  Response constrained to: .>-1 |  |
| \|  \|  \|  \|  \| c6_errorf \| \| --- \| --- \| --- \| --- \| --- \| | NOTE: YOU INPUT 0 VISITS, BUT IN A PREVIOUS QUESTION YOU INPUT THAT THE RESPONDENT VISITED THIS FACILITY / HEALER IN THE PAST MONTH. PLEASE GO BACK AND CONFIRM THE RESPONSES.  Question relevant when: ${c6_f1} =0 |  |
| \|  \|  \|  \|  \| over18_group > hivyes > a5-13_group > hiv_positive > c6_7  Group relevant when: ${c6_f} =1 \| \| --- \| --- \| --- \| --- \| --- \| | | |
| \|  \|  \|  \|  \|  \| c6_f1label \| \| --- \| --- \| --- \| --- \| --- \| --- \| | For the last Faith healer visit, how much did you spend for:  Please record the answers in TSh. If DK, enter -98. If RF, enter -99. |  |
| \|  \|  \|  \|  \|  \| c6_f2 (required) \| \| --- \| --- \| --- \| --- \| --- \| --- \| | Clinic/consultation fee  Response constrained to: .>-1 or .=-98 or .=-99 |  |
| \|  \|  \|  \|  \|  \| c6_f3 (required) \| \| --- \| --- \| --- \| --- \| --- \| --- \| | Medical tests (e.g. blood tests and X-rays)  Response constrained to: .>-1 or .=-98 or .=-99 |  |
| \|  \|  \|  \|  \|  \| c6_f4 (required) \| \| --- \| --- \| --- \| --- \| --- \| --- \| | Medicines  Response constrained to: .>-1 or .=-98 or .=-99 |  |
| \|  \|  \|  \|  \|  \| c6_f5 (required) \| \| --- \| --- \| --- \| --- \| --- \| --- \| | Transport  Response constrained to: .>-1 or .=-98 or .=-99 |  |
| \|  \|  \|  \|  \|  \| c6_f6 (required) \| \| --- \| --- \| --- \| --- \| --- \| --- \| | Payment for someone to look after your child while you were gone  Response constrained to: .>-1 or .=-98 or .=-99 |  |
| \|  \|  \|  \|  \|  \| c6_f7 (required) \| \| --- \| --- \| --- \| --- \| --- \| --- \| | Payment for someone to look after your house, garden/fields or animals while you were gone  Response constrained to: .>-1 or .=-98 or .=-99 |  |
| \|  \|  \|  \|  \|  \| c6_f8 (required) \| \| --- \| --- \| --- \| --- \| --- \| --- \| | Food  Response constrained to: .>-1 or .=-98 or .=-99 |  |
| \|  \|  \|  \|  \|  \| c6_f9 (required) \| \| --- \| --- \| --- \| --- \| --- \| --- \| | Phone calls/SMS  Response constrained to: .>-1 or .=-98 or .=-99 |  |
| \|  \|  \|  \|  \|  \| c6_f10 (required) \| \| --- \| --- \| --- \| --- \| --- \| --- \| | Accommodation if you needed to stay the night nearby  Response constrained to: .>-1 or .=-98 or .=-99 |  |
| \|  \|  \|  \|  \|  \| c6_f11 (required) \| \| --- \| --- \| --- \| --- \| --- \| --- \| | Other (specify below)  Response constrained to: .>-1 or .=-98 or .=-99 |  |
| \|  \|  \|  \|  \| c6_f11_other (required) \| \| --- \| --- \| --- \| --- \| --- \| | Please specify 'Other' payment related to faith healer visit(s)  Question relevant when: ${c6_f11} >0 |  |
| \|  \|  \|  \|  \| c126 (required) \| \| --- \| --- \| --- \| --- \| --- \| | How much did you spend on other health care in the past SIX months, such as traditional medicines, medicines from a convenience store, special food, etc.?  Please record the amount in TSh. If DK, enter -98. If RF, enter -99.  Response constrained to: .>-1 or .=-98 or .=-99 |  |
| \|  \|  \|  \|  \| c127 (required) \| \| --- \| --- \| --- \| --- \| --- \| | In the past six months did you have to borrow money to pay for healthcare? | \|  \| 1 \| Yes \| \| --- \| --- \| --- \| \|  \| 2 \| No \| \|  \| 99 \| REFUSED \| |
| \|  \|  \|  \|  \| c128 (required) \| \| --- \| --- \| --- \| --- \| --- \| | How much money did you borrow?  Please record the amount in TSh. If DK, enter -98. If RF, enter -99.  Question relevant when: ${c127} =1  Response constrained to: .>-1 or .=-98 or .=-99 |  |
| \|  \|  \|  \|  \| c129 (required) \| \| --- \| --- \| --- \| --- \| --- \| | In the past six months did you have to sell personal or household items in order to pay for healthcare? | \|  \| 1 \| Yes \| \| --- \| --- \| --- \| \|  \| 2 \| No \| \|  \| 99 \| REFUSED \| |
| \|  \|  \|  \|  \| c130 (required) \| \| --- \| --- \| --- \| --- \| --- \| | At what time did you arrive at the clinic today?  Please enter the time. |  |
| \|  \|  \|  \|  \| over18_group > hivyes > a5-13_group > hiv_positive > time_clinic \| \| --- \| --- \| --- \| --- \| --- \| | | |
| \|  \|  \|  \|  \|  \| c131 \| \| --- \| --- \| --- \| --- \| --- \| --- \| | How much time did it take you today to get to the clinic?  For example, if it took the respondent 1 hour and 30 minutes to get to the clinic, input '1' HOUR and '30' MINUTES. If respondent does not know, enter '98' HOURS and '98' MINUTES. If respondent refuses, enter '99' HOURS and '99' MINUTES. |  |
| \|  \|  \|  \|  \|  \| c131_hr (required) \| \| --- \| --- \| --- \| --- \| --- \| --- \| | HOURS: |  |
| \|  \|  \|  \|  \|  \| c131_min (required) \| \| --- \| --- \| --- \| --- \| --- \| --- \| | MINUTES: |  |
| \|  \|  \|  \|  \| c132 (required) \| \| --- \| --- \| --- \| --- \| --- \| | Approximately how many minutes did you spend waiting to be seen by a nurse or physician today?  Please enter the number of minutes. If DK, enter -98. If RF, enter -99. |  |
| \|  \|  \|  \|  \| c133 (required) \| \| --- \| --- \| --- \| --- \| --- \| | Approximately how many minutes did you spend with the nurse or physician today?  Please enter the number of minutes. If DK, enter -98. If RF, enter -99. |  |
| \|  \|  \|  \|  \| over18_group > hivyes > a5-13_group > hiv_positive > c134_group \| \| --- \| --- \| --- \| --- \| --- \| | | |
| \|  \|  \|  \|  \|  \| c134 (required) \| \| --- \| --- \| --- \| --- \| --- \| --- \| | What would you have been doing if you had not gone to the ART clinic today?  Read out each option and select all that apply.  Response constrained to: if(selected(., '99'), count-selected(.)=1, count-selected(.)>=1) and if(selected(., '7'), count-selected(.)=1, count-selected(.)>=1) | \|  \| 1 \| Earning money \| \| --- \| --- \| --- \| \|  \| 2 \| Doing unpaid community work or volunteer work \| \|  \| 3 \| Doing household chores such as cleaning, cooking, shopping for food, maintenance and repairs, working in the garden, gathering wood, gathering water, housework, etc \| \|  \| 4 \| Taking care of children \| \|  \| 5 \| Leisure activities (sport, watching TV, listening to music, reading, visiting friends and family, going to movies, etc.) \| \|  \| 6 \| Attending school or other educational institution \| \|  \| 7 \| Nothing \| \|  \| 8 \| Other \| \|  \| 99 \| REFUSED \| |
| \|  \|  \|  \|  \|  \| c134_other \| \| --- \| --- \| --- \| --- \| --- \| --- \| | Specify if 'Other':  Question relevant when: selected( ${c134} , 8) |  |
| \|  \|  \|  \|  \| c135 (required) \| \| --- \| --- \| --- \| --- \| --- \| | Did you lose any money from the time you took to come to the clinic?  Question relevant when: selected( ${c134} , '1') | \|  \| 1 \| Yes \| \| --- \| --- \| --- \| \|  \| 2 \| No \| \|  \| 99 \| REFUSED \| |
| \|  \|  \|  \|  \| c136 (required) \| \| --- \| --- \| --- \| --- \| --- \| | How much money did you lose?  Please record the answers in TSh. If DK, enter -98. If RF, enter -99.  Question relevant when: ${c135} =1  Response constrained to: .>-1 or .=-98 or .=-99 |  |
| \|  \|  \|  \|  \| note_d \| \| --- \| --- \| --- \| --- \| --- \| | PART 4: SELF-REPORTED ART ADHERENCE |  |
| \|  \|  \|  \|  \| d1 (required) \| \| --- \| --- \| --- \| --- \| --- \| | Have you ever informed anyone about your HIV status? | \|  \| 1 \| Yes \| \| --- \| --- \| --- \| \|  \| 0 \| No \| \|  \| 2 \| DON'T KNOW \| \|  \| 3 \| REFUSED \| |
| \|  \|  \|  \|  \| over18_group > hivyes > a5-13_group > hiv_positive > disclosure \| \| --- \| --- \| --- \| --- \| --- \| | | |
| \|  \|  \|  \|  \|  \| d2 (required) \| \| --- \| --- \| --- \| --- \| --- \| --- \| | To whom did you inform about your HIV status?  Read out each option and select all that apply. Probe with: Anyone else?  Question relevant when: ${d1} =1  Response constrained to: if(selected(., '99'), count-selected(.)=1, count-selected(.)>=1) | \|  \| 1 \| Spouse / Current partner \| \| --- \| --- \| --- \| \|  \| 2 \| Girlfriend / Boyfriend \| \|  \| 3 \| Parent \| \|  \| 4 \| Brother / Sister \| \|  \| 5 \| Someone else in the family \| \|  \| 6 \| Friend \| \|  \| 7 \| Religious Leader \| \|  \| 99 \| REFUSED \| \|  \| 97 \| Other (specify below) \| |
| \|  \|  \|  \|  \| d2_other (required) \| \| --- \| --- \| --- \| --- \| --- \| | You selected 'Other', please specify:  Question relevant when: selected( ${d2} , 97) |  |
| \|  \|  \|  \|  \| d3 (required) \| \| --- \| --- \| --- \| --- \| --- \| | In total, to how many people have you disclosed your HIV status?  Please enter the number of people.  Question relevant when: ${d1} =1 |  |
| \|  \|  \|  \|  \| d4 (required) \| \| --- \| --- \| --- \| --- \| --- \| | Are you currently taking ARVs? | \|  \| 1 \| Yes \| \| --- \| --- \| --- \| \|  \| 2 \| No \| \|  \| 99 \| REFUSED \| |
| \|  \|  \|  \|  \| over18_group > hivyes > a5-13_group > hiv_positive > yes_ARVs  Group relevant when: ${d4} =1 \| \| --- \| --- \| --- \| --- \| --- \| | | |
| \|  \|  \|  \|  \|  \| d5 (required) \| \| --- \| --- \| --- \| --- \| --- \| --- \| | How would you rate your adherence to ARVs over the last month? Please answer with very poor, poor, fair, good, very good, or excellent. | \|  \| 1 \| Very poor \| \| --- \| --- \| --- \| \|  \| 2 \| Poor \| \|  \| 3 \| Fair \| \|  \| 4 \| Good \| \|  \| 5 \| Very good \| \|  \| 6 \| Excellent \| \|  \| 98 \| DON'T KNOW \| \|  \| 99 \| REFUSED \| |
| \|  \|  \|  \|  \|  \| d6-10 \| \| --- \| --- \| --- \| --- \| --- \| --- \| | Please answer the next five questions with ‘very often’, ‘often’, ‘sometimes’ ‘rarely’, or ‘never’. |  |
| \|  \|  \|  \|  \|  \| over18_group > hivyes > a5-13_group > hiv_positive > yes_ARVs > often \| \| --- \| --- \| --- \| --- \| --- \| --- \| | | |
| \|  \|  \|  \|  \|  \|  \| d6 (required) \| \| --- \| --- \| --- \| --- \| --- \| --- \| --- \| | Some people forget to take their ARVs. In the last one month, how often did this happen to you? | \|  \| 1 \| Very often \| \| --- \| --- \| --- \| \|  \| 2 \| Often \| \|  \| 3 \| Sometimes \| \|  \| 4 \| Rarely \| \|  \| 5 \| Never \| \|  \| 98 \| DON'T KNOW \| \|  \| 99 \| REFUSED \| |
| \|  \|  \|  \|  \|  \|  \| d7 (required) \| \| --- \| --- \| --- \| --- \| --- \| --- \| --- \| | Some people miss out a dose of their ARVs or adjust it to suit their own needs. In the last one month, how often did you do this? | \|  \| 1 \| Very often \| \| --- \| --- \| --- \| \|  \| 2 \| Often \| \|  \| 3 \| Sometimes \| \|  \| 4 \| Rarely \| \|  \| 5 \| Never \| \|  \| 98 \| DON'T KNOW \| \|  \| 99 \| REFUSED \| |
| \|  \|  \|  \|  \|  \|  \| d8 (required) \| \| --- \| --- \| --- \| --- \| --- \| --- \| --- \| | Some people stop taking their ARVs when they feel better. In the last one month, how often did you do this? | \|  \| 1 \| Very often \| \| --- \| --- \| --- \| \|  \| 2 \| Often \| \|  \| 3 \| Sometimes \| \|  \| 4 \| Rarely \| \|  \| 5 \| Never \| \|  \| 98 \| DON'T KNOW \| \|  \| 99 \| REFUSED \| |
| \|  \|  \|  \|  \|  \|  \| d9 (required) \| \| --- \| --- \| --- \| --- \| --- \| --- \| --- \| | Some people stop taking their ARVs when they feel worse. In the last one month, how often did you do this? | \|  \| 1 \| Very often \| \| --- \| --- \| --- \| \|  \| 2 \| Often \| \|  \| 3 \| Sometimes \| \|  \| 4 \| Rarely \| \|  \| 5 \| Never \| \|  \| 98 \| DON'T KNOW \| \|  \| 99 \| REFUSED \| |
| \|  \|  \|  \|  \|  \|  \| d10 (required) \| \| --- \| --- \| --- \| --- \| --- \| --- \| --- \| | Some people miss their clinic appointment to pick up their ARVs. In the last six months, how often did you do this? | \|  \| 1 \| Very often \| \| --- \| --- \| --- \| \|  \| 2 \| Often \| \|  \| 3 \| Sometimes \| \|  \| 4 \| Rarely \| \|  \| 5 \| Never \| \|  \| 98 \| DON'T KNOW \| \|  \| 99 \| REFUSED \| |
| \|  \|  \|  \|  \|  \| d11 (required) \| \| --- \| --- \| --- \| --- \| --- \| --- \| | In the last six months, how many times did you miss your appointment to pick up your ARVs? | \|  \| 1 \| One \| \| --- \| --- \| --- \| \|  \| 2 \| Two \| \|  \| 3 \| Three \| \|  \| 4 \| Four \| \|  \| 5 \| Five \| \|  \| 6 \| Six or more \| \|  \| 7 \| None \| \|  \| 98 \| DON'T KNOW \| \|  \| 99 \| REFUSED \| |
| \|  \|  \|  \|  \|  \| d12 (required) \| \| --- \| --- \| --- \| --- \| --- \| --- \| | What was the reason or the reasons for missing the visits?  DO NOT READ THE LIST ALOUD; PROBE WITH: "ANYTHING ELSE?" Select all that apply.  Question relevant when: ${d11} <7 or ${d10} <5  Response constrained to: if(selected(., '99'), count-selected(.)=1, count-selected(.)>=1) and if(selected(., '98'), count-selected(.)=1, count-selected(.)>=1) | \|  \| 1 \| Lack of money \| \| --- \| --- \| --- \| \|  \| 2 \| Lack of time \| \|  \| 3 \| I felt better \| \|  \| 4 \| I could not take time off from work \| \|  \| 5 \| No transport \| \|  \| 6 \| Too ill to travel \| \|  \| 7 \| Other responsibilities \| \|  \| 8 \| The treatment is not effective / does not make me feel better \| \|  \| 9 \| The queues in the facility are too long \| \|  \| 10 \| The staff are rude or uncaring \| \|  \| 11 \| I have had bad experiences with the staff in the past \| \|  \| 97 \| Other (SPECIFY ON THE NEXT PAGE) \| \|  \| 98 \| Don't know \| \|  \| 99 \| Refused \| |
| \|  \|  \|  \|  \|  \| d12_other (required) \| \| --- \| --- \| --- \| --- \| --- \| --- \| | PLEASE SPECIFY 'Other' REASON  Question relevant when: selected( ${d12} , 97) |  |
| \|  \|  \|  \|  \|  \| d13_pre (required) \| \| --- \| --- \| --- \| --- \| --- \| --- \| | Do you know the date when you last picked up your ARVs from the healthcare facility? | \|  \| 1 \| Yes, I know the date \| \| --- \| --- \| --- \| \|  \| 2 \| No, I do not know the date \| \|  \| 97 \| Never picked up ARVs \| |
| \|  \|  \|  \|  \|  \| d13 (required) \| \| --- \| --- \| --- \| --- \| --- \| --- \| | Before today, when did you last pick up your ARVs from the healthcare facility?  Please enter the date. If If RF, enter Jan 1, 1980.  Question relevant when: ${d13_pre} =1  Response constrained to: . <= today() |  |
| \|  \|  \|  \|  \|  \| d13_dk (required) \| \| --- \| --- \| --- \| --- \| --- \| --- \| | DOES THE RESPONDENT KNOW HOW MANY WEEKS OR DAYS AGO HE/SHE PICKED UP THE ARVS?  Question relevant when: ${d13_pre} =2 | \|  \| 1 \| Weeks \| \| --- \| --- \| --- \| \|  \| 2 \| Days \| \|  \| 98 \| Still don't know \| |
| \|  \|  \|  \|  \|  \| d13_dk_weeks (required) \| \| --- \| --- \| --- \| --- \| --- \| --- \| | How many weeks ago did you pick up your ARVs from the healthcare facility?  If RF, enter 9999.  Question relevant when: ${d13_dk} =1 |  |
| \|  \|  \|  \|  \|  \| d13_dk_days (required) \| \| --- \| --- \| --- \| --- \| --- \| --- \| | How many days ago did you pick up your ARVs from the healthcare facility?  If RF, enter 9999.  Question relevant when: ${d13_dk} =2 |  |
| \|  \|  \|  \|  \|  \| d14 (required) \| \| --- \| --- \| --- \| --- \| --- \| --- \| | How frequently are you supposed to go to the healthcare facility to pick up your ARVs? | \|  \| 1 \| Once a month \| \| --- \| --- \| --- \| \|  \| 2 \| Once every 3 months \| \|  \| 3 \| Once every 6 months \| \|  \| 4 \| My ARVs are brought to my home by a home-based carer \| \|  \| 97 \| Other (specify below) \| \|  \| 99 \| REFUSED \| |
| \|  \|  \|  \|  \|  \| d14_other (required) \| \| --- \| --- \| --- \| --- \| --- \| --- \| | Please specify 'Other' frequency:  Question relevant when: ${d14} =97 |  |
| \|  \|  \|  \|  \|  \| d15 (required) \| \| --- \| --- \| --- \| --- \| --- \| --- \| | A home-based carer is someone from the community who regularly visits households. Home-based carers provide information on how to stay healthy and help care for ill people at their home.  Imagine there are two options to get your ARVs. The first option is to continue having to attend the healthcare facility on a regular basis to pick up ARVs. The second option is for a home-based carers to bring the ARVs to your home free of charge. If you had the choice, which option would you prefer? | \|  \| 1 \| Continue picking up ARVs from a healthcare facility \| \| --- \| --- \| --- \| \|  \| 2 \| Receiving ARVs at home from a home-based carer \| \|  \| 98 \| Don't know \| \|  \| 99 \| REFUSED \| |
| \|  \|  \|  \|  \| note_e \| \| --- \| --- \| --- \| --- \| --- \| | PART 5: COVERAGE OF, AND SATISFACTION WITH HBC SERVICES  I would now like to ask you a few questions about home-based carers. Please bear in mind that all your answers will be treated as highly confidential. No one outside the immediate study team will be told about any answers you gave. |  |
| \|  \|  \|  \|  \| e1 (required) \| \| --- \| --- \| --- \| --- \| --- \| | A home-based carer is someone from the community who regularly visits households. Home-based carers provide information on how to stay healthy and help care for ill people at their home.  Have you ever been visited by a home-based carer? | \|  \| 1 \| Yes \| \| --- \| --- \| --- \| \|  \| 2 \| No \| \|  \| 99 \| REFUSED \| |
| \|  \|  \|  \|  \| e2 (required) \| \| --- \| --- \| --- \| --- \| --- \| | Has your household ever been visited by a home-based carer?  Question relevant when: ${e1} =2 or ${e1} =99 | \|  \| 1 \| Yes \| \| --- \| --- \| --- \| \|  \| 2 \| No \| \|  \| 98 \| Don't know \| \|  \| 99 \| REFUSED \| |
| \|  \|  \|  \|  \| over18_group > hivyes > a5-13_group > hiv_positive > e3_group  Group relevant when: ${e2} =2 \| \| --- \| --- \| --- \| --- \| --- \| | | |
| \|  \|  \|  \|  \|  \| e3 (required) \| \| --- \| --- \| --- \| --- \| --- \| --- \| | In your opinion, why has this household never been visited by a home-based carer?  Read out each option and select all that apply.  Response constrained to: if(selected(., '99'), count-selected(.)=1, count-selected(.)>=1) and if(selected(., '98'), count-selected(.)=1, count-selected(.)>=1) | \|  \| 1 \| The home-based carer has got too many households to take care of \| \| --- \| --- \| --- \| \|  \| 2 \| We have asked the home-based carer not to visit this household \| \|  \| 3 \| No one in our household has been ill \| \|  \| 4 \| The home-based carer mostly visits wealthy households \| \|  \| 5 \| This community does not have a home-based carer \| \|  \| 6 \| We have personal differences with the home-based carer \| \|  \| 7 \| The home-based carer is too old or sick to do his/her job \| \|  \| 8 \| The home-based carer died \| \|  \| 9 \| The home-based carer only visits his/her friends \| \|  \| 10 \| The home-based carer only visits his/her neighbors \| \|  \| 11 \| The home-based carer is lazy \| \|  \| 98 \| I don't know \| \|  \| 97 \| Other (specify below) \| \|  \| 99 \| REFUSED \| |
| \|  \|  \|  \|  \| e3_other (required) \| \| --- \| --- \| --- \| --- \| --- \| | Specify if other reason:  Question relevant when: selected( ${e3} , '97') |  |
| \|  \|  \|  \|  \| over18_group > hivyes > a5-13_group > hiv_positive > skip_e17  Group relevant when: ${e1} =1 or ${e2} =1 \| \| --- \| --- \| --- \| --- \| --- \| | | |
| \|  \|  \|  \|  \|  \| e4 (required) \| \| --- \| --- \| --- \| --- \| --- \| --- \| | When was the last time that a home-based carer came to visit your household?  If respondent DK, enter February 1980. If respondent RF, enter March 1980.  Response constrained to: . <= today() |  |
| \|  \|  \|  \|  \|  \| e5 (required) \| \| --- \| --- \| --- \| --- \| --- \| --- \| | Were you present at the time of the last visit? | \|  \| 1 \| Yes \| \| --- \| --- \| --- \| \|  \| 2 \| No \| \|  \| 99 \| REFUSED \| |
| \|  \|  \|  \|  \|  \| e6 (required) \| \| --- \| --- \| --- \| --- \| --- \| --- \| | When was the last time you were present during a visit by a home-based carer?  If respondent DK, enter February 1980. If respondent RF, enter March 1980.  Question relevant when: ${e5} !=1  Response constrained to: . <= today() |  |
| \|  \|  \|  \|  \|  \| e7 (required) \| \| --- \| --- \| --- \| --- \| --- \| --- \| | During the last six months, how often did a home-based carer visit your household?  If respondent DK, enter "8888." If respondent RF, enter "9999."  Response constrained to: .>-1 |  |
| \|  \|  \|  \|  \|  \| over18_group > hivyes > a5-13_group > hiv_positive > skip_e17 > e8_group \| \| --- \| --- \| --- \| --- \| --- \| --- \| | | |
| \|  \|  \|  \|  \|  \|  \| e8 (required) \| \| --- \| --- \| --- \| --- \| --- \| --- \| --- \| | In your opinion, why does the home-based carer not visit your household more frequently?  Read out each option and select all that apply.  Question relevant when: ${e7} <6  Response constrained to: if(selected(., '99'), count-selected(.)=1, count-selected(.)>=1) and if(selected(., '98'), count-selected(.)=1, count-selected(.)>=1) | \|  \| 1 \| The home-based carer has got too many households to take care of \| \| --- \| --- \| --- \| \|  \| 2 \| We have asked the home-based carer not to visit this household \| \|  \| 3 \| No one in our household has been ill \| \|  \| 4 \| The home-based carer mostly visits wealthy households \| \|  \| 5 \| This community does not have a home-based carer \| \|  \| 6 \| We have personal differences with the home-based carer \| \|  \| 7 \| The home-based carer is too old or sick to do his/her job \| \|  \| 8 \| The home-based carer died \| \|  \| 9 \| The home-based carer only visits his/her friends \| \|  \| 10 \| The home-based carer only visits his/her neighbors \| \|  \| 11 \| The home-based carer is lazy \| \|  \| 98 \| I don't know \| \|  \| 97 \| Other (specify below) \| \|  \| 99 \| REFUSED \| |
| \|  \|  \|  \|  \|  \| e8_other (required) \| \| --- \| --- \| --- \| --- \| --- \| --- \| | Specify if other reason:  Question relevant when: selected( ${e8} , '97') |  |
| \|  \|  \|  \|  \|  \| e9 (required) \| \| --- \| --- \| --- \| --- \| --- \| --- \| | During the last six months, on average, how much time did the home-based carer spend on one visit to your household?  Please enter the number of minutes. If respondent DK, enter "8888." If respondent RF, enter "9999."  Response constrained to: .>-1 |  |
| \|  \|  \|  \|  \|  \| e10 (required) \| \| --- \| --- \| --- \| --- \| --- \| --- \| | In the last six months, have you always been visited by the same home-based carer? | \|  \| 1 \| Yes \| \| --- \| --- \| --- \| \|  \| 2 \| No \| \|  \| 99 \| REFUSED \| |
| \|  \|  \|  \|  \|  \| e11 (required) \| \| --- \| --- \| --- \| --- \| --- \| --- \| | How many different home-based carers have come to visit you in the last six months?  Please enter the number. If respondent DK, enter "8888." If respondent RF, enter "9999."  Question relevant when: ${e10} !=1  Response constrained to: .>-1 |  |
| \|  \|  \|  \|  \|  \| over18_group > hivyes > a5-13_group > hiv_positive > skip_e17 > e12_group \| \| --- \| --- \| --- \| --- \| --- \| --- \| | | |
| \|  \|  \|  \|  \|  \|  \| e12_label \| \| --- \| --- \| --- \| --- \| --- \| --- \| --- \| | Which services has this household received from a home-based carer in the last six months?  Read out each option and select ‘Yes’ ‘No’ 'Don't know' or 'REFUSED' for each. | \|  \| 1 \| Yes \| \| --- \| --- \| --- \| \|  \| 2 \| No \| \|  \| 98 \| Don't know \| \|  \| 99 \| REFUSED \| |
| \|  \|  \|  \|  \|  \|  \| e12_1 (required) \| \| --- \| --- \| --- \| --- \| --- \| --- \| --- \| | Advice on how to stay healthy | \|  \| 1 \| Yes \| \| --- \| --- \| --- \| \|  \| 2 \| No \| \|  \| 98 \| Don't know \| \|  \| 99 \| REFUSED \| |
| \|  \|  \|  \|  \|  \|  \| e12_2 (required) \| \| --- \| --- \| --- \| --- \| --- \| --- \| --- \| | Information on immunizations for children | \|  \| 1 \| Yes \| \| --- \| --- \| --- \| \|  \| 2 \| No \| \|  \| 98 \| Don't know \| \|  \| 99 \| REFUSED \| |
| \|  \|  \|  \|  \|  \|  \| e12_3 (required) \| \| --- \| --- \| --- \| --- \| --- \| --- \| --- \| | Information and advice on feeding of babies | \|  \| 1 \| Yes \| \| --- \| --- \| --- \| \|  \| 2 \| No \| \|  \| 98 \| Don't know \| \|  \| 99 \| REFUSED \| |
| \|  \|  \|  \|  \|  \|  \| e12_4 (required) \| \| --- \| --- \| --- \| --- \| --- \| --- \| --- \| | Measuring a child’s height and weight | \|  \| 1 \| Yes \| \| --- \| --- \| --- \| \|  \| 2 \| No \| \|  \| 98 \| Don't know \| \|  \| 99 \| REFUSED \| |
| \|  \|  \|  \|  \|  \|  \| e12_5 (required) \| \| --- \| --- \| --- \| --- \| --- \| --- \| --- \| | Information on pregnancy and childbirth | \|  \| 1 \| Yes \| \| --- \| --- \| --- \| \|  \| 2 \| No \| \|  \| 98 \| Don't know \| \|  \| 99 \| REFUSED \| |
| \|  \|  \|  \|  \|  \|  \| e12_6 (required) \| \| --- \| --- \| --- \| --- \| --- \| --- \| --- \| | Checking if a pregnancy is going well | \|  \| 1 \| Yes \| \| --- \| --- \| --- \| \|  \| 2 \| No \| \|  \| 98 \| Don't know \| \|  \| 99 \| REFUSED \| |
| \|  \|  \|  \|  \|  \|  \| e12_7 (required) \| \| --- \| --- \| --- \| --- \| --- \| --- \| --- \| | Delivery of a baby at home | \|  \| 1 \| Yes \| \| --- \| --- \| --- \| \|  \| 2 \| No \| \|  \| 98 \| Don't know \| \|  \| 99 \| REFUSED \| |
| \|  \|  \|  \|  \|  \|  \| e12_8 (required) \| \| --- \| --- \| --- \| --- \| --- \| --- \| --- \| | Checking if a newborn is healthy | \|  \| 1 \| Yes \| \| --- \| --- \| --- \| \|  \| 2 \| No \| \|  \| 98 \| Don't know \| \|  \| 99 \| REFUSED \| |
| \|  \|  \|  \|  \|  \|  \| e12_9 (required) \| \| --- \| --- \| --- \| --- \| --- \| --- \| --- \| | Distributing food | \|  \| 1 \| Yes \| \| --- \| --- \| --- \| \|  \| 2 \| No \| \|  \| 98 \| Don't know \| \|  \| 99 \| REFUSED \| |
| \|  \|  \|  \|  \|  \|  \| e12_10 (required) \| \| --- \| --- \| --- \| --- \| --- \| --- \| --- \| | Advice or help with sanitation, such as toilets | \|  \| 1 \| Yes \| \| --- \| --- \| --- \| \|  \| 2 \| No \| \|  \| 98 \| Don't know \| \|  \| 99 \| REFUSED \| |
| \|  \|  \|  \|  \|  \|  \| e12_11 (required) \| \| --- \| --- \| --- \| --- \| --- \| --- \| --- \| | Referral to a healthcare facility when someone was ill | \|  \| 1 \| Yes \| \| --- \| --- \| --- \| \|  \| 2 \| No \| \|  \| 98 \| Don't know \| \|  \| 99 \| REFUSED \| |
| \|  \|  \|  \|  \|  \|  \| e12_12 (required) \| \| --- \| --- \| --- \| --- \| --- \| --- \| --- \| | Care at home when someone was ill | \|  \| 1 \| Yes \| \| --- \| --- \| --- \| \|  \| 2 \| No \| \|  \| 98 \| Don't know \| \|  \| 99 \| REFUSED \| |
| \|  \|  \|  \|  \|  \| over18_group > hivyes > a5-13_group > hiv_positive > skip_e17 > e12_group2 \| \| --- \| --- \| --- \| --- \| --- \| --- \| | | |
| \|  \|  \|  \|  \|  \|  \| e12_label2 \| \| --- \| --- \| --- \| --- \| --- \| --- \| --- \| | Which services has this household received from a home-based carer in the last six months? | \|  \| 1 \| Yes \| \| --- \| --- \| --- \| \|  \| 2 \| No \| \|  \| 98 \| Don't know \| \|  \| 99 \| REFUSED \| |
| \|  \|  \|  \|  \|  \|  \| e12_13 (required) \| \| --- \| --- \| --- \| --- \| --- \| --- \| --- \| | Care at home when someone was dying | \|  \| 1 \| Yes \| \| --- \| --- \| --- \| \|  \| 2 \| No \| \|  \| 98 \| Don't know \| \|  \| 99 \| REFUSED \| |
| \|  \|  \|  \|  \|  \|  \| e12_14 (required) \| \| --- \| --- \| --- \| --- \| --- \| --- \| --- \| | First aid in an emergency | \|  \| 1 \| Yes \| \| --- \| --- \| --- \| \|  \| 2 \| No \| \|  \| 98 \| Don't know \| \|  \| 99 \| REFUSED \| |
| \|  \|  \|  \|  \|  \|  \| e12_15 (required) \| \| --- \| --- \| --- \| --- \| --- \| --- \| --- \| | Observing someone taking their medication | \|  \| 1 \| Yes \| \| --- \| --- \| --- \| \|  \| 2 \| No \| \|  \| 98 \| Don't know \| \|  \| 99 \| REFUSED \| |
| \|  \|  \|  \|  \|  \|  \| e12_16 (required) \| \| --- \| --- \| --- \| --- \| --- \| --- \| --- \| | Information on family planning | \|  \| 1 \| Yes \| \| --- \| --- \| --- \| \|  \| 2 \| No \| \|  \| 98 \| Don't know \| \|  \| 99 \| REFUSED \| |
| \|  \|  \|  \|  \|  \|  \| e12_17 (required) \| \| --- \| --- \| --- \| --- \| --- \| --- \| --- \| | Carrying out a pregnancy test | \|  \| 1 \| Yes \| \| --- \| --- \| --- \| \|  \| 2 \| No \| \|  \| 98 \| Don't know \| \|  \| 99 \| REFUSED \| |
| \|  \|  \|  \|  \|  \|  \| e12_18 (required) \| \| --- \| --- \| --- \| --- \| --- \| --- \| --- \| | Providing contraceptives | \|  \| 1 \| Yes \| \| --- \| --- \| --- \| \|  \| 2 \| No \| \|  \| 98 \| Don't know \| \|  \| 99 \| REFUSED \| |
| \|  \|  \|  \|  \|  \|  \| e12_19 (required) \| \| --- \| --- \| --- \| --- \| --- \| --- \| --- \| | Providing condoms | \|  \| 1 \| Yes \| \| --- \| --- \| --- \| \|  \| 2 \| No \| \|  \| 98 \| Don't know \| \|  \| 99 \| REFUSED \| |
| \|  \|  \|  \|  \|  \|  \| e12_20 (required) \| \| --- \| --- \| --- \| --- \| --- \| --- \| --- \| | Providing HIV medicines | \|  \| 1 \| Yes \| \| --- \| --- \| --- \| \|  \| 2 \| No \| \|  \| 98 \| Don't know \| \|  \| 99 \| REFUSED \| |
| \|  \|  \|  \|  \|  \|  \| e12_21 (required) \| \| --- \| --- \| --- \| --- \| --- \| --- \| --- \| | Providing medication for tuberculosis | \|  \| 1 \| Yes \| \| --- \| --- \| --- \| \|  \| 2 \| No \| \|  \| 98 \| Don't know \| \|  \| 99 \| REFUSED \| |
| \|  \|  \|  \|  \|  \|  \| e12_22 (required) \| \| --- \| --- \| --- \| --- \| --- \| --- \| --- \| | Providing other medication | \|  \| 1 \| Yes \| \| --- \| --- \| --- \| \|  \| 2 \| No \| \|  \| 98 \| Don't know \| \|  \| 99 \| REFUSED \| |
| \|  \|  \|  \|  \|  \|  \| e12_23 (required) \| \| --- \| --- \| --- \| --- \| --- \| --- \| --- \| | HIV-testing | \|  \| 1 \| Yes \| \| --- \| --- \| --- \| \|  \| 2 \| No \| \|  \| 98 \| Don't know \| \|  \| 99 \| REFUSED \| |
| \|  \|  \|  \|  \|  \|  \| e12_24 (required) \| \| --- \| --- \| --- \| --- \| --- \| --- \| --- \| | Screening for tuberculosis | \|  \| 1 \| Yes \| \| --- \| --- \| --- \| \|  \| 2 \| No \| \|  \| 98 \| Don't know \| \|  \| 99 \| REFUSED \| |
| \|  \|  \|  \|  \|  \|  \| e12_25 (required) \| \| --- \| --- \| --- \| --- \| --- \| --- \| --- \| | Other (SPECIFY) | \|  \| 1 \| Yes \| \| --- \| --- \| --- \| \|  \| 2 \| No \| \|  \| 98 \| Don't know \| \|  \| 99 \| REFUSED \| |
| \|  \|  \|  \|  \|  \|  \| e12_25other \| \| --- \| --- \| --- \| --- \| --- \| --- \| --- \| | Please specify 'Other' service:  Question relevant when: selected( ${e12_25} , 1) |  |
| \|  \|  \|  \|  \|  \| e13 (required) \| \| --- \| --- \| --- \| --- \| --- \| --- \| | Overall, how satisfied or dissatisfied are you with the services provided by the home-based carers in your community?  Ask the respondent to refer to scale 1. Please tick a number on the scale. | \|  \| 0 \| 0 (Very dissatisfied) \| \| --- \| --- \| --- \| \|  \| 1 \| 1 \| \|  \| 2 \| 2 \| \|  \| 3 \| 3 \| \|  \| 4 \| 4 \| \|  \| 5 \| 5 \| \|  \| 6 \| 6 \| \|  \| 7 \| 7 \| \|  \| 8 \| 8 \| \|  \| 9 \| 9 \| \|  \| 10 \| 10 (Very satisfied) \| \|  \| 99 \| REFUSED \| |
| \|  \|  \|  \|  \|  \| e14 (required) \| \| --- \| --- \| --- \| --- \| --- \| --- \| | How satisfied or dissatisfied are you with the accessibility of the home-based carers in your community? With accessibility we mean your ability to see a home-based carer when you are ill or looking for advice.  Ask the respondent to refer to scale 1. Please tick a number on the scale. | \|  \| 0 \| 0 (Very dissatisfied) \| \| --- \| --- \| --- \| \|  \| 1 \| 1 \| \|  \| 2 \| 2 \| \|  \| 3 \| 3 \| \|  \| 4 \| 4 \| \|  \| 5 \| 5 \| \|  \| 6 \| 6 \| \|  \| 7 \| 7 \| \|  \| 8 \| 8 \| \|  \| 9 \| 9 \| \|  \| 10 \| 10 (Very satisfied) \| \|  \| 99 \| REFUSED \| |
| \|  \|  \|  \|  \|  \| e15 (required) \| \| --- \| --- \| --- \| --- \| --- \| --- \| | How satisfied or dissatisfied are you with the quality of the advice and care given by the home-based carers in your community?  Ask the respondent to refer to scale 1. Please tick a number on the scale. | \|  \| 0 \| 0 (Very dissatisfied) \| \| --- \| --- \| --- \| \|  \| 1 \| 1 \| \|  \| 2 \| 2 \| \|  \| 3 \| 3 \| \|  \| 4 \| 4 \| \|  \| 5 \| 5 \| \|  \| 6 \| 6 \| \|  \| 7 \| 7 \| \|  \| 8 \| 8 \| \|  \| 9 \| 9 \| \|  \| 10 \| 10 (Very satisfied) \| \|  \| 99 \| REFUSED \| |
| \|  \|  \|  \|  \|  \| e16 (required) \| \| --- \| --- \| --- \| --- \| --- \| --- \| | How satisfied or dissatisfied are you with being treated respectfully by home-based carers?  Ask the respondent to refer to scale 1. Please tick a number on the scale. | \|  \| 0 \| 0 (Very dissatisfied) \| \| --- \| --- \| --- \| \|  \| 1 \| 1 \| \|  \| 2 \| 2 \| \|  \| 3 \| 3 \| \|  \| 4 \| 4 \| \|  \| 5 \| 5 \| \|  \| 6 \| 6 \| \|  \| 7 \| 7 \| \|  \| 8 \| 8 \| \|  \| 9 \| 9 \| \|  \| 10 \| 10 (Very satisfied) \| \|  \| 99 \| REFUSED \| |
| \|  \|  \|  \|  \| e17 (required) \| \| --- \| --- \| --- \| --- \| --- \| | Do you trust the home-based carers in your area to keep information about your health confidential? With confidential, we mean that the home-based carer does not tell other people about your health without your permission. | \|  \| 1 \| Yes, I trust the home-based carers with my medical information \| \| --- \| --- \| --- \| \|  \| 2 \| No, I don’t trust the home-based carers with my medical information \| \|  \| 3 \| I can’t answer this question because I don’t know who the home-based carers are \| \|  \| 99 \| REFUSED \| |
| \|  \|  \|  \|  \| e18 (required) \| \| --- \| --- \| --- \| --- \| --- \| | Would you recommend the home-based carer program to other communities? | \|  \| 1 \| Yes \| \| --- \| --- \| --- \| \|  \| 2 \| No \| \|  \| 99 \| REFUSED \| |
| \|  \|  \|  \|  \| e19 (required) \| \| --- \| --- \| --- \| --- \| --- \| | Apart from home-based carers, have any other people come to your house to provide information about health, to offer testing for an illness, or to ask you to come to a healthcare facility? | \|  \| 1 \| Yes \| \| --- \| --- \| --- \| \|  \| 2 \| No \| \|  \| 99 \| REFUSED \| |
| \|  \|  \|  \|  \| e20 (required) \| \| --- \| --- \| --- \| --- \| --- \| | Let us refer to this person or these people as “community health workers” in the next few questions. Other than home-based carers, how many community health workers have come to visit you at your home in the last one year?  Please enter the number. If respondent DK, enter "8888." If respondent RF, enter "9999."  Question relevant when: ${e19} =1  Response constrained to: .>-1 |  |
| \|  \|  \|  \|  \| e21 (required) \| \| --- \| --- \| --- \| --- \| --- \| | During the last one year, how often did these community health workers come to visit your household?  Please enter the number. If respondent DK, enter "8888." If respondent RF, enter "9999."  Question relevant when: ${e19} =1  Response constrained to: .>-1 |  |
| \|  \|  \|  \|  \| e22 (required) \| \| --- \| --- \| --- \| --- \| --- \| | The following questions try to find out if patients are interested in community health worker services. But this does NOT mean that we will offer community health worker services to you.  Please think of a community health worker as someone who visits households on a regular basis to provide advice on health issues or to care for those who are ill. Would you like to receive home visits from a community health worker? | \|  \| 1 \| Yes \| \| --- \| --- \| --- \| \|  \| 2 \| No \| \|  \| 99 \| REFUSED \| |
| \|  \|  \|  \|  \| over18_group > hivyes > a5-13_group > hiv_positive > e22_yes  Group relevant when: ${e22} =1 \| \| --- \| --- \| --- \| --- \| --- \| | | |
| \|  \|  \|  \|  \|  \| e23 (required) \| \| --- \| --- \| --- \| --- \| --- \| --- \| | Would you like to receive these home visits by a community health worker once a week, once a month, once every 3 months, or once a year?  Question relevant when: ${e22} =1 | \|  \| 1 \| Once a week \| \| --- \| --- \| --- \| \|  \| 2 \| Once a month \| \|  \| 3 \| Once every 3 months \| \|  \| 4 \| Once every 6 months \| \|  \| 99 \| REFUSED \| |
| \|  \|  \|  \|  \|  \| over18_group > hivyes > a5-13_group > hiv_positive > e22_yes > e24  Group relevant when: ${e22} =1 \| \| --- \| --- \| --- \| --- \| --- \| --- \| | | |
| \|  \|  \|  \|  \|  \|  \| e24 \| \| --- \| --- \| --- \| --- \| --- \| --- \| --- \| | Which of the following services would you like to receive from a community health worker who visits your household?  Read out each option and select ‘Yes’ ‘No’ 'Don't know' or 'REFUSED' for each. | \|  \| 1 \| Yes \| \| --- \| --- \| --- \| \|  \| 2 \| No \| \|  \| 98 \| Don't know \| \|  \| 99 \| REFUSED \| |
| \|  \|  \|  \|  \|  \|  \| e24_1 (required) \| \| --- \| --- \| --- \| --- \| --- \| --- \| --- \| | Advice on how to stay healthy | \|  \| 1 \| Yes \| \| --- \| --- \| --- \| \|  \| 2 \| No \| \|  \| 98 \| Don't know \| \|  \| 99 \| REFUSED \| |
| \|  \|  \|  \|  \|  \|  \| e24_2 (required) \| \| --- \| --- \| --- \| --- \| --- \| --- \| --- \| | Information on immunizations for children | \|  \| 1 \| Yes \| \| --- \| --- \| --- \| \|  \| 2 \| No \| \|  \| 98 \| Don't know \| \|  \| 99 \| REFUSED \| |
| \|  \|  \|  \|  \|  \|  \| e24_3 (required) \| \| --- \| --- \| --- \| --- \| --- \| --- \| --- \| | Information and advice on feeding of babies | \|  \| 1 \| Yes \| \| --- \| --- \| --- \| \|  \| 2 \| No \| \|  \| 98 \| Don't know \| \|  \| 99 \| REFUSED \| |
| \|  \|  \|  \|  \|  \|  \| e24_4 (required) \| \| --- \| --- \| --- \| --- \| --- \| --- \| --- \| | Measuring a child’s height and weight | \|  \| 1 \| Yes \| \| --- \| --- \| --- \| \|  \| 2 \| No \| \|  \| 98 \| Don't know \| \|  \| 99 \| REFUSED \| |
| \|  \|  \|  \|  \|  \|  \| e24_5 (required) \| \| --- \| --- \| --- \| --- \| --- \| --- \| --- \| | Information on pregnancy and childbirth | \|  \| 1 \| Yes \| \| --- \| --- \| --- \| \|  \| 2 \| No \| \|  \| 98 \| Don't know \| \|  \| 99 \| REFUSED \| |
| \|  \|  \|  \|  \|  \|  \| e24_6 (required) \| \| --- \| --- \| --- \| --- \| --- \| --- \| --- \| | Checking if a pregnancy is going well | \|  \| 1 \| Yes \| \| --- \| --- \| --- \| \|  \| 2 \| No \| \|  \| 98 \| Don't know \| \|  \| 99 \| REFUSED \| |
| \|  \|  \|  \|  \|  \|  \| e24_7 (required) \| \| --- \| --- \| --- \| --- \| --- \| --- \| --- \| | Delivery of a baby at home | \|  \| 1 \| Yes \| \| --- \| --- \| --- \| \|  \| 2 \| No \| \|  \| 98 \| Don't know \| \|  \| 99 \| REFUSED \| |
| \|  \|  \|  \|  \|  \|  \| e24_8 (required) \| \| --- \| --- \| --- \| --- \| --- \| --- \| --- \| | Checking if a newborn is healthy | \|  \| 1 \| Yes \| \| --- \| --- \| --- \| \|  \| 2 \| No \| \|  \| 98 \| Don't know \| \|  \| 99 \| REFUSED \| |
| \|  \|  \|  \|  \|  \|  \| e24_9 (required) \| \| --- \| --- \| --- \| --- \| --- \| --- \| --- \| | Distributing food | \|  \| 1 \| Yes \| \| --- \| --- \| --- \| \|  \| 2 \| No \| \|  \| 98 \| Don't know \| \|  \| 99 \| REFUSED \| |
| \|  \|  \|  \|  \|  \|  \| e24_10 (required) \| \| --- \| --- \| --- \| --- \| --- \| --- \| --- \| | Advice or help with sanitation, such as toilets | \|  \| 1 \| Yes \| \| --- \| --- \| --- \| \|  \| 2 \| No \| \|  \| 98 \| Don't know \| \|  \| 99 \| REFUSED \| |
| \|  \|  \|  \|  \|  \|  \| e24_11 (required) \| \| --- \| --- \| --- \| --- \| --- \| --- \| --- \| | Referral to a healthcare facility when someone is ill | \|  \| 1 \| Yes \| \| --- \| --- \| --- \| \|  \| 2 \| No \| \|  \| 98 \| Don't know \| \|  \| 99 \| REFUSED \| |
| \|  \|  \|  \|  \|  \|  \| e24_12 (required) \| \| --- \| --- \| --- \| --- \| --- \| --- \| --- \| | An injection to treat illnesses | \|  \| 1 \| Yes \| \| --- \| --- \| --- \| \|  \| 2 \| No \| \|  \| 98 \| Don't know \| \|  \| 99 \| REFUSED \| |
| \|  \|  \|  \|  \|  \|  \| e24_13 (required) \| \| --- \| --- \| --- \| --- \| --- \| --- \| --- \| | Care at home when someone is ill | \|  \| 1 \| Yes \| \| --- \| --- \| --- \| \|  \| 2 \| No \| \|  \| 98 \| Don't know \| \|  \| 99 \| REFUSED \| |
| \|  \|  \|  \|  \|  \| over18_group > hivyes > a5-13_group > hiv_positive > e22_yes > e24_group2  Group relevant when: ${e22} =1 \| \| --- \| --- \| --- \| --- \| --- \| --- \| | | |
| \|  \|  \|  \|  \|  \|  \| e24_b \| \| --- \| --- \| --- \| --- \| --- \| --- \| --- \| | (Continued) Which of the following services would you like to receive from a community health worker who visits your household?  Read out each option and select ‘Yes’ ‘No’ 'Don't know' or 'REFUSED' for each. | \|  \| 1 \| Yes \| \| --- \| --- \| --- \| \|  \| 2 \| No \| \|  \| 98 \| Don't know \| \|  \| 99 \| REFUSED \| |
| \|  \|  \|  \|  \|  \|  \| e24_14 (required) \| \| --- \| --- \| --- \| --- \| --- \| --- \| --- \| | Care at home when someone is dying | \|  \| 1 \| Yes \| \| --- \| --- \| --- \| \|  \| 2 \| No \| \|  \| 98 \| Don't know \| \|  \| 99 \| REFUSED \| |
| \|  \|  \|  \|  \|  \|  \| e24_15 (required) \| \| --- \| --- \| --- \| --- \| --- \| --- \| --- \| | First aid in an emergency | \|  \| 1 \| Yes \| \| --- \| --- \| --- \| \|  \| 2 \| No \| \|  \| 98 \| Don't know \| \|  \| 99 \| REFUSED \| |
| \|  \|  \|  \|  \|  \|  \| e24_16 (required) \| \| --- \| --- \| --- \| --- \| --- \| --- \| --- \| | Observing someone taking their medication | \|  \| 1 \| Yes \| \| --- \| --- \| --- \| \|  \| 2 \| No \| \|  \| 98 \| Don't know \| \|  \| 99 \| REFUSED \| |
| \|  \|  \|  \|  \|  \|  \| e24_17 (required) \| \| --- \| --- \| --- \| --- \| --- \| --- \| --- \| | Information on family planning | \|  \| 1 \| Yes \| \| --- \| --- \| --- \| \|  \| 2 \| No \| \|  \| 98 \| Don't know \| \|  \| 99 \| REFUSED \| |
| \|  \|  \|  \|  \|  \|  \| e24_18 (required) \| \| --- \| --- \| --- \| --- \| --- \| --- \| --- \| | Providing contraceptives | \|  \| 1 \| Yes \| \| --- \| --- \| --- \| \|  \| 2 \| No \| \|  \| 98 \| Don't know \| \|  \| 99 \| REFUSED \| |
| \|  \|  \|  \|  \|  \|  \| e24_19 (required) \| \| --- \| --- \| --- \| --- \| --- \| --- \| --- \| | Injections for contraception | \|  \| 1 \| Yes \| \| --- \| --- \| --- \| \|  \| 2 \| No \| \|  \| 98 \| Don't know \| \|  \| 99 \| REFUSED \| |
| \|  \|  \|  \|  \|  \|  \| e24_20 (required) \| \| --- \| --- \| --- \| --- \| --- \| --- \| --- \| | Providing condoms | \|  \| 1 \| Yes \| \| --- \| --- \| --- \| \|  \| 2 \| No \| \|  \| 98 \| Don't know \| \|  \| 99 \| REFUSED \| |
| \|  \|  \|  \|  \|  \|  \| e24_21 (required) \| \| --- \| --- \| --- \| --- \| --- \| --- \| --- \| | Providing a pregnancy test | \|  \| 1 \| Yes \| \| --- \| --- \| --- \| \|  \| 2 \| No \| \|  \| 98 \| Don't know \| \|  \| 99 \| REFUSED \| |
| \|  \|  \|  \|  \|  \|  \| e24_22 (required) \| \| --- \| --- \| --- \| --- \| --- \| --- \| --- \| | Providing medication for HIV | \|  \| 1 \| Yes \| \| --- \| --- \| --- \| \|  \| 2 \| No \| \|  \| 98 \| Don't know \| \|  \| 99 \| REFUSED \| |
| \|  \|  \|  \|  \|  \|  \| e24_23 (required) \| \| --- \| --- \| --- \| --- \| --- \| --- \| --- \| | Providing medication for tuberculosis | \|  \| 1 \| Yes \| \| --- \| --- \| --- \| \|  \| 2 \| No \| \|  \| 98 \| Don't know \| \|  \| 99 \| REFUSED \| |
| \|  \|  \|  \|  \|  \|  \| e24_24 (required) \| \| --- \| --- \| --- \| --- \| --- \| --- \| --- \| | Providing other medication | \|  \| 1 \| Yes \| \| --- \| --- \| --- \| \|  \| 2 \| No \| \|  \| 98 \| Don't know \| \|  \| 99 \| REFUSED \| |
| \|  \|  \|  \|  \|  \|  \| e24_25 (required) \| \| --- \| --- \| --- \| --- \| --- \| --- \| --- \| | HIV-testing | \|  \| 1 \| Yes \| \| --- \| --- \| --- \| \|  \| 2 \| No \| \|  \| 98 \| Don't know \| \|  \| 99 \| REFUSED \| |
| \|  \|  \|  \|  \|  \|  \| e24_26 (required) \| \| --- \| --- \| --- \| --- \| --- \| --- \| --- \| | Screening for tuberculosis | \|  \| 1 \| Yes \| \| --- \| --- \| --- \| \|  \| 2 \| No \| \|  \| 98 \| Don't know \| \|  \| 99 \| REFUSED \| |
| \|  \|  \|  \|  \|  \|  \| e24_27 (required) \| \| --- \| --- \| --- \| --- \| --- \| --- \| --- \| | Other (SPECIFY ON THE NEXT PAGE) | \|  \| 1 \| Yes \| \| --- \| --- \| --- \| \|  \| 2 \| No \| \|  \| 98 \| Don't know \| \|  \| 99 \| REFUSED \| |
| \|  \|  \|  \|  \|  \| e24_27other (required) \| \| --- \| --- \| --- \| --- \| --- \| --- \| | PLEASE SPECIFY 'Other' SERVICE:  Question relevant when: ${e24_27} =1 |  |
| \|  \|  \|  \|  \| e25 (required) \| \| --- \| --- \| --- \| --- \| --- \| | Would you like to receive home visits from a community health worker when you are ill? | \|  \| 1 \| Yes \| \| --- \| --- \| --- \| \|  \| 2 \| No \| \|  \| 99 \| REFUSED \| |
| \|  \|  \|  \|  \| e26 (required) \| \| --- \| --- \| --- \| --- \| --- \| | Would you like to receive home visits from a community health worker when you are not able to care for yourself? | \|  \| 1 \| Yes \| \| --- \| --- \| --- \| \|  \| 2 \| No \| \|  \| 99 \| REFUSED \| |
| \|  \|  \|  \|  \| e27 (required) \| \| --- \| --- \| --- \| --- \| --- \| | Would you like to receive home visits from a community health worker when you are dying? | \|  \| 1 \| Yes \| \| --- \| --- \| --- \| \|  \| 2 \| No \| \|  \| 99 \| REFUSED \| |
| \|  \|  \|  \|  \| time_end \| \| --- \| --- \| --- \| --- \| --- \| | Time at end of interview |  |
| \|  \|  \|  \|  \| respondent_comments \| \| --- \| --- \| --- \| --- \| --- \| | Thank you very much for your effort and time!  Do you have any comments or feedback for us? |  |
| \|  \|  \|  \|  \| ready_eligibility \| \| --- \| --- \| --- \| --- \| --- \| | Are you ready to answer patient eligibility questions?  Please return to this page and select 'Yes' when ready. | \|  \| 1 \| Yes \| \| --- \| --- \| --- \| \|  \| 2 \| No \| |
| \|  \|  \|  \|  \| over18_group > hivyes > a5-13_group > hiv_positive > eligibility  Group relevant when: ${ready_eligibility} =1 \| \| --- \| --- \| --- \| --- \| --- \| | | |
| \|  \|  \|  \|  \|  \| aa1 (required) \| \| --- \| --- \| --- \| --- \| --- \| --- \| | Eligibility  Patient CTC2: [ctc2]  Has this participant been receiving ART for at least 6 months? | \|  \| 1 \| Yes \| \| --- \| --- \| --- \| \|  \| 2 \| No \| |
| \|  \|  \|  \|  \|  \| aa2 (required) \| \| --- \| --- \| --- \| --- \| --- \| --- \| | Does this patient have a viral load taken in the last 12 months?  Question relevant when: ${aa1} =1 | \|  \| 1 \| Yes \| \| --- \| --- \| --- \| \|  \| 2 \| No \| |
| \|  \|  \|  \|  \|  \| aa3 (required) \| \| --- \| --- \| --- \| --- \| --- \| --- \| | Is the viral load suppressed?  Question relevant when: ${aa2} =1 | \|  \| 1 \| Yes \| \| --- \| --- \| --- \| \|  \| 2 \| No \| |
| \|  \|  \|  \|  \|  \| aa4 (required) \| \| --- \| --- \| --- \| --- \| --- \| --- \| | Had the participant been receiving ART for at least 6 months when the most current viral load was measured?  Question relevant when: ${aa3} =1 | \|  \| 1 \| Yes \| \| --- \| --- \| --- \| \|  \| 2 \| No \| |
| \|  \|  \|  \|  \|  \| aa5 (required) \| \| --- \| --- \| --- \| --- \| --- \| --- \| | Does this patient have a CD4-count taken in the last 12 months?  Question relevant when: ${aa4} =2 or ${aa2} =2 or ${aa3} =2 | \|  \| 1 \| Yes \| \| --- \| --- \| --- \| \|  \| 2 \| No \| |
| \|  \|  \|  \|  \|  \| aa6 (required) \| \| --- \| --- \| --- \| --- \| --- \| --- \| | Is the most current CD4-count greater than 350?  Question relevant when: ${aa5} =1 | \|  \| 1 \| Yes \| \| --- \| --- \| --- \| \|  \| 2 \| No \| |
| \|  \|  \|  \|  \|  \| aa7 (required) \| \| --- \| --- \| --- \| --- \| --- \| --- \| | Had the participant been receiving ART for at least 6 months when the most current CD4-count was measured?  Question relevant when: ${aa6} =1 | \|  \| 1 \| Yes \| \| --- \| --- \| --- \| \|  \| 2 \| No \| |
| \|  \|  \|  \|  \| over18_group > hivyes > a5-13_group > hiv_positive > labs_group \| \| --- \| --- \| --- \| --- \| --- \| | | |
| \|  \|  \|  \|  \|  \| labs_note \| \| --- \| --- \| --- \| --- \| --- \| --- \| | SAVE AND EXIT THIS SURVEY, THEN RETURN TO THIS PAGE TO ENTER THE CD4 COUNTS AND VIRAL LOAD DATA FOR THIS PATIENT. |  |
| \|  \|  \|  \|  \|  \| labs_note2 \| \| --- \| --- \| --- \| --- \| --- \| --- \| | Please confirm that you have the correct patient:  CTC2 number: [ctc2] |  |
| \|  \|  \|  \|  \|  \| VL_date (required) \| \| --- \| --- \| --- \| --- \| --- \| --- \| | When was the blood sample for the last viral load taken?  If respondent has never had a viral load measurement, enter February 1980.  Response constrained to: . <= today() |  |
| \|  \|  \|  \|  \|  \| VL_suppressed (required) \| \| --- \| --- \| --- \| --- \| --- \| --- \| | Was the patient's viral load suppressed? | \|  \| 1 \| Yes \| \| --- \| --- \| --- \| \|  \| 2 \| No \| |
| \|  \|  \|  \|  \|  \| VL_number (required) \| \| --- \| --- \| --- \| --- \| --- \| --- \| | What was the patient's viral load?  Please answer in copies/mL. Enter 0 if the viral load was suppressed. If no viral load was taken, enter 99999.  Response constrained to: .>-1 |  |
| \|  \|  \|  \|  \|  \| CD4_date (required) \| \| --- \| --- \| --- \| --- \| --- \| --- \| | When was the blood sample for the last CD4 count taken?  If respondent has never had a CD4 count measurement, enter February 1980.  Response constrained to: . <= today() |  |
| \|  \|  \|  \|  \|  \| CD4_number (required) \| \| --- \| --- \| --- \| --- \| --- \| --- \| | What was the patient's CD4-count?  Please answer in cells/mL. If no CD4 count was taken, enter 99999.  Response constrained to: .>-1 |  |
| \|  \|  \|  \|  \|  \| started_ARVs (required) \| \| --- \| --- \| --- \| --- \| --- \| --- \| | Has this patient been started on ARVs at this healthcare facility? | \|  \| 1 \| Yes \| \| --- \| --- \| --- \| \|  \| 2 \| No \| |
| \|  \|  \|  \|  \| date_started_ARVs (required) \| \| --- \| --- \| --- \| --- \| --- \| | Please enter the date on which the patient was started on ARVs.  Question relevant when: ${started_ARVs} =1  Response constrained to: . <= today() |  |
| \|  \|  \|  \|  \| over18_group > hivyes > a5-13_group > hiv_positive > confirm_group \| \| --- \| --- \| --- \| --- \| --- \| | | |
| \|  \|  \|  \|  \|  \| confirm_note \| \| --- \| --- \| --- \| --- \| --- \| --- \| | Before finalizing this survey on the next page, PLEASE CONFIRM: |  |
| \|  \|  \|  \|  \|  \| eligibility_done (required) \| \| --- \| --- \| --- \| --- \| --- \| --- \| | 1. Did you fill out the patient eligiblity questions? | \|  \| 1 \| Yes \| \| --- \| --- \| --- \| |
| \|  \|  \|  \|  \|  \| lab_done (required) \| \| --- \| --- \| --- \| --- \| --- \| --- \| | 2. Did you complete the patient lab information? | \|  \| 1 \| Yes \| \| --- \| --- \| --- \| |
