# Supplementary material for: The impact of community health worker-led home delivery of antiretroviral therapy on virological suppression: a non-inferiority cluster-randomized health systems trial in Dar es Salaam, Tanzania
Source: BMC Health Serv Res. 2017 Feb 22;17:160. doi: 10.1186/s12913-017-2032-7 (PMC5322683; doi:10.1186/s12913-017-2032-7)
Supplement: Additional file 3: — Healthcare Provider Questionnaire. (DOCX 142 kb) [file 12913_2017_2032_MOESM3_ESM.docx]

#### Healthcare Provider Questionnaire

| Field | Question | Answer |
| --- | --- | --- |
| intronote | Welcome to the Healthcare Provider Questionnaire. Please swipe forward to continue. |  |
| 0.1: INTERVIEWER INFORMATION | | |
| \|  \| interviewer_id (required) \| \| --- \| --- \| | Select interviewer name: | \|  \| 1 \| Grace Joseph Matemu \| \| --- \| --- \| --- \| \|  \| 2 \| Geofrey Isdory \| \|  \| 3 \| Glory William \| \|  \| 4 \| Tunkine Sanga \| \|  \| 5 \| Willy Ulkaye \| \|  \| 6 \| Lilian Lwanda \| \|  \| 7 \| Sakina Hamisi \| \|  \| 8 \| Happiness Madadi \| \|  \| 9 \| Mgalama Jaqueline \| \|  \| 10 \| Jeila Maulid \| \|  \| 11 \| Aidath Murusuri \| \|  \| 12 \| Judith Mmari \| \|  \| 13 \| Joan Reno Mori \| \|  \| 14 \| Paul Msale \| \|  \| 15 \| Charles Kafula \| \|  \| 16 \| Josephine Uiso \| \|  \| 17 \| Irene Machume \| \|  \| 18 \| Flora Musa \| \|  \| 97 \| OTHER \| |
| \|  \| 0.1: INTERVIEWER INFORMATION > -  Group relevant when: ${interviewer_id} =97 \| \| --- \| --- \| | | |
| \|  \|  \| other_interviewer_note \| \| --- \| --- \| --- \| | You selected 'OTHER' interviewer. Please enter your first and last name. |  |
| \|  \|  \| other_interviewer_first (required) \| \| --- \| --- \| --- \| | First name of interviewer: |  |
| \|  \|  \| other_interviewer_last (required) \| \| --- \| --- \| --- \| | Last name of interviewer: |  |
| \|  \| facility (required) \| \| --- \| --- \| | Select facility: | \|  \| 1 \| Temeke Hospital \| \| --- \| --- \| --- \| \|  \| 2 \| Kigamboni \| \|  \| 3 \| Mbagala Tangi tatu \| \|  \| 4 \| Tambukareli \| \|  \| 5 \| Maji Matitu \| \|  \| 6 \| Yombo Makangarawe \| \|  \| 7 \| Mbagala Round Table \| \|  \| 8 \| Buza \| \|  \| 9 \| Kingugi \| \|  \| 10 \| Kimbiji \| \|  \| 11 \| Toa Ngoma \| \|  \| 12 \| Arafa Ugweno \| \|  \| 13 \| Kichemchem \| \|  \| 14 \| Sandali (Mico kasarobo old) \| \|  \| 15 \| Kibada \| \|  \| 16 \| Mji mwema \| \|  \| 17 \| Keko \| \|  \| 18 \| Kisarawe II \| \|  \| 19 \| Magomeni Health Center \| \|  \| 20 \| Kimara Dispensary \| \|  \| 21 \| Bunju Dispensary \| \|  \| 22 \| Kawe Dispensary \| \|  \| 23 \| Kijitonyama Dispensary \| \|  \| 24 \| Kinondoni Hospital - Other Hospital \| \|  \| 25 \| Makuburi Dispensary \| \|  \| 27 \| Ununio Dispensary \| \|  \| 28 \| Tandale Dispensary \| \|  \| 29 \| Mburahati Dispensary \| \|  \| 30 \| Mwenge Dispensary \| \|  \| 31 \| Mbezi Dispensary \| \|  \| 32 \| Hananasif Dispensary \| \|  \| 33 \| Kigogo Dispensary \| \|  \| 34 \| Mabibo Dispensary \| \|  \| 35 \| Goba Dispensary \| |
| \|  \| ID_study (required) \| \| --- \| --- \| | Enter the study ID-number: |  |
| \|  \| interview_date (required) \| \| --- \| --- \| | Confirm today's date: |  |
| rsp_id (required) | Counting all of the people you have interviewed today, what number survey is this? |  |
| introduction | Hello, my name is [NAME] and I am working with MDH Tanzania. The reason I am here is because we are conducting health research in Temeke district. |  |
| \|  \| time_start \| \| --- \| --- \| | Time at start of interview |  |
| \|  \| note_a \| \| --- \| --- \| | PART 1: BACKGROUND INFORMATION |  |
| \|  \| a1 (required) \| \| --- \| --- \| | WHAT IS THE RESPONDENT'S SEX?  Only ask if not obvious. | \|  \| 1 \| Female \| \| --- \| --- \| --- \| \|  \| 2 \| Male \| |
| \|  \| a2 (required) \| \| --- \| --- \| | How old are you?  Please enter the age in years. If respondent does not know, but confirms that he/she is over 18, enter 1111. |  |
| \|  \| note_a2 \| \| --- \| --- \| | The only reason why we are asking the following few questions is so that if you take the questionnaire again in the future, we can link your past responses to the new ones. |  |
| \|  \| a3 (required) \| \| --- \| --- \| | We will NOT contact you but can you please tell me the last 4 digits of your mobile phone number?  Response constrained to: regex(., '^[0-9]{4}$') |  |
| \|  \| a4 (required) \| \| --- \| --- \| | What was the name of your primary school? |  |
| \|  \| a5 (required) \| \| --- \| --- \| | What is the name of the city/town/village where you grew up? |  |
| \|  \| a6 (required) \| \| --- \| --- \| | Have you taken this questionnaire before? | \|  \| 1 \| Yes \| \| --- \| --- \| --- \| \|  \| 2 \| No \| |
| \|  \| consented_group > a7_group \| \| --- \| --- \| | | |
| \|  \|  \| a7_label \| \| --- \| --- \| --- \| | What is your professional title?  Please read out each answer option and select 'Yes' 'No' or 'REFUSED.' | \|  \| 1 \| Yes \| \| --- \| --- \| --- \| \|  \| 2 \| No \| \|  \| 99 \| REFUSED \| |
| \|  \|  \| a7_a (required) \| \| --- \| --- \| --- \| | Nurse officer | \|  \| 1 \| Yes \| \| --- \| --- \| --- \| \|  \| 2 \| No \| \|  \| 99 \| REFUSED \| |
| \|  \|  \| a7_b (required) \| \| --- \| --- \| --- \| | Nurse midwife | \|  \| 1 \| Yes \| \| --- \| --- \| --- \| \|  \| 2 \| No \| \|  \| 99 \| REFUSED \| |
| \|  \|  \| a7_c (required) \| \| --- \| --- \| --- \| | Auxiliary nurse | \|  \| 1 \| Yes \| \| --- \| --- \| --- \| \|  \| 2 \| No \| \|  \| 99 \| REFUSED \| |
| \|  \|  \| a7_d (required) \| \| --- \| --- \| --- \| | Nurse attendant | \|  \| 1 \| Yes \| \| --- \| --- \| --- \| \|  \| 2 \| No \| \|  \| 99 \| REFUSED \| |
| \|  \|  \| a7_e (required) \| \| --- \| --- \| --- \| | Public health nurse | \|  \| 1 \| Yes \| \| --- \| --- \| --- \| \|  \| 2 \| No \| \|  \| 99 \| REFUSED \| |
| \|  \|  \| a7_f (required) \| \| --- \| --- \| --- \| | Nurse counselor | \|  \| 1 \| Yes \| \| --- \| --- \| --- \| \|  \| 2 \| No \| \|  \| 99 \| REFUSED \| |
| \|  \|  \| a7_g (required) \| \| --- \| --- \| --- \| | Nutritionist | \|  \| 1 \| Yes \| \| --- \| --- \| --- \| \|  \| 2 \| No \| \|  \| 99 \| REFUSED \| |
| \|  \|  \| a7_h (required) \| \| --- \| --- \| --- \| | HBC | \|  \| 1 \| Yes \| \| --- \| --- \| --- \| \|  \| 2 \| No \| \|  \| 99 \| REFUSED \| |
| \|  \|  \| a7_i (required) \| \| --- \| --- \| --- \| | Physician | \|  \| 1 \| Yes \| \| --- \| --- \| --- \| \|  \| 2 \| No \| \|  \| 99 \| REFUSED \| |
| \|  \|  \| a7_other (required) \| \| --- \| --- \| --- \| | Other (specify on next page) | \|  \| 1 \| Yes \| \| --- \| --- \| --- \| \|  \| 2 \| No \| \|  \| 99 \| REFUSED \| |
| \|  \| a7_iother (required) \| \| --- \| --- \| | Please specify 'Other' professional title.  Question relevant when: ${a7_other} =1 |  |
| \|  \| a8_precursor (required) \| \| --- \| --- \| | Have you been working in this profession for more than one year? | \|  \| 1 \| Yes \| \| --- \| --- \| --- \| \|  \| 2 \| No \| \|  \| 99 \| REFUSED \| |
| \|  \| a8_years (required) \| \| --- \| --- \| | For how many years have you been working in this profession?  Enter -98 if DK. Enter -99 if RF.  Question relevant when: ${a8_precursor} =1  Response constrained to: .>-1 and .<121 or .=-99 or .=-98 |  |
| \|  \| a8_months (required) \| \| --- \| --- \| | For how many months have you been working in this profession?  Enter -98 if DK. Enter -99 if RF.  Question relevant when: ${a8_precursor} =2  Response constrained to: .>-1 and .<13 or .=-99 or .=-98 |  |
| \|  \| a9_precursor (required) \| \| --- \| --- \| | Have you been working in this FACILITY for more than one year? | \|  \| 1 \| Yes \| \| --- \| --- \| --- \| \|  \| 2 \| No \| \|  \| 99 \| REFUSED \| |
| \|  \| a9_years (required) \| \| --- \| --- \| | For how many years have you been working in this facility?  Enter -98 if DK. Enter -99 if RF.  Question relevant when: ${a9_precursor} =1  Response constrained to: .>0 and .<121 or .=-99 or .=-98 |  |
| \|  \| a9_months (required) \| \| --- \| --- \| | For how many months have you been working in this facility?  Enter -98 if DK. Enter -99 if RF.  Question relevant when: ${a9_precursor} =2  Response constrained to: .>-1 and .<13 or .=-99 or .=-98 |  |
| \|  \| a10 \| \| --- \| --- \| | How many patients did you see today?  Please enter the number of patients. Enter -98 if DK. Enter -99 if RF.  Response constrained to: .>-1 or .=-99 or .=-98 |  |
| \|  \| a11 \| \| --- \| --- \| | Approximately how many minutes did you spend with the last patient you saw today?  Enter -98 if DK. Enter -99 if RF.  Response constrained to: .>-1 or .=-99 or .=-98 |  |
| \|  \| a12 \| \| --- \| --- \| | On average, approximately how many minutes did you spend per patient today?  Enter -98 if DK. Enter -99 if RF.  Response constrained to: .>-1 or .=-99 or .=-98 |  |
| \|  \| b_note \| \| --- \| --- \| | PART 2: TIME PRESSURE  I would now like to ask you a few questions about the amount of time you have available to spend with clients.  Ask respondent to refer to Scale 1. |  |
| \|  \| consented_group > group_b1-6 \| \| --- \| --- \| | | |
| \|  \|  \| label_b1-6 \| \| --- \| --- \| --- \| | 1=Strongly disagree, 2=Disagree,  3=Neutral, 4=Agree, 5=Strongly agree, RF=Refused | \|  \| 1 \| 1 \| \| --- \| --- \| --- \| \|  \| 2 \| 2 \| \|  \| 3 \| 3 \| \|  \| 4 \| 4 \| \|  \| 5 \| 5 \| \|  \| RF \| Refused \| |
| \|  \|  \| b1 (required) \| \| --- \| --- \| --- \| | During the last one month, I could have provided better care to my patients if I had had more time for them. | \|  \| 1 \| 1 \| \| --- \| --- \| --- \| \|  \| 2 \| 2 \| \|  \| 3 \| 3 \| \|  \| 4 \| 4 \| \|  \| 5 \| 5 \| \|  \| RF \| Refused \| |
| \|  \|  \| b2 (required) \| \| --- \| --- \| --- \| | During the last one month, I could have easily dealt with a significantly higher number of patients. | \|  \| 1 \| 1 \| \| --- \| --- \| --- \| \|  \| 2 \| 2 \| \|  \| 3 \| 3 \| \|  \| 4 \| 4 \| \|  \| 5 \| 5 \| \|  \| RF \| Refused \| |
| \|  \|  \| b3 (required) \| \| --- \| --- \| --- \| | During the last one month, I was able to spend as much time with my patients as was necessary. | \|  \| 1 \| 1 \| \| --- \| --- \| --- \| \|  \| 2 \| 2 \| \|  \| 3 \| 3 \| \|  \| 4 \| 4 \| \|  \| 5 \| 5 \| \|  \| RF \| Refused \| |
| \|  \|  \| b4 (required) \| \| --- \| --- \| --- \| | During the last one month, I have generally had enough time to answer all my patients’ questions and concerns. | \|  \| 1 \| 1 \| \| --- \| --- \| --- \| \|  \| 2 \| 2 \| \|  \| 3 \| 3 \| \|  \| 4 \| 4 \| \|  \| 5 \| 5 \| \|  \| RF \| Refused \| |
| \|  \|  \| b5 (required) \| \| --- \| --- \| --- \| | During the last one month, I feel that my patients were probably dissatisfied with the services I provided because I was so rushed. | \|  \| 1 \| 1 \| \| --- \| --- \| --- \| \|  \| 2 \| 2 \| \|  \| 3 \| 3 \| \|  \| 4 \| 4 \| \|  \| 5 \| 5 \| \|  \| RF \| Refused \| |
| \|  \|  \| b6 (required) \| \| --- \| --- \| --- \| | During the last one month, I have been so stressed that I was sometimes impolite to my patients. | \|  \| 1 \| 1 \| \| --- \| --- \| --- \| \|  \| 2 \| 2 \| \|  \| 3 \| 3 \| \|  \| 4 \| 4 \| \|  \| 5 \| 5 \| \|  \| RF \| Refused \| |
| \|  \| c_intro \| \| --- \| --- \| | PART 3: QUALITY OF CARE  I would now like to ask you a question about quality of care. The question is NOT about the care you have provided yourself. Rather, I want to know about how you feel the quality of care is at this facility as a whole. |  |
| \|  \| c1 (required) \| \| --- \| --- \| | During the last one month, how would you say was the quality of healthcare for HIV-infected patients at this facility?  Please answer on a scale from 0 to 10 where 0 equals very bad quality of care and 10 equals very good quality of care.  Ask respondent to refer to Scale 2. | \|  \| 0 \| 0 (very bad quality of care) \| \| --- \| --- \| --- \| \|  \| 1 \| 1 \| \|  \| 2 \| 2 \| \|  \| 3 \| 3 \| \|  \| 4 \| 4 \| \|  \| 5 \| 5 \| \|  \| 6 \| 6 \| \|  \| 7 \| 7 \| \|  \| 8 \| 8 \| \|  \| 9 \| 9 \| \|  \| 10 \| 10 (very good quality of care) \| \|  \| 99 \| REFUSED \| |
| \|  \| consented_group > c2_group \| \| --- \| --- \| | | |
| \|  \|  \| c2 \| \| --- \| --- \| --- \| | What do you feel could be done to improve the quality of care for HIV patients at this facility?  Write DK if the respondent does not know. |  |
| \|  \|  \| c2_a (required) \| \| --- \| --- \| --- \| | Suggestion 1: |  |
| \|  \|  \| c2_b \| \| --- \| --- \| --- \| | Suggestion 2: |  |
| \|  \|  \| c2_c \| \| --- \| --- \| --- \| | Suggestion 3: |  |
| \|  \|  \| c2_other \| \| --- \| --- \| --- \| | Any other suggestions? | \|  \| 1 \| Yes \| \| --- \| --- \| --- \| \|  \| 2 \| No \| |
| \|  \| c2_othertext (required) \| \| --- \| --- \| | Please specify 'Other' suggestions here.  Question relevant when: ${c2_other} =1 |  |
| \|  \| d_intro \| \| --- \| --- \| | PART 4: HEALTHCARE WORKERS' VIEWS ON HOME-DELIVERY OF ART  Please read out the following introduction: |  |
| \|  \| d_note \| \| --- \| --- \| | “There is a study taking place at a few healthcare facilities in Temeke district. It tries to find out if using home-based carers to deliver ART directly to patients’ homes is a good idea. A home-based carer is someone from the community who regularly visits households to provide information on how to stay healthy and helps care for ill people at their home. Home-based carers delivering ART to patients’ homes means that patients don’t need to come to the facility to pick up their medicines. So, it may become easier for patients to stay on treatment. But it also means that ART patients see a nurse or physician only once or twice a year. We’d like to find out what you think about using home-based carers to deliver ART to patients’ homes. Please tell us if you strongly agree, agree, are neutral, disagree, or strongly disagree with the following statements.”  Ask respondent to refer to scale 1. |  |
| \|  \| consented_group > group_d1-4 \| \| --- \| --- \| | | |
| \|  \|  \| label_d1-4 \| \| --- \| --- \| --- \| | 1=Strongly disagree, 2=Disagree,  3=Neutral, 4=Agree, 5=Strongly agree, RF=Refused | \|  \| 1 \| 1 \| \| --- \| --- \| --- \| \|  \| 2 \| 2 \| \|  \| 3 \| 3 \| \|  \| 4 \| 4 \| \|  \| 5 \| 5 \| \|  \| RF \| Refused \| |
| \|  \|  \| d1 (required) \| \| --- \| --- \| --- \| | Home-based carers delivering ART to patients’ homes is a bad idea because it means that patients won’t see a nurse or physician as often. | \|  \| 1 \| 1 \| \| --- \| --- \| --- \| \|  \| 2 \| 2 \| \|  \| 3 \| 3 \| \|  \| 4 \| 4 \| \|  \| 5 \| 5 \| \|  \| RF \| Refused \| |
| \|  \|  \| d2 (required) \| \| --- \| --- \| --- \| | It is a good idea to use home-based carers to deliver ART to patients’ homes as long as the patients are asked to see a nurse or physician at least once a year. | \|  \| 1 \| 1 \| \| --- \| --- \| --- \| \|  \| 2 \| 2 \| \|  \| 3 \| 3 \| \|  \| 4 \| 4 \| \|  \| 5 \| 5 \| \|  \| RF \| Refused \| |
| \|  \|  \| d3 (required) \| \| --- \| --- \| --- \| | It is irresponsible to use home-based carers to deliver ART to patients’ homes even if patients have been stable on ART for a long time.  If the respondent asks what is meant by ‘stable’, respond with ‘a CD4-count of >350 cells/microliter at the last two measurements’. If the respondent asks what is meant by ‘a long time’, respond with ‘six months or longer’. | \|  \| 1 \| 1 \| \| --- \| --- \| --- \| \|  \| 2 \| 2 \| \|  \| 3 \| 3 \| \|  \| 4 \| 4 \| \|  \| 5 \| 5 \| \|  \| RF \| Refused \| |
| \|  \|  \| d4 (required) \| \| --- \| --- \| --- \| | Home-based carers should be used to deliver ART to patients’ homes even if the patient has only just been initiated on ART. | \|  \| 1 \| 1 \| \| --- \| --- \| --- \| \|  \| 2 \| 2 \| \|  \| 3 \| 3 \| \|  \| 4 \| 4 \| \|  \| 5 \| 5 \| \|  \| RF \| Refused \| |
| \|  \| e_intro \| \| --- \| --- \| | PART 5: JOB SATISFACTION  The following questions are about how happy you are with your job at this facility. It is important for us to find out how health workers in Dar es Salaam feel about their jobs and if this changes over time. After all, patient care tends to suffer when health workers are unhappy with their work. Please remember that all your answers will be treated as highly confidential and we will not pass any of your answers to your supervisors or anyone else outside the immediate study team. |  |
| \|  \| e1 (required) \| \| --- \| --- \| | Overall, how satisfied or dissatisfied are you with your current job? Please answer with very dissatisfied, dissatisfied, neutral, satisfied, or very satisfied. | \|  \| 1 \| Very dissatisfied \| \| --- \| --- \| --- \| \|  \| 2 \| Dissatisfied \| \|  \| 3 \| Neutral \| \|  \| 4 \| Satisfied \| \|  \| 5 \| Very satisfied \| \|  \| 99 \| REFUSED \| |
| \|  \| consented_group > group_e2-e5 \| \| --- \| --- \| | | |
| \|  \|  \| note_e2-e5 \| \| --- \| --- \| --- \| | Thinking specifically about your current job at this healthcare facility, do you agree with the following? You can answer with strongly disagree, disagree, agree, and strongly agree.  Ask respondent to refer to scale 3. |  |
| \|  \|  \| e2 (required) \| \| --- \| --- \| --- \| | The management of this healthcare facility is supportive of me. | \|  \| 1 \| Strongly disagree \| \| --- \| --- \| --- \| \|  \| 2 \| Disagree \| \|  \| 3 \| Agree \| \|  \| 4 \| Strongly agree \| \|  \| 99 \| REFUSED \| |
| \|  \|  \| e3 (required) \| \| --- \| --- \| --- \| | I receive the right amount of support and guidance from my direct supervisor. | \|  \| 1 \| Strongly disagree \| \| --- \| --- \| --- \| \|  \| 2 \| Disagree \| \|  \| 3 \| Agree \| \|  \| 4 \| Strongly agree \| \|  \| 99 \| REFUSED \| |
| \|  \|  \| e4 (required) \| \| --- \| --- \| --- \| | I am provided with all trainings necessary for me to perform my job. | \|  \| 1 \| Strongly disagree \| \| --- \| --- \| --- \| \|  \| 2 \| Disagree \| \|  \| 3 \| Agree \| \|  \| 4 \| Strongly agree \| \|  \| 99 \| REFUSED \| |
| \|  \|  \| e5 (required) \| \| --- \| --- \| --- \| | I have learned many new job skills in this position. | \|  \| 1 \| Strongly disagree \| \| --- \| --- \| --- \| \|  \| 2 \| Disagree \| \|  \| 3 \| Agree \| \|  \| 4 \| Strongly agree \| \|  \| 99 \| REFUSED \| |
| \|  \| consented_group > group_e6-e9 \| \| --- \| --- \| | | |
| \|  \|  \| note_e6-e9 \| \| --- \| --- \| --- \| | Thinking specifically about your current job at this healthcare facility, do you agree with the following? You can answer with strongly disagree, disagree, agree, and strongly agree.  Ask respondent to refer to scale 3. |  |
| \|  \|  \| e6 (required) \| \| --- \| --- \| --- \| | I feel encouraged by my supervisor to offer suggestions and improvements. | \|  \| 1 \| Strongly disagree \| \| --- \| --- \| --- \| \|  \| 2 \| Disagree \| \|  \| 3 \| Agree \| \|  \| 4 \| Strongly agree \| \|  \| 99 \| REFUSED \| |
| \|  \|  \| e7 (required) \| \| --- \| --- \| --- \| | The facility’s management makes changes based on my suggestions and feedback. | \|  \| 1 \| Strongly disagree \| \| --- \| --- \| --- \| \|  \| 2 \| Disagree \| \|  \| 3 \| Agree \| \|  \| 4 \| Strongly agree \| \|  \| 99 \| REFUSED \| |
| \|  \|  \| e8 (required) \| \| --- \| --- \| --- \| | I am appropriately recognized when I perform well at my regular work duties. | \|  \| 1 \| Strongly disagree \| \| --- \| --- \| --- \| \|  \| 2 \| Disagree \| \|  \| 3 \| Agree \| \|  \| 4 \| Strongly agree \| \|  \| 99 \| REFUSED \| |
| \|  \|  \| e9 (required) \| \| --- \| --- \| --- \| | The facility rules make it easy for me to do a good job. | \|  \| 1 \| Strongly disagree \| \| --- \| --- \| --- \| \|  \| 2 \| Disagree \| \|  \| 3 \| Agree \| \|  \| 4 \| Strongly agree \| \|  \| 99 \| REFUSED \| |
| \|  \| consented_group > group_e10-e13 \| \| --- \| --- \| | | |
| \|  \|  \| note_e10-e13 \| \| --- \| --- \| --- \| | Thinking specifically about your current job at this healthcare facility, do you agree with the following? You can answer with strongly disagree, disagree, agree, and strongly agree.  Ask respondent to refer to scale 3. |  |
| \|  \|  \| e10 (required) \| \| --- \| --- \| --- \| | I am satisfied with my chances for promotion. | \|  \| 1 \| Strongly disagree \| \| --- \| --- \| --- \| \|  \| 2 \| Disagree \| \|  \| 3 \| Agree \| \|  \| 4 \| Strongly agree \| \|  \| 99 \| REFUSED \| |
| \|  \|  \| e11 (required) \| \| --- \| --- \| --- \| | I have adequate opportunities to develop my professional skills. | \|  \| 1 \| Strongly disagree \| \| --- \| --- \| --- \| \|  \| 2 \| Disagree \| \|  \| 3 \| Agree \| \|  \| 4 \| Strongly agree \| \|  \| 99 \| REFUSED \| |
| \|  \|  \| e12 (required) \| \| --- \| --- \| --- \| | I have an accurate written job description. | \|  \| 1 \| Strongly disagree \| \| --- \| --- \| --- \| \|  \| 2 \| Disagree \| \|  \| 3 \| Agree \| \|  \| 4 \| Strongly agree \| \|  \| 99 \| REFUSED \| |
| \|  \|  \| e13 (required) \| \| --- \| --- \| --- \| | The amount of work I am expected to finish each week is reasonable. | \|  \| 1 \| Strongly disagree \| \| --- \| --- \| --- \| \|  \| 2 \| Disagree \| \|  \| 3 \| Agree \| \|  \| 4 \| Strongly agree \| \|  \| 99 \| REFUSED \| |
| \|  \| consented_group > group_e14-e18 \| \| --- \| --- \| | | |
| \|  \|  \| note_e14-e18 \| \| --- \| --- \| --- \| | Thinking specifically about your current job at this healthcare facility, do you agree with the following? You can answer with strongly disagree, disagree, agree, and strongly agree.  Ask respondent to refer to scale 3. |  |
| \|  \|  \| e14 (required) \| \| --- \| --- \| --- \| | My work assignments are always clearly explained to me. | \|  \| 1 \| Strongly disagree \| \| --- \| --- \| --- \| \|  \| 2 \| Disagree \| \|  \| 3 \| Agree \| \|  \| 4 \| Strongly agree \| \|  \| 99 \| REFUSED \| |
| \|  \|  \| e15 (required) \| \| --- \| --- \| --- \| | My work is evaluated based on a fair system of performance standards. | \|  \| 1 \| Strongly disagree \| \| --- \| --- \| --- \| \|  \| 2 \| Disagree \| \|  \| 3 \| Agree \| \|  \| 4 \| Strongly agree \| \|  \| 99 \| REFUSED \| |
| \|  \|  \| e16 (required) \| \| --- \| --- \| --- \| | This facility provides all the equipment, supplies, and resources necessary for me to perform my duties. | \|  \| 1 \| Strongly disagree \| \| --- \| --- \| --- \| \|  \| 2 \| Disagree \| \|  \| 3 \| Agree \| \|  \| 4 \| Strongly agree \| \|  \| 99 \| REFUSED \| |
| \|  \|  \| e17 (required) \| \| --- \| --- \| --- \| | My co-workers and I work well together. | \|  \| 1 \| Strongly disagree \| \| --- \| --- \| --- \| \|  \| 2 \| Disagree \| \|  \| 3 \| Agree \| \|  \| 4 \| Strongly agree \| \|  \| 99 \| REFUSED \| |
| \|  \|  \| e18 (required) \| \| --- \| --- \| --- \| | I feel I can easily communicate with members from all levels of this facility. | \|  \| 1 \| Strongly disagree \| \| --- \| --- \| --- \| \|  \| 2 \| Disagree \| \|  \| 3 \| Agree \| \|  \| 4 \| Strongly agree \| \|  \| 99 \| REFUSED \| |
| \|  \| e19 (required) \| \| --- \| --- \| | For the next question, please answer with definitely no, probably no, probably yes, or definitely yes.  I would recommend this health facility to other workers as a good place to work. | \|  \| 1 \| Definitely no \| \| --- \| --- \| --- \| \|  \| 2 \| Probably no \| \|  \| 3 \| Probably yes \| \|  \| 4 \| Definitely yes \| \|  \| 99 \| REFUSED \| |
| \|  \| e20 (required) \| \| --- \| --- \| | How would you rate this health facility as a place to work on a scale from 0 (the worst) to 10 (the best)?  Ask respondent to refer to Scale 6. | \|  \| 0 \| 0 (Very dissatisfied) \| \| --- \| --- \| --- \| \|  \| 1 \| 1 \| \|  \| 2 \| 2 \| \|  \| 3 \| 3 \| \|  \| 4 \| 4 \| \|  \| 5 \| 5 \| \|  \| 6 \| 6 \| \|  \| 7 \| 7 \| \|  \| 8 \| 8 \| \|  \| 9 \| 9 \| \|  \| 10 \| 10 (Very satisfied) \| \|  \| 99 \| REFUSED \| |
| \|  \| consented_group > group_e21-e24 \| \| --- \| --- \| | | |
| \|  \|  \| note_e21-e24 \| \| --- \| --- \| --- \| | Thinking specifically about your current job at this facility, do you agree with the following? You can answer with strongly disagree, disagree, neutral, agree and strongly agree.  Ask the respondent to refer to scale 1. |  |
| \|  \|  \| e21 (required) \| \| --- \| --- \| --- \| | I find real enjoyment in my job. | \|  \| 1 \| Strongly disagree \| \| --- \| --- \| --- \| \|  \| 2 \| Disagree \| \|  \| 3 \| Neutral \| \|  \| 4 \| Agree \| \|  \| 5 \| Strongly agree \| \|  \| 99 \| REFUSED \| |
| \|  \|  \| e22 (required) \| \| --- \| --- \| --- \| | I like my job better than the average person. | \|  \| 1 \| Strongly disagree \| \| --- \| --- \| --- \| \|  \| 2 \| Disagree \| \|  \| 3 \| Neutral \| \|  \| 4 \| Agree \| \|  \| 5 \| Strongly agree \| \|  \| 99 \| REFUSED \| |
| \|  \|  \| e23 (required) \| \| --- \| --- \| --- \| | Most days I am enthusiastic about my job. | \|  \| 1 \| Strongly disagree \| \| --- \| --- \| --- \| \|  \| 2 \| Disagree \| \|  \| 3 \| Neutral \| \|  \| 4 \| Agree \| \|  \| 5 \| Strongly agree \| \|  \| 99 \| REFUSED \| |
| \|  \|  \| e24 (required) \| \| --- \| --- \| --- \| | I feel fairly well satisfied with my job. | \|  \| 1 \| Strongly disagree \| \| --- \| --- \| --- \| \|  \| 2 \| Disagree \| \|  \| 3 \| Neutral \| \|  \| 4 \| Agree \| \|  \| 5 \| Strongly agree \| \|  \| 99 \| REFUSED \| |
| \|  \| consented_group > group_e25-e28 \| \| --- \| --- \| | | |
| \|  \|  \| note_e25-e28 \| \| --- \| --- \| --- \| | Thinking specifically about your current job at this facility, do you agree with the following? You can answer with strongly disagree, disagree, neutral, agree and strongly agree.  Ask the respondent to refer to scale 1. |  |
| \|  \|  \| e25 (required) \| \| --- \| --- \| --- \| | I am willing to put in a great deal of effort to make this facility successful. | \|  \| 1 \| Strongly disagree \| \| --- \| --- \| --- \| \|  \| 2 \| Disagree \| \|  \| 3 \| Neutral \| \|  \| 4 \| Agree \| \|  \| 5 \| Strongly agree \| \|  \| 99 \| REFUSED \| |
| \|  \|  \| e26 (required) \| \| --- \| --- \| --- \| | These days I feel motivated to work as hard as I can. | \|  \| 1 \| Strongly disagree \| \| --- \| --- \| --- \| \|  \| 2 \| Disagree \| \|  \| 3 \| Neutral \| \|  \| 4 \| Agree \| \|  \| 5 \| Strongly agree \| \|  \| 99 \| REFUSED \| |
| \|  \|  \| e27 (required) \| \| --- \| --- \| --- \| | I am careful not to make errors at work. | \|  \| 1 \| Strongly disagree \| \| --- \| --- \| --- \| \|  \| 2 \| Disagree \| \|  \| 3 \| Neutral \| \|  \| 4 \| Agree \| \|  \| 5 \| Strongly agree \| \|  \| 99 \| REFUSED \| |
| \|  \|  \| e28 (required) \| \| --- \| --- \| --- \| | When I am not sure how to treat a client’s condition I look for information or ask for advice. | \|  \| 1 \| Strongly disagree \| \| --- \| --- \| --- \| \|  \| 2 \| Disagree \| \|  \| 3 \| Neutral \| \|  \| 4 \| Agree \| \|  \| 5 \| Strongly agree \| \|  \| 99 \| REFUSED \| |
| \|  \| f_intro \| \| --- \| --- \| | PART 6: QUESTIONS ON INTENT TO LEAVE THE JOB |  |
| \|  \| consented_group > group_f1-f3 \| \| --- \| --- \| | | |
| \|  \|  \| note_f1-f3 \| \| --- \| --- \| --- \| | When people are dissatisfied with their job, they tend to look for other options. The following questions are about how often you are thinking about other job options. Again, none of this information will be shared with anyone outside the immediate study team.  Please ask respondent to refer to scale 4. |  |
| \|  \|  \| f1 (required) \| \| --- \| --- \| --- \| | How frequently do you think about leaving this facility to work somewhere else? | \|  \| 1 \| Never \| \| --- \| --- \| --- \| \|  \| 2 \| Rarely \| \|  \| 3 \| Occasionally \| \|  \| 4 \| Often \| \|  \| 5 \| Very often \| \|  \| 99 \| REFUSED \| |
| \|  \|  \| f2 (required) \| \| --- \| --- \| --- \| | How frequently do you think about finding a job that does not involve working with patients? | \|  \| 1 \| Never \| \| --- \| --- \| --- \| \|  \| 2 \| Rarely \| \|  \| 3 \| Occasionally \| \|  \| 4 \| Often \| \|  \| 5 \| Very often \| \|  \| 99 \| REFUSED \| |
| \|  \|  \| f3 (required) \| \| --- \| --- \| --- \| | How frequently do you think about leaving Tanzania to work abroad? | \|  \| 1 \| Never \| \| --- \| --- \| --- \| \|  \| 2 \| Rarely \| \|  \| 3 \| Occasionally \| \|  \| 4 \| Often \| \|  \| 5 \| Very often \| \|  \| 99 \| REFUSED \| |
| \|  \| consented_group > group_f4-f6 \| \| --- \| --- \| | | |
| \|  \|  \| note_f4-f6 \| \| --- \| --- \| --- \| | For the following few questions, please answer with very unlikely, unlikely, uncertain, likely, or very likely.  Please ask the respondent to refer to scale 5. |  |
| \|  \|  \| f4 (required) \| \| --- \| --- \| --- \| | How likely is it that you will search for a job outside this facility? | \|  \| 1 \| Very unlikely \| \| --- \| --- \| --- \| \|  \| 2 \| Unlikely \| \|  \| 3 \| Uncertain \| \|  \| 4 \| Likely \| \|  \| 5 \| Very likely \| \|  \| 99 \| REFUSED \| |
| \|  \|  \| f5 (required) \| \| --- \| --- \| --- \| | How likely is it that you will actually leave this facility in the next 5 years? | \|  \| 1 \| Very unlikely \| \| --- \| --- \| --- \| \|  \| 2 \| Unlikely \| \|  \| 3 \| Uncertain \| \|  \| 4 \| Likely \| \|  \| 5 \| Very likely \| \|  \| 99 \| REFUSED \| |
| \|  \|  \| f6 (required) \| \| --- \| --- \| --- \| | How likely is it that you will search for a job that does not involve working with patients? | \|  \| 1 \| Very unlikely \| \| --- \| --- \| --- \| \|  \| 2 \| Unlikely \| \|  \| 3 \| Uncertain \| \|  \| 4 \| Likely \| \|  \| 5 \| Very likely \| \|  \| 99 \| REFUSED \| |
| \|  \| consented_group > group_f7-f9 \| \| --- \| --- \| | | |
| \|  \|  \| note_f7-f9 \| \| --- \| --- \| --- \| | For the following few questions, please answer with very unlikely, unlikely, uncertain, likely, or very likely.  Please ask the respondent to refer to scale 5. |  |
| \|  \|  \| f7 (required) \| \| --- \| --- \| --- \| | Within the next 5 years, how likely is it that you will actually leave this job to take up a job that does not involve working patients? | \|  \| 1 \| Very unlikely \| \| --- \| --- \| --- \| \|  \| 2 \| Unlikely \| \|  \| 3 \| Uncertain \| \|  \| 4 \| Likely \| \|  \| 5 \| Very likely \| \|  \| 99 \| REFUSED \| |
| \|  \|  \| f8 (required) \| \| --- \| --- \| --- \| | How likely is it that you will search for a job abroad? | \|  \| 1 \| Very unlikely \| \| --- \| --- \| --- \| \|  \| 2 \| Unlikely \| \|  \| 3 \| Uncertain \| \|  \| 4 \| Likely \| \|  \| 5 \| Very likely \| \|  \| 99 \| REFUSED \| |
| \|  \|  \| f9 (required) \| \| --- \| --- \| --- \| | How likely is it that you will actually leave Tanzania to work abroad within the next 5 years? | \|  \| 1 \| Very unlikely \| \| --- \| --- \| --- \| \|  \| 2 \| Unlikely \| \|  \| 3 \| Uncertain \| \|  \| 4 \| Likely \| \|  \| 5 \| Very likely \| \|  \| 99 \| REFUSED \| |
| \|  \| time_end \| \| --- \| --- \| | Time at end of interview |  |
| \|  \| respondent_comments \| \| --- \| --- \| | Thank you very much for your effort and time!  Do you have any comments or feedback for us? |  |
